# KAT2A-driven succinylation of SRSF11 enforces spliceosome-mediated RAD52 splicing to promote homologous recombination and radioresistance in hepatocellular carcinoma

**DOI:** 10.1038/s41392-025-02458-7

**Published:** 2025-11-07

**Authors:** Jun Wu, Jingsheng Yuan, Zijian Liu, Yongjie Zhou, Bo Zhang, Yahong Xu, Qiwen Zeng, Zhenru Wu, Lingxiang Kong, Jiaguo Wang, Bohan Zhang, Jian Yang, Tao Lv, Yujun Shi, Jiayin Yang

**Affiliations:** 1 Department of Liver Transplantation Center & Institute of Organ Transplantation, West China Hospital, Sichuan University, Chengdu, China; 2 Key Laboratory of Transplant Engineering and Immunology, NHC, West China Hospital, Sichuan University, Chengdu, China; 3 Department of Head and Neck Oncology and Department of Radiation Oncology, Cancer Center, West China Hospital, Sichuan University, Chengdu, China; 4 Department of Critical Care Medicine, West China Hospital, Sichuan University, Chengdu, China; 5 Department of General Surgery and NHC Key Laboratory of Transplant Engineering and Immunology, Frontiers Science Center for Disease-related Molecular Network, West China Hospital, Sichuan University, Chengdu, China; 6 Institute of Clinical Pathology, West China Hospital, Sichuan University, Chengdu, China; 7 Department of Plastic and Burn Surgery, West China Hospital, Sichuan University, Chengdu, China

**Keywords:** Gastrointestinal cancer, Oncogenes

## Abstract

Posttranslational modification succinylation plays a pivotal role in tumorigenesis across malignancies, yet its mechanistic contributions to hepatocellular carcinoma (HCC) pathogenesis and therapeutic resistance remain poorly characterized. In this study, we systematically demonstrated that the splicing factor SRSF11 undergoes functional consequential succinylation in HCC progression. Mechanistically, lysine acetyltransferase 2 A (KAT2A) directly interacts with SRSF11 to catalyze its succinylation at lysine 419 (K419), thereby enhancing DNA damage repair capacity in both in vitro and in vivo HCC models. Structural and functional analyses revealed that K419 succinylation stabilizes SRSF11-spliceosome interactions, which promote the inclusion of exon 10 of RAD52 through enhanced pre-mRNAs binding. This exon-specific splicing event preserves the RAD51-binding domain essential for homologous recombination (HR) repair, ultimately facilitating RAD52-RAD51 dimer assembly and HR-mediated genomic stabilization. Clinically, elevated SRSF11 expression is correlated with increased HR activity, radioresistance, and reduced survival in HCC patients. Notably, genetic disruption of the KAT2A-SRSF11 axis sensitizes HCC cells to radiation-induced apoptosis. Our findings establish succinylation as a novel regulatory mechanism linking alternative splicing to DNA repair fidelity in HCC, while proposing therapeutic targeting of this pathway to overcome radioresistance in advanced HCC.

## Introduction

Hepatocellular carcinoma (HCC), the predominant form of primary liver cancer, ranks as the third-leading cause of global cancer mortality, with 5-year survival rates less than 20% due to frequent late-stage diagnosis and postoperative recurrence.^1,2^ Although combinatorial immunotherapy and antiangiogenic regimens have improved outcomes, the 4-year overall survival rate remains limited to 25.2%.^3,4^ In recent years, radiotherapeutic strategies—notably stereotactic body radiotherapy and yttrium-90 radioembolization—demonstrated efficacy in reducing recurrence and prolonging survival for advanced HCC patients with macrovascular invasion or metastatic disease.^5–8^ Nevertheless, intrinsic resistance restricts durable clinical responses, underscoring the imperative to delineate novel resistance mechanisms and therapeutic vulnerabilities.

Genomic instability, a cancer hallmark driven by defective DNA damage response (DDR), enables malignant progression through aberrant cell cycle control and error-prone repair.^9–11^ Among DNA lesions, double-strand breaks (DSBs) pose the most severe threat to genomic integrity, engaging two principal repair pathways: nonhomologous end joining (NHEJ) and homologous recombination (HR).^12,13^ NHEJ is a rapid process that does not involve the use of a homologous template for repair.^12,13^ In contrast, HR, restricted to the S/G2 phase, ensures high-fidelity repair via RAD51-mediated homology-directed synthesis, which requires BRCA1/2-dependent RAD51 filament assembly and RAD52-facilitated strand invasion.^12–18^ However, tumor cells exploit these DDR pathways to evade radiation-induced lethality, yet the regulatory mechanisms governing repair fidelity remain incompletely resolved.

Emerging evidence implicates succinylation—a conserved posttranslational modification (PTM) involving lysine residue conjugation with succinyl groups—as a critical regulator of oncogenic processes and an important potential therapeutic target of cancer.^19–21^ This dynamic modification is orchestrated by succinyltransferases and desuccinylases, such as carnitine palmitoyltransferase 1A (CPT1A), K(lysine) acetyltransferase 2A (KAT2A), histone acetyltransferase 1 (HAT1) and P300.^21,22^ Due to substantial mass/charge perturbations, succinylation induces profound structural and functional protein alterations.^21,23^ In recent years, many studies have shown that the process of DNA damage repair is strictly regulated by PTMs in cancers.^24,25^ While a recent study links succinylation to nucleosome destabilization during DDR,^26^ its mechanistic interplay with DSBs repair pathways in therapeutic resistance remains unexplored.

Building on our prior identification of serine/arginine-rich splicing factor 11 (SRSF11) as an HCC proliferation regulator,^27^ we herein demonstrated that SRSF11 serves as a pivotal succinylation substrate in HCC. Mechanistically, KAT2A catalyzes SRSF11 succinylation at lysine 419 (K419), enhancing spliceosome recruitment to enforce RAD52 exon 10 inclusion. This splicing event preserves the RAD51-binding domain, enabling RAD52-RAD51 dimerization to drive HR-mediated repair and radioresistance. Genetic disruption of the KAT2A-SRSF11 axis induces RAD52 exon 10 skipping, abrogates the RAD51 interaction, and sensitizes HCC cells to radiation. Our work establishes succinylation as a master regulator of splicing-mediated DDR in HCC while nominating the KAT2A-SRSF11-RAD52 circuit as a therapeutic target to overcome radioresistance.

## Results

### KAT2A drives oncogenic SRSF11 succinylation at K419 in HCC

**Fig. 1:**
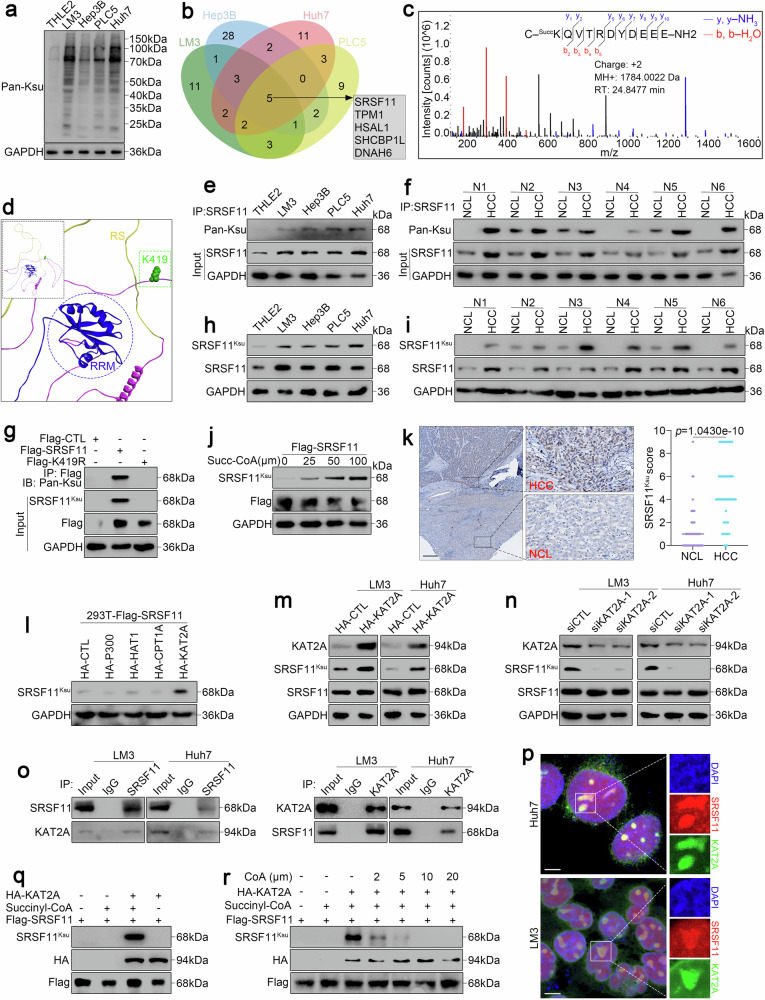
KAT2A drives oncogenic SRSF11 succinylation at K419. **a** The succinylation level examined with anti-pan-Ksu antibody in LM3, Hep3B, PLC5, Huh7 and THLE2 cells. **b** Venn diagram showing the overlapping succinylated proteins detected by LC‒MS/MS in LM3, Hep3B, PLC5 and Huh7 cells. **c** LC‒MS/MS spectrum of the succinylated site at K419 of SRSF11. **d** Schematic overview of SRSF11 K419 succinylation. The succinylation level and expression of SRSF11 examined with anti-pan-Ksu antibody in HCC cells and THLE2 cells (**e**), together with HCC and NCL tissues (**f**, N: the clinical samples from different patients) after immunoprecipitation with an anti-SRSF11 antibody. **g** The succinylation level of SRSF11 in 293 T cells was examined after overexpression of Flag-SRSF11 and Flag-SRSF11 K419R and immunoprecipitation with an anti-Flag antibody. The succinylation level and expression of SRSF11 examined with anti-SRSF11^Ksu^ antibody that specifically recognized SRSF11 with succinylation at K419 in HCC cells and THLE2 cells (**h**), together with HCC and NCL tissues (**i**). **j** Purified Flag-SRSF11 protein was incubated with the indicated concentrations of succinyl CoA. The succinylation level of SRSF11 examined with anti-SRSF11^Ksu^ antibody. **k** Representative images of IHC staining with anti-SRSF11^Ksu^ antibody in NCL and HCC tissues. Scale bar, 200 μm. **l** The succinylation level of Flag-SRSF11-overexpressed 293 T cells by overexpression of HA-P300, HAT1, CPT1A or KAT2A. Succinylation and expression levels of SRSF11 in Huh7 and LM3 cells after overexpression of HA-KAT2A (**m**) and knockdown of KAT2A by siRNA transfection (**n**). **o**. The binding of KAT2A with SRSF11 detected by anti-KAT2A antibody after immunoprecipitation with an anti-SRSF11 antibody (left), and the binding of SRSF11 with KAT2A detected by anti-SRSF11 antibody after immunoprecipitation with an anti-KAT2A antibody (right) in Huh7 and LM3 cells. **p** Immunofluorescence staining showing the colocalization of SRSF11 with KAT2A in Huh7 and LM3 cells. Scale bar, 5 μm. **q** Purified Flag-SRSF11 protein was incubated with HA-KAT2A and the indicated concentrations of succinyl-CoA. The succinylation level of SRSF11 examined with anti-SRSF11^Ksu^ antibody. **r** Purified Flag-SRSF11 protein was incubated with HA-KAT2A at the indicated concentrations of succinyl CoA and CoA. The succinylation level of SRSF11 examined with anti-SRSF11^Ksu^ antibody. For k, the data are presented as the means ± SDs. For **a**, **e**‒**j** and **l**‒**r**, 3 independent experiments (*n* = 3) with similar results were performed in triplicate. In k, the statistical analyses were performed via two-tailed unpaired Student’s *t*-tests. p < 0.05 was considered to indicate statistical significance

To screen for critical succinylated candidates in HCC, we first detected the global succinylation levels using an anti-pan-lysine succinylation (Ksu) sites antibody in HCC cell lines (LM3, Hep3B, PLC5, Huh7) and immortal adult liver epithelial THLE2 cells. The global succinylation detection revealed pronounced hyper-succinylation in HCC cell lines relative to THLE2 cells (Fig. 1a). To obtain the comprehensive insights of succinylation modifications in HCC, we detected succinylated residues in LM3, Hep3B, PLC5, and Huh7 cells via liquid chromatography‒tandem mass spectrometry (LC‒MS/MS) analysis. LC-MS/MS-based proteomic profiling identified five consistently succinylated targets across HCC cells, namely, SRSF11, TPM1, HSAL1, SHCBP1L and DNAH6 (Fig. 1b and Supplementary Table 1). Given the role of SRSF11 in HCC pathobiology established in our previous study,^27^ it is prioritized for further functional interrogation. LC‒MS/MS analysis resolved K419 as the principal succinylation site on SRSF11 (Fig. 1c). Moreover, analysis of different species revealed that the K419 residue of SRSF11 is highly conserved from *Homo sapiens* to *Bos taurus* (Supplementary Fig. 1a). Alternatively, we displayed a schematic overview of SRSF11 K419 succinylation by bioinformatics using Discovery Studio (Fig. 1d). Notably, the K419 residue is adjacent to the RNA recognition motif (RRM) domain and the arginine-serine (RS) domain, suggesting that K419 succinylation of SRSF11 may participate in the regulation of SRSF11 interactions with downstream RNAs or other splicing factors.

To further validate and confirm the site-specific succinylation of SRSF11, four methods were employed. First, total protein extracts from HCC cells and THLE2 cells were immunoprecipitated with an anti-SRSF11 antibody, followed by western blotting with an anti-pan-Ksu antibody. The results indicated that the level of SRSF11 succinylation was markedly increased in HCC cells compared with THLE2 cells (Fig. 1e and Supplementary Fig. 1b). Consistently, the SRSF11 succinylation level was markedly increased in HCC tissues compared with noncancerous liver (NCL) tissues in human (Fig. 1f and Supplementary Fig. 1c) and mouse primary HCC model (Supplementary Fig. 1d). Then, we mutated the lysine at K419 to arginine (K419R) of SRSF11 and expressed Flag-tagged wild-type SRSF11 (Flag-SRSF11) and mutant SRSF11 (Flag-SRSF11 K419R) in 293 T cells. The results revealed that K419R substitution abrogated succinylation signals in 293 T overexpression models (Fig. 1g). Third, we generated an antibody that specifically recognizes SRSF11 with succinylation at K419 (anti-SRSF11^Ksu^). We further verified the succinylation of SRSF11 K419 in the above HCC cells and tissues using anti-SRSF11^Ksu^ antibody. As expected, the examining results for K419 succinylation with the anti-SRSF11^Ksu^ antibody were consistent with those with the anti-pan-Ksu antibody (Fig. 1h, i and Supplementary Fig. 1e, f). Finally, to determine whether K419 of SRSF11 is succinylated in vitro, we incubated purified Flag-SRSF11 with succinyl-CoA at concentrations ranging from 0–100 μM, and dose-responsive K419 succinylation of recombinant SRSF11 occurred with succinyl-CoA supplementation (Fig. 1j). In addition, immunohistochemistry (IHC) assays with the anti-SRSF11^Ksu^ antibody further revealed that, compared with that in NCL tissues, the K419 succinylation of SRSF11 is markedly elevated in HCC tissues (Fig. 1k). These results showed that SRSF11 is succinylated at K419 in HCC.

To further investigate the succinyltransferase that succinylates SRSF11 at K419, we overexpressed several identified succinyltransferases (P300, HAT1, CPT1A, KAT2A) in 293 T cells. The results revealed that KAT2A was the exclusive catalyst of SRSF11 K419 modification (Fig. 1l). We subsequently overexpressed and knocked down KAT2A in LM3 and Huh7 cells, respectively. We found that overexpression of KAT2A can enhance the K419 succinylation of SRSF11 (Fig. 1m) and that knockdown of KAT2A by siRNAs reduces SRSF11 succinylation at K419 (Fig. 1n). Notably, neither the overexpression nor the knockdown of KAT2A affected the protein level of SRSF11 (Fig. 1m, n). These results suggest that K419 succinylation may modulate SRSF11 functionality independent of protein stability. Next, we confirmed the direct binding of SRSF11 and KAT2A through coimmunoprecipitation (Co-IP) in LM3 and Huh7 cells (Fig. 1o). Immunofluorescence revealed that SRSF11 colocalized with KAT2A in the nucleus (Fig. 1p). In situ Proximity Ligation Assay (PLA) further supported the interaction of SRSF11 with KAT2A (Supplementary Fig. 1g). To test whether KAT2A can succinylate SRSF11 through succinyl CoA in vitro, we incubated purified Flag-SRSF11 and succinyl CoA with or without KAT2A, and the results showed that SRSF11 could be succinylated only in the presence of KAT2A (Fig. 1q). Notably, KAT2A-mediated SRSF11 succinylation was inhibited by high-dose CoA (Fig. 1r), suggesting that CoA competes with succinyl-CoA to bind to KAT2A and that the CoA group in succinyl-CoA is involved in its interaction with KAT2A. In short, these data confirm that KAT2A directly succinylates SRSF11 at K419 in HCC.

### SRSF11 depletion exacerbates genomic instability and enhances radiosensitivity

**Fig. 2:**
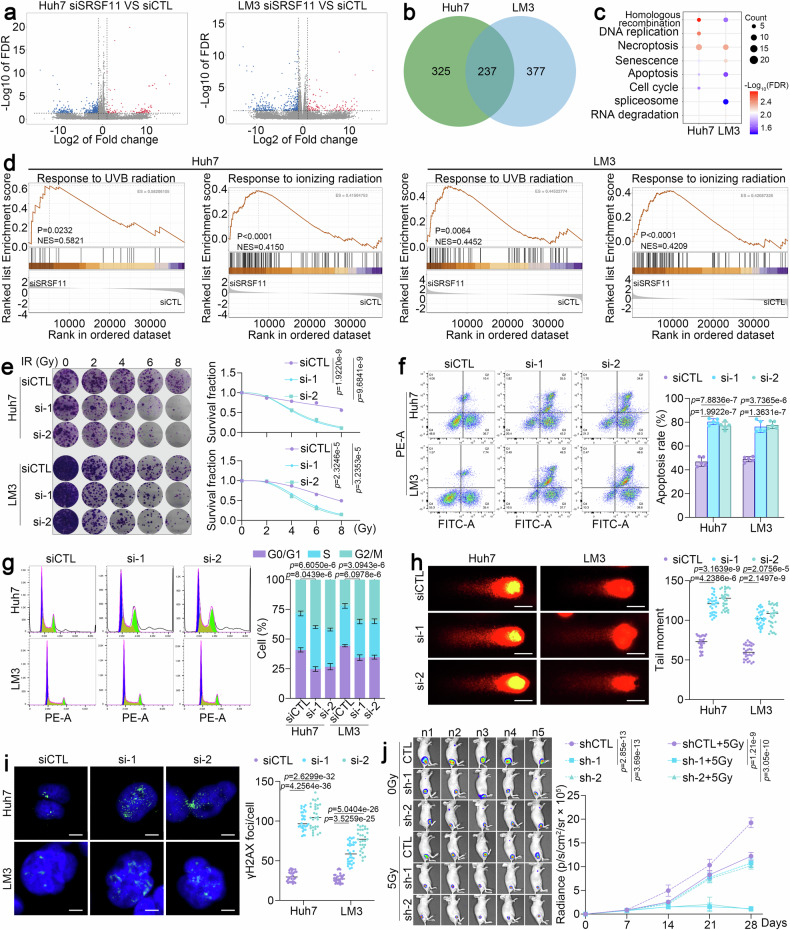
SRSF11 inhibition increases DNA damage and radiotherapy sensitivity. **a** Volcano plot showing the differentially expressed genes by knockdown of SRSF11 in Huh7 (left) and LM3 (right) cells. Fold change ≥ 1.5 and FDR value < 0.05 were considered to indicate significance. **b** Venn diagram showing overlapping differentially expressed genes in both Huh7 and LM3 cells. **c** KEGG analysis of differentially expressed genes in Huh7 and LM3 cells. **d**. GSEA of Huh7 (left) and LM3 (right) cells. **e** Colony formation assays of LM3 and Huh7 cells transfected with siSRSF11 and treated with the indicated dose of IR. Flow cytometry analysis of apoptosis (**f**) and cell cycle (**g**) in Huh7 and LM3 cells transfected with siSRSF11 and treated with 5 Gy of IR. **h** Comet assay of Huh7 and LM3 cells transfected with siSRSF11 and treated with 5 Gy of IR. Scale bars, 10 μm. **i** Immunofluorescence staining for γ-H2AX (green) and DAPI (blue) in LM3 and Huh7 cells transfected with siSRSF11 and treated with 5 Gy of IR. Scale bars, 10 μm. **j** Luciferase signals of xenograft model in nude mice using LM3 cells transfected with shSRSF11 and treated without/with IR. (*n* = 5 per group). For **e**‒**j**, the data are presented as the means ± SDs. For **e**‒**i**, 3 independent experiments (*n* = 3) with similar results were performed in triplicate. For **e**‒**j**, the statistical analyses were performed via two-tailed unpaired Student’s *t*-tests. *p* < 0.05 was considered to indicate statistical significance

To further explore the potential role of SRSF11 in HCC, we comprehensively investigated the function of SRSF11 through RNA sequencing (RNA-seq). Principal component analysis revealed that SRSF11 knockdown led to global transcriptome alterations (Supplementary Fig. 2a). 214 upregulated genes and 348 downregulated genes were identified in Huh7 cells and 233 upregulated genes and 381 downregulated genes were identified in LM3 cells (FDR < 0.05, fold change ≥ 1.5) (Fig. 2a). Among them, 237 dysregulated genes were observed across LM3 and Huh7 models (Fig. 2b). Pathway Enrichment Analysis (KEGG) enrichment analysis of these dysregulated genes revealed that silenced SRSF11 is associated with cell damage-related signaling pathways, such as necroptosis, apoptosis and RNA degradation (Fig. 2c). Further gene set enrichment analysis (GSEA) using all differentially expressed genes from each cell line suggested that the response to UVB radiation, ionizing radiation (IR) and HR were strongly enriched (Fig. 2d and Supplementary Fig. 2b). Thus, we deduce that SRSF11 participates in genomic instability responses and regulating radiotherapy sensitivity in HCC.

To corroborate the above deduction, we knocked down SRSF11 in Huh7 and LM3 cells via siRNAs (Supplementary Fig. 2c) and then treated the cells with IR. A single-hit multitarget model confirmed that SRSF11 knockdown markedly suppressed HCC cell proliferation treated by IR (Fig. 2e). Flow cytometric analysis further confirmed that SRSF11 silencing significantly increased IR-induced apoptosis (Fig. 2f). An increase in the proportion of cells in the G2/M phase indicates that the cells are more sensitive to IR.^13^ We found that inhibiting SRSF11 markedly increased G2/M phase arrest in HCC cells treated with IR (Fig. 2g). DSBs constitute a critical mechanism for IR-induced cell lethality.^13^ We next assessed IR-induced cellular DSBs by quantifying comet tail moments and phosphorylated histone H2AX (γH2AX) foci. The comet assay results indicated that SRSF11-silenced HCC cells presented more severe DSBs than did the corresponding control cells after IR treatment (Fig. 2h). Consistently, knockdown of SRSF11 significantly increased the number of γH2AX foci induced by IR (Fig. 2i). To further validate the above results, we constructed lentiviruses containing shRNAs targeting SRSF11 (Supplementary Fig. 2d) and found that SRSF11 inhibition indeed significantly promoted IR-induced cell death and DNA damage in HCC (Supplementary Fig. 2e-i). In addition, knockout of SRSF11 by sgRNA (Supplementary Fig. 2j) showed the consistent results (Supplementary Fig. 2k, l). Importantly, an in vivo xenograft model further demonstrated the significant synergistic inhibitory effect of SRSF11 knockdown after IR in HCC, as reflected by results for tumor growth and tumor weight (Fig. 2j and Supplementary Fig. 2m). These findings establish SRSF11 as a critical modulator of DNA damage repair and radioresistance in HCC.

### SRSF11 enhances homologous recombination-mediated DNA repair

Next, we further explored the effects of SRSF11 overexpression on DNA damage and radiosensitivity. To this end, we constructed an SRSF11 expression vector containing tetracycline-induced SRSF11 silencing (Tet-off) elements. As anticipated, overexpression of SRSF11 in HCC cells promoted cell proliferation and survival upon IR, whereas TET treatment-induced SRSF11 re-silencing markedly exacerbated this severe impairment (Supplementary Fig. 3a, b). Analysis of the cell cycle showed that overexpressing SRSF11 reduced the proportion of cells in the G2/M phase, whereas silencing SRSF11 by TET treatment increased the proportion of cells in the G2/M phase compared with that in the control group (Supplementary Fig. 3c). The quantification of comet tail moments and γH2AX foci further revealed that overexpression of SRSF11 mitigated DNA DSBs (Supplementary Fig. 3d, e). Consistent with the cellular data, xenograft models corroborated these findings, with SRSF11 overexpression abrogating radiation-induced tumor suppression, whereas TET-induced silencing of SRSF11 recaptured the effect of IR (Supplementary Fig. 3f, i).

**Fig. 3:**
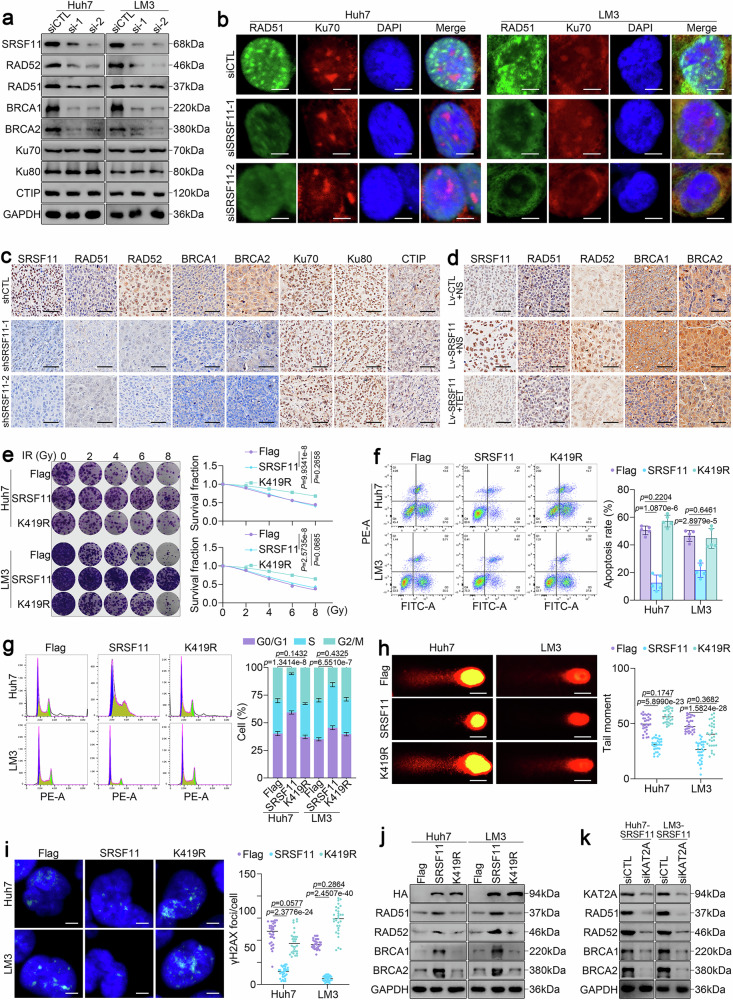
KAT2A-catalyzed K419 succinylation governs SRSF11-dependent HR activity. **a** Western blotting of the HR-related proteins SRSF11, RAD51, RAD52, BRCA1, and BRCA2, and the NHEJ-related proteins Ku70, Ku80, and CTIP in Huh7 and LM3 cells transfected with siSRSF11. **b** Immunofluorescence staining for the core protein of HR, RAD51 (green), and the core protein of NHEJ, Ku70 (red), in LM3 and Huh7 cells transfected with siSRSF11. Scale bars, 10 μm. **c** Representative images of IHC staining for the HR-related proteins RAD51, RAD52, BRCA1, and BRCA2 and the NHEJ-related proteins Ku70, Ku80, and CTIP in xenograft tumors infected with/without shSRSF11. Scale bars, 50 μm. **d** Representative images of IHC staining for the HR-related proteins RAD51, RAD52, BRCA1, and BRCA2 in xenograft tumors infected with Lv-SRSF11 and treated with/without TET. Scale bars, 50 μm. **e** Colony formation assays of LM3 and Huh7 cells transfected with Flag-CTL, Flag-SRSF11, or Flag-SRSF11 K419R, respectively, and treated with the indicated dose of IR. Flow cytometry analysis of apoptosis (**f**) and the cell cycle (**g**) in Huh7 and LM3 cells transfected with Flag-CTL, Flag-SRSF11, or Flag-SRSF11 K419R, respectively, and treated with 5 Gy of IR. **h** Comet assay of Huh7 and LM3 cells transfected with Flag-CTL, Flag-SRSF11, or Flag-SRSF11 K419R, respectively, and treated with 5 Gy of IR. Scale bars, 10 μm. **i** Immunofluorescence staining for γ-H2AX (green) and DAPI (blue) in LM3 and Huh7 cells transfected with Flag-CTL, Flag-SRSF11 or Flag-SRSF11 K419R, respectively, and treated with 5 Gy of IR. Scale bars, 10 μm. Western blotting assay of the expression of RAD51, BRCA1 and BRCA2 in Huh7 and LM3 cells transfected with Flag-CTL, Flag-SRSF11 or Flag-SRSF11 K419R, respectively, and treated with 5 Gy of IR (**j**), and the expression of RAD51, BRCA1 and BRCA2 in SRSF11-overexpressing Huh7 and LM3 cells transfected with siKAT2A (**k**). For all the above experiments, 3 independent experiments (*n* = 3) with similar results were performed in triplicate. For **e**‒**i**, the data are presented as the means ± SDs. The statistical analyses were performed via two-tailed unpaired Student’s *t*-tests. *p* < 0.05 was considered to indicate statistical significance

Based on these results, we hypothesized that SRSF11 regulates radioresistance by affecting DNA damage repair. HR and NHEJ are the two major pathways for DNA DSBs repair.^13^ To elucidate the potential mechanisms by which SRSF11 regulates DNA damage repair, we detected the expression of key genes involved in HR and NHEJ signaling. As a result, SRSF11 knockdown selectively downregulated HR mediators RAD51, RAD52, BRCA1, and BRCA2 without altering NHEJ components Ku70/80 or CTIP (Fig. 3a). Immunofluorescence confirmed the diminished RAD51 nuclear foci formation in SRSF11-deficient cells post-irradiation (Fig. 3b). Moreover, in vivo IHC assays also revealed that the expression of only HR-related proteins, but not NHEJ proteins, was reduced after SRSF11 silencing (Fig. 3c and Supplementary Fig. 3j). Conversely, SRSF11 overexpression significantly increased the expression of RAD51, RAD52, BRCA1, and BRCA2, and the expression of these factors was resuppressed upon TET addition (Fig. 3d and Supplementary Fig. 3k). Collectively, these data revealed that SRSF11 promotes DSBs repair in HCC by regulating HR but not NHEJ.

### KAT2A-catalyzed K419 succinylation governs SRSF11-dependent HR activity

We next investigated whether KAT2A-induced K419 succinylation of SRSF11 could modulate HR in HCC. We found that wild-type SRSF11, but not SRSF11 K419R, markedly enhanced DNA damage repair and radioresistance, as indicated by clonogenic assays (Fig. 3e), apoptosis (Fig. 3f), cell cycle (Fig. 3g), comet assay (Fig. 3h), and γH2AX foci (Fig. 3i) analyses. Moreover, compared with the control vector, the overexpression of wild-type SRSF11 resulted in a noticeable increase in the expression of HR-associated proteins, whereas SRSF11 K419R failed to exert this effect (Fig. 3j). Collectively, these data indicate that SRSF11 K419 is crucial for HR in HCC.

To further validate the HR-regulatory effects of K419 succinylation of SRSF11 catalyzed by KAT2A in HCC, we constructed SRSF11-knockout (SRSF11^KO^) Huh7 and LM3 cell lines. Through knocking down KAT2A expression after overexpressing SRSF11 wild-type or K419R, we found that KAT2A silencing caused suppression of cell proliferation (Supplementary Fig. 4a) and promotion of apoptosis (Supplementary Fig. 4b), G2/M phase arrest (Supplementary Fig. 4c), DNA damage (Supplementary Fig. 4d), and γH2AX foci formation (Supplementary Fig. 4e). These effects were abolished in K419R-reconstituted cells (Supplementary Fig. 4f–i). Conversely, KAT2A overexpression failed to cause noticeable alterations in radiotherapy sensitivity and DNA damage in SRSF11 K419R expressed SRSF11^KO^ cells (Supplementary Figure 4j-m). Critically, KAT2A knockdown selectively regulated HR-associated protein expression in wild-type (Fig. 3k) but not mutant SRSF11 contexts (Supplementary Fig. 4n, o). These data conclusively demonstrate that KAT2A-mediated K419 succinylation licenses SRSF11’s capacity to orchestrate HR-dependent genomic stabilization in HCC.

### SRSF11 governs RAD52 pre-mRNA splicing to modulate homologous recombination

**Fig. 4:**
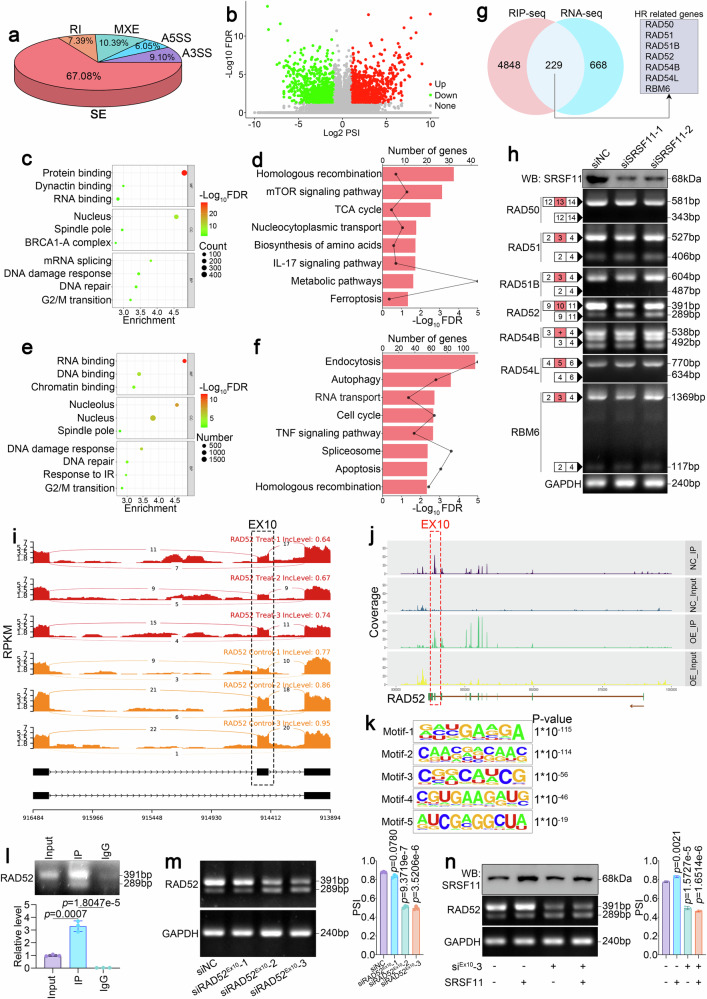
SRSF11 promotes the inclusion of RAD52 exon 10. **a** Pie chart depicting the proportions of different types of AS events based on the RNA-seq data from Huh7 cells after the knockdown of SRSF11. SE, skipped exons, RI, retained introns, A5SS, alternative 5′ splice site, A3SS, alternative 3′ splice site, MXE, mutually exclusive exons. **b** Volcano plot showing the different alternative splicing of SE. **c** GO analysis of different alternative splicing genes. **d** KEGG analysis of different alternative splicing genes. **e** GO analysis of SRSF11-binding genes. **f** KEGG analysis of SRSF11-binding genes. **g** Venn diagram of SRSF11-binding genes from RIP-seq and different splicing genes from RNA-seq. **h** RT‒PCR was performed to validate the AS events of seven intersecting HR-related genes. **i** Visualization of exon 10 skipping of RAD52 by the Sashimi plots treated by knockdown of SRSF11 with siRNAs. **j** Coverage plots of peak reads in the RAD52 visualized with IGV using RIP-seq data. **k** Enriched sequence elements of the top 5 SRSF11-binding motifs. **l** RIP and RT‒PCR detection of RAD52 pre-mRNA binding to SRSF11 by anti-SRSF11 antibody. **m** RT‒PCR analysis of siRNAs specifically targeting exon 10 (siRAD52^Ex10^). **n** RT‒PCR detection of exon 10 skipping of RAD52 via the overexpression of SRSF11 or combined knockdown of RAD52 with siRAD52^Ex10^. For **l**‒**n**, the data are presented as the means ± SDs. For **h** and **l**‒**n**, 3 independent experiments (*n* = 3) with similar results were performed in triplicate. The statistical analyses were performed via two-tailed unpaired Student’s *t*-tests. *p* < 0.05 was considered to indicate statistical significance

Although we confirmed that K419 succinylation promotes SRSF11-mediated HR, the intrinsic mechanism by which these proteins regulate HR remains obscure. SRSF family proteins play crucial roles in alternative splicing (AS).^28^ Therefore, we performed RNA-seq to identify global SRSF-mediated alternative splicing events by SRSF11 silencing in HCC cells. A total of 89600 alternative splicing events of five types were identified, and exon skipping (SE) accounted for 67.08% of all splicing events (Fig. 4a), indicating that SE is the main type of splicing event regulated by SRSF11. Among these splicing events, 897 different (FDR < 0.05, |ΔPSI | > 0.1) splicing events were identified (Fig. 4b). Gene Ontology (GO) analysis of these different splicing genes showed that the DNA damage response and DNA repair were markedly enriched (Fig. 4c). KEGG analysis showed that HR was the most highly enriched signaling pathway (Fig. 4d), which is highly consistent with our above results that SRSF11 regulates HR.

To further investigate the direct targets of SRSF11-mediated HR, we performed RNA immunoprecipitation sequencing (RIP-seq) to identify SRSF11-binding mRNAs. A total of 5077 genes that bind to SRSF11 were identified. GO and KEGG analysis of SRSF11-binding genes further confirmed that DNA damage repair and HR signaling were remarkably enriched (Fig. 4e, f). Based on the integrative screening of these SRSF11-binding genes and different splicing events, we identified seven HR-associated candidates, namely, RAD50, RAD51, RAD51B, RAD52, RAD54B, RAD54L and RBM6^29^ (Fig. 4g). Next, we designed primers near skipped exons to further validate the splicing of these genes via RT‒PCR and confirmed that only the RAD51, RAD52, RAD54B and RBM6 underwent AS (Fig. 4h and Supplementary Fig. 5a). Among them, only the exon 10 skipping of RAD52 showed noticeable increases after SRSF11 silencing (Fig. 4h and Supplementary Fig. 5a). Furthermore, the Sashimi plot from the AS analysis illustrated that the exon 10 skipping event of RAD52 was increased after SRSF11 silencing, and the coverage plots showed that the coverage surrounding exon 10 was notably increased in SRSF11-IP group, according to the RNA-seq and RIP-seq data (Fig. 4I, j). Previous study has reported that the CUGAGU 6-mer motif is the preference sequences of SRSF11.^30^ Consistently, we used the HOMER algorithm to identify the SRSF11-recognizing RNA motif of RAD52 and found that the motif containing CUGAGU was remarkably enriched (Fig. 4k), which is near the intron‒exon junction. Moreover, RIP assay further validated the direct binding of SRSF11 to RAD52 (Fig. 4l). To further confirm that RAD52 is a direct downstream functional target of SRSF11, we designed siRNAs specifically targeting exon 10 (siRAD52^Ex10^) of RAD52 (Fig. 4m). We demonstrated that SRSF11 silencing increased the exon 10 skipping of RAD52. Here, we further confirmed that SRSF11 overexpression reduced the exon 10 skipping of RAD52 (Fig. 4n). However, after knocking down the expression of RAD52 by siRAD52^Ex10^, the overexpression of SRSF11 did not markedly increase the exon 10 inclusion (Fig. 4n). These data further support that exon 10 of RAD52 is a direct splicing sequence of SRSF11.

To determine whether the RAD52 exon 10 skipping occurs in HCC patients, we detected its expression in HCC and NCL tissues using RT-PCR. Surprisingly, the exon 10 skipping of RAD52 in HCC tissues consistently decreased compared to NCL tissues in detected samples (Supplementary Fig. 5b). To further investigate the influence of the exon 10 inclusion of RAD52 in HCC tissues on the overall survival (OS) and relapse-free survival (RFS) in our clinical cohort, we designed the probes specifically targeting exon 10 of RAD52 for RNA In Situ Hybridization to detect the quantity of RAD52 with or without exon 10 using paraffin sections (Supplementary Fig. 5c). As expected, high expression of RAD52 with exon 10 was remarkably associated with poor OS and RFS in HCC patients (Supplementary Fig. 5d, e).

### SRSF11-dependent RAD52 exon 10 inclusion facilitates RAD51 complex assembly

**Fig. 5:**
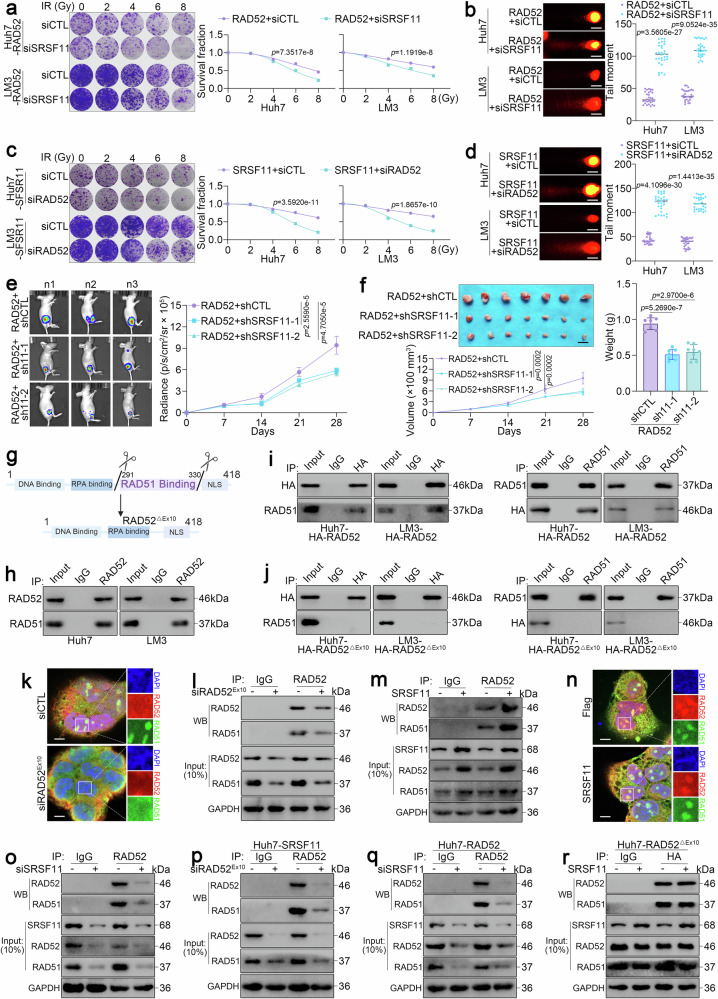
SRSF11 promotes RAD52/RAD51 dimer formation by regulating RAD52 splicing. **a** Colony formation assays of RAD52-overexpressing LM3 and Huh7 cells transfected with siSRSF11 and treated with the indicated dose of IR. **b** Comet assay of RAD52-overexpressing LM3 and Huh7 cells transfected with siSRSF11 and treated with 5 Gy of IR. Scale bars, 10 μm. **c** Colony formation assays of SRSF11-overexpressing LM3 and Huh7 cells transfected with siRAD52^Ex10^ and treated with the indicated dose of IR. **d** Comet assay of SRSF11-overexpressing LM3 and Huh7 cells transfected with siRAD52^Ex10^ and treated with 5 Gy of IR. Scale bars, 10 μm. Luciferase signals (**e**) and representative images of the xenograft, tumor volume, and tumor weight (**f**) of xenograft model in nude mice using RAD52-overexpressed LM3 cells infected with shSRSF11 and treated with 5 Gy of IR. (*n* = 7 per group). Scale bars, 1 cm. **g** The schematic diagram showing that exon 10 skipping of RAD52 led to the deletion of the RAD51 binding region. **h** Western blotting assay of the binding of RAD51 with RAD52 after immunoprecipitation with an anti-RAD52 antibody. **i** Western blotting assay of the binding of RAD51 with RAD52 by overexpressing HA-RAD52 after immunoprecipitation with an anti-HA antibody (left) or anti-RAD51 antibody (right). **j** Western blotting assay of the binding of RAD51 with RAD52 in HA-RAD52^△Ex10^-overexpressing cells after immunoprecipitation with an anti-HA antibody (left) or anti-RAD51 antibody (right). **k** Immunofluorescence staining for RAD51 (green) and RAD52 (red) and DAPI (blue) in Huh7 cells transfected with or without siRAD52^Ex10^. Scale bars, 5 μm. **l** Western blotting assay of the binding of RAD51 with RAD52 after immunoprecipitation with an anti-RAD52 antibody in Huh7 cells transfected with or without siRAD52^Ex10^. **m** Western blotting assay of the binding of RAD51 with RAD52 after immunoprecipitation with an anti-RAD52 antibody in Huh7 cells transfected with or without Flag-SRSF11. **n** Immunofluorescence staining for RAD51 (green) and RAD52 (red) and DAPI (blue) in Huh7 cells transfected with or without Flag-SRSF11. Scale bars, 5 μm. **o** Western blotting assay of the binding of RAD51 with RAD52 after immunoprecipitation with an anti-RAD52 antibody in Huh7 cells transfected with or without siSRSF11. **p** Western blotting assay of the binding of RAD51 with RAD52 after immunoprecipitation with an anti-RAD52 antibody in SRSF11-overexpressing Huh7 cells transfected with or without siRAD52^Ex10^. **q** Western blotting assay of the binding of RAD51 with RAD52 after immunoprecipitation with an anti-RAD52 antibody in RAD52-overexpressing Huh7 cells transfected with or without siSRSF11. **r** Western blotting assay of the binding of RAD51 with RAD52 after immunoprecipitation with an anti-HA antibody in RAD52^ΔEx10^-overexpressing Huh7 cells transfected with or without Flag-SRSF11. For **a**‒**d** and **i**‒**r**, 3 independent experiments (*n* = 3) with similar results were performed in triplicate. For **a**‒**f**, the data are presented as the means ± SDs. The statistical analyses were performed via two-tailed unpaired Student’s *t*-tests. p < 0.05 was considered to indicate statistical significance

Next, we sought to investigate whether exon 10 skipping of RAD52 mediated by SRSF11 silencing participates in regulating HR. First, we found that SRSF11 silencing dramatically augmented DNA damage caused by IR in RAD52-overexpressing HCC cells, as shown by a colony formation assay (Fig. 5a), comet assay (Fig. 5b), γH2AX staining (Supplementary Fig. 6a), and limited HR (Supplementary Fig. 6b). Moreover, siRAD52^Ex10^ also markedly increased DNA damage (Fig. 5c, d and Supplementary Fig. 6c) and limited HR (Supplementary Fig. 6d) induced by IR in SRSF11-overexpressing HCC cells. Consistent with these results, in the nude mouse xenograft model, we overexpressed HA-RAD52 and knocked down SRSF11 and found that knocking down SRSF11 significantly inhibited tumor growth in vivo compared with that in the control group (Fig. 5e, f). These results indicate that exon 10 skipping of RAD52, regulated by SRSF11 could impair HR. Then, to further confirm the role of exon 10 skipping of RAD52 in impairing HR, we constructed an HA-fused RAD52 truncation mutation plasmid lacking exon 10 (HA-RAD52^△Ex10^). Unsurprisingly, compared with the control, HA-RAD52^△Ex10^ overexpression alone did not appreciably alter IR-induced DNA damage in SRSF11-overexpressing RAD52^KO^ HCC cells (Supplementary Fig. 6e–g). After knocking down of SRSF11, overexpression of RAD52 alone can promote DNA damage repair induced by IR, but overexpression of HA-RAD52^△Ex10^ alone cannot promote DNA damage repair (Supplementary Fig. 6h, i). These results indicate that exon 10 skipping of RAD52 inhibits HR-mediated DNA damage repair in HCC.

Notably, the protein encoded by RAD52 exon 10 (289–322 amino acids) contains the RAD51 binding domain (291–330 amino acids) that promotes HR (Fig. 5g).^15,31^ As we all known, RAD51 is a central recombinase essential for HR polymerase-mediated template-driven synthesis. Thus, we deduced that exon 10 skipping of RAD52 affects mainly the interaction between RAD52 with RAD51 in HR. To verify the above hypothesis, we investigated the direct effect of exon 10 skipping of RAD52 on the interaction of RAD51. We first confirmed the endogenous reciprocal binding of RAD52 to RAD51 (Fig. 5h). Then, by overexpressing HA-RAD52 in Huh7 and LM3 cells, the binding of RAD52 and RAD51 was further confirmed through Co-IP assay (Fig. 5i), whereas no significant RAD51 binding to RAD52 was detected after overexpressing HA-RAD52^△Ex10^ (Fig. 5j). Immunofluorescence assays further revealed the colocalization of RAD52 and RAD51 in HCC cells (Fig. 5k). Notably, after knockdown of exon 10 of the RAD52 transcript using siRAD52^Ex10^ specifically, the binding of RAD51 with RAD52 was significantly suppressed (Fig. 5k, l and Supplementary Fig. 6j). These results showed that exon 10 of RAD52 is critical for RAD52 binding to RAD51. To further explore the effect of SRSF11-mediated exon 10 inclusion of RAD52 on RAD51 binding, we found that overexpression of SRSF11 promoted the binding between RAD52 and RAD51 using Co-IP (Fig. 5m and Supplementary Fig. 6k) and immunofluorescence assays (Fig. 5n), In contrary, knocking down SRSF11 suppressed the binding of RAD52 and RAD51 (Fig. 5o and Supplementary Fig. 6l). Consistently, siRAD52^Ex10^ resulted in reduced binding of RAD52 and RAD51 in SRSF11-overexpressing cells (Fig. 5p and Supplementary Fig. 6m). siSRSF11 also had similar effects on RAD52-overexpressing cells (Fig. 5q and Supplementary Fig. 6n). However, the overexpression of SRSF11 had no significant effect on the binding of RAD52 and RAD51 in HA-RAD52^△Ex10^ cells (Fig. 5r). These data establish that SRSF11-mediated RAD52 exon 10 inclusion licenses RAD51 binding, thereby enabling HR-dependent genomic stabilization in HCC.

### KAT2A-catalyzed succinylation licenses SRSF11’s spliceosome recruitment capacity

**Fig. 6:**
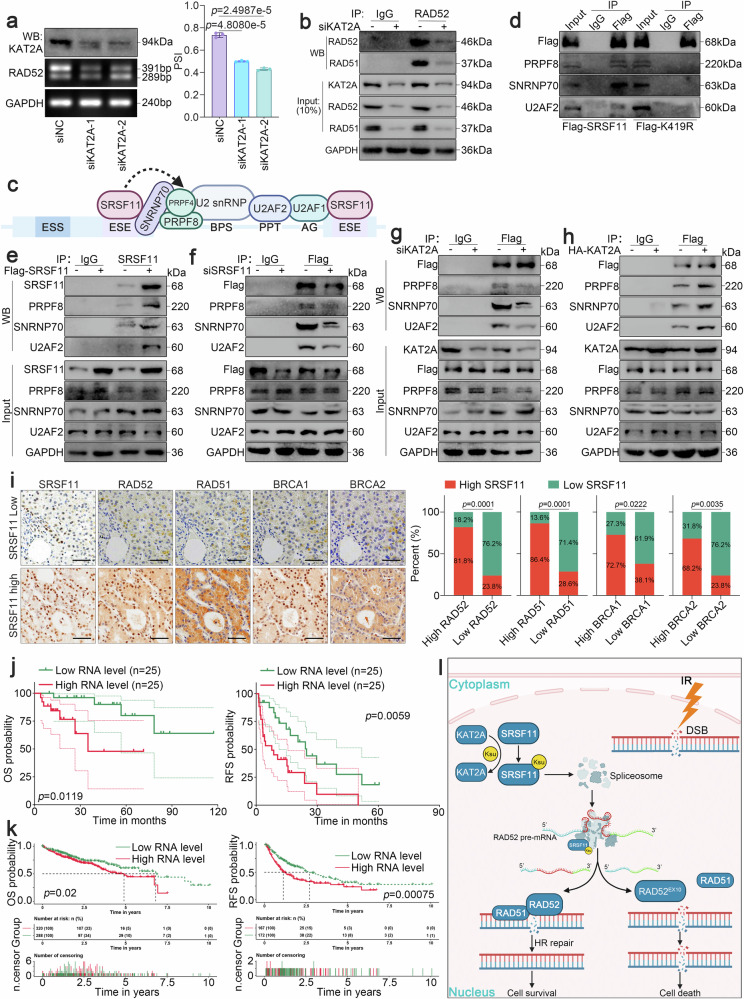
KAT2A-catalyzed succinylation licenses SRSF11’s spliceosome recruitment capacity and SRSF11 expression is correlated with adverse clinical outcomes in HCC. **a** RT‒PCR detection of exon 10 skipping of RAD52 in Huh7 cells transfected with siKAT2A. **b** Western blotting assay of the binding of RAD51 with RAD52 after immunoprecipitation with an anti-RAD52 antibody in Huh7 cells transfected with or without siKAT2A. **c** Schematic diagram showing SRSF11-associated spliceosome elements. **d** Western blotting assay of the binding of U2AF2, PRPF8, and SNRNP70 with SRSF11 after immunoprecipitation with an anti-Flag antibody in Huh7 cells transfected with Flag-SRSF11 or Flag-SRSF11 K419R, respectively. **e** Western blotting assay of the binding of U2AF2, PRPF8, and SNRNP70 with SRSF11 after immunoprecipitation with an anti-Flag antibody in Huh7 cells transfected with Flag-SRSF11. **f** Western blotting assay of the binding of U2AF2, PRPF8, and SNRNP70 with SRSF11 after immunoprecipitation with an anti-Flag antibody in Huh7 cells transfected with siSRSF11. **g** Western blotting assay of the binding of U2AF2, PRPF8, and SNRNP70 with SRSF11 after immunoprecipitation with an anti-Flag antibody in Huh7 cells transfected with siKAT2A. **h** Western blotting assay of the binding of U2AF2, PRPF8, and SNRNP70 with SRSF11 after immunoprecipitation with an anti-Flag antibody in Huh7 cells transfected with HA-KAT2A. **i** Representative images of IHC staining of SRSF11, RAD51, RAD52, BRCA1 and BRCA2 in HCC samples with high or low expression of SRSF11 (left) and correlation analysis (right). **j** OS and RFS curves for SRSF11 high expression and low expression group from our clinical cohort. The area around each dashed line represents a 95% confidence interval. **k** OS and RFS curves using transcriptomic from TCGA-LIHC database. **l** Diagram illustrating that KAT2A directly promotes succinylation at K419 site of SRSF11, followed by the formation of spliceosomes and enhanced inclusion of exon 10 of RAD52, thereby enhancing interaction with RAD51 and ultimately promoting HR of DSBs and radioresistance in tumor cells. Exon 10 skipping of RAD52 can reduce the recruitment of RAD51 and impair HR, thereby promoting radiation-induced tumor cell death. For **a**‒**b**, **d** and **e**‒**h**, 3 independent experiments (*n* = 3) with similar results were performed in triplicate. For a, the data are presented as the means ± SDs. The statistical analyses were performed via two-tailed unpaired Student’s *t*-tests. *p* < 0.05 was considered to indicate statistical significance

We next investigated the role of KAT2A-mediated K419 succinylation of SRSF11 in RAD52 splicing. We first confirmed that KAT2A silencing dramatically increased the exon 10 skipping of RAD52 pre-mRNAs (Fig. 6a). The overexpression of SRSF11 decreased the exon 10 skipping of RAD52, but the expression of SRSF11 K419R did not significantly change in KAT2A-knocked down Huh7 cells (Supplementary Fig. 7a). Meanwhile, knockdown of KAT2A suppressed the binding of RAD52 and RAD51 (Fig. 6b and Supplementary Fig. 7b). Moreover, after knocking down KAT2A, we found that overexpression of Flag-SRSF11 or Flag-SRSF11 K419R did not noticeably affect the binding of RAD52 to RAD51 in SRSF11^KO^ cells (Supplementary Fig. 7c). These results demonstrated that KAT2A mediated K419 succinylation regulates the binding of RAD52 with RAD51 by SRSF11-mediated exon 10 skipping of RAD52.

Next, we further investigate the potential mechanism by which KAT2A-mediated K419 succinylation regulates SRSF11-mediated RAD52 splicing. As shown in Fig. 1, KAT2A-mediated K419 succinylation did not affect the expression of SRSF11, and the K419 residue is adjacent to the RRM domain and RS domain. Thus, we deduce that K419 succinylation may affect the binding of SRSF11 with RAD52 pre-mRNAs and the key splicing proteins. To this end, we first investigated whether KAT2A-mediated K419 succinylation affects the binding of SRSF11 to RAD52 pre-mRNAs. We overexpressed Flag-SRSF11 or Flag-SRSF11 K419R in HCC cells, respectively, and found that the RAD52 pre-mRNAs binding to SRSF11 was dramatically suppressed in overexpressed Flag-SRSF11 K419R cells (Supplementary Fig. 7d). Next, we explored whether KAT2A-mediated K419 succinylation affects SRSF11-associated spliceosome formation. We analyzed the correlations of several key proteins of the spliceosome [including U2 small nuclear RNA auxiliary factor 1 (U2AF1), U2 small nuclear RNA auxiliary factor 2 (U2AF2), pre-mRNA processing splicing factor 4 (PRPF4), pre-mRNA processing splicing factor 8 (PRPF8), SNRNP70, small nuclear ribonucleoprotein polypeptides B and B1 (SNRPB), DEAH (Asp-Glu-Ala-His) box polypeptide 15 (DHX15), BRR2 and SF3B1] with KAT2A and SRSF11 using the TCGA database (Fig. 6c). The data from the TCGA database indicated that the expression of U2AF1, U2AF2, PRPF4, PRPF8, SNRNP70, SNRPB, DHX15, SF3B1 and BRR2 was positively correlated with the expression of SRSF11 at both the mRNA and protein levels (Supplementary Fig. 7e, f). Moreover, the expression of U2AF1, U2AF2, PRPF4, PRPF8, SNRNP70, SNRPB, DHX15, SF3B1 and BRR2 was also positively correlated with the expression of KAT2A (Supplementary Fig. 7g). In addition, the expression of these genes was positively correlated with the expression of RAD51 and RAD52 (Supplementary Fig. 8a, b). Thus, we deduced that KAT2A-mediated K419 succinylation promotes SRSF11-associated spliceosome formation. To verify the above hypothesis, we confirmed the binding of SRSF11 to the U2AF2/PRPF8/SNRNP70 complex via Co-IP assay (Fig. 6d). Conversely, we found that overexpressing SRSF11 promoted the binding of SRSF11 to the U2AF2/PRPF8/SNRNP70 complex (Fig. 6e), whereas knocking down SRSF11 suppressed the binding of SRSF11 to U2AF2/PRPF8/SNRNP70 complex (Fig. 6f). Importantly, knocking down KAT2A suppressed the binding of SRSF11 to these proteins (Fig. 6g), whereas overexpression of KAT2A induced in the opposite effects (Fig. 6h) in SRSF11 overexpressed HCC cells. These data delineate a KAT2A-SRSF11 axis in which K419 succinylation licenses spliceosome recruitment to enforce RAD52 exon 10 inclusion, thereby enabling HR proficiency.

### SRSF11 expression is correlated with HR proficiency, radiorestistance, and adverse clinical outcomes in HCC

Here, we further analyzed the correlations of SRSF11 expression with HR-mediated DSBs repair, radiotherapy sensitivity and survival outcomes in HCC patients. We first confirmed that, compared with that in THLE2 cells, SRSF11 expression was significantly higher in HCC cell lines (Supplementary Fig. 9a). We then established diethyl nitrosamine (DEN)- and β-catenin^ΔN90^-induced orthotopic mouse HCC models, and the IHC results indicated that SRSF11 expression was higher in HCC tissues than in NCL tissues in both models (Supplementary Fig. 9b). Consistently, in our clinical cohort, SRSF11 was more highly expressed in HCC tissues than in NCL tissues (Supplementary Fig. 9c). We further validated the above results using public databases. Indeed, the transcriptomic and proteomic data from the TCGA-LIHC cohort further verified that SRSF11 mRNA and protein levels were markedly higher in HCC tissues than in NCL tissues (Supplementary Fig. 9d, e). Finally, using single-cell transcriptome data (GSE166635), we further verified that SRSF11 expression was indeed higher in Malignant cells than in Non-Malignant cells (Supplementary Fig. 9f).

We next evaluated the impact of SRSF11 on the sensitivity of HCC patients to HR and radiotherapy. A total of 50 HCC patients were included in our study; the HCC samples were classified into high expression (IHC score>5, *N* = 24) and low expression (IHC score<5, *N* = 26) groups based on IHC scores. Chi-square analysis indicated that SRSF11 levels were markedly positively correlated with RAD52 (*P* = 0.0001), RAD51 (*P* = 0.0001), BRCA1 (*P* = 0.0222) and BRCA2 (*P* = 0.0035) levels (Fig. 6i). Consistent with our cohort, SRSF11 levels were remarkedly positively correlated with RAD52 (*P* = 3.44 × 10^−58^), RAD51 (*P* = 8.69 × 10^−12^), BRCA1 (*P* = 1.08 × 10^−22^), and BRCA2 (*P* = 1.90 × 10^−29^) levels (Supplementary Fig. 10a) from TCGA-LIHC. We further adapted the Radiosensitivity Index, DNA Damage Repair Score, and HR Score to evaluate the impacts of SRSF11 on radiosensitivity, DNA damage repair, and HR in patients from TCGA-LIHC and GSE166635. Both transcriptomic and proteomic data suggest that high SRSF11 expression is associated with HR-mediated repair and radioresistance in HCC (Supplementary Fig. 10b–d). Collectively, these data strongly support the notion that SRSF11 confers HR and radioresistance.

The strong association between SRSF11 expression and radioresistance prompted us to examine whether high SRSF11 expression is correlated with poor clinical outcomes in HCC patients. Indeed, our cohort indicated that high SRSF11 expression was remarkably associated with poor OS and RFS in HCC patients (Fig. 6j). According to transcriptomic and proteomic data from the TCGA-LIHC cohort, high SRSF11 expression was indeed significantly associated with short OS and RFS in patients with HCC (Fig. 6k and Supplementary Fig. 11a). Together, these results revealed that high expression of SRSF11 is correlated with radioresistance and poor survival in patients.

## Discussion

Mounting evidence underscores the critical role of succinylation in the initiation and progression of tumors, yet its functional architecture in HCC remains poorly mapped. Here, we systematically identified SRSF11 as an essential functional target of succinylation and demonstrated that KAT2A-elicited K419 succinylation of SRSF11 promotes the HR of DSBs by regulating SRSF11-mediated exon 10 skipping of RAD52, thereby promoting DNA damage repair and radioresistance. Inhibition of the KAT2A-SRSF11 axis can promote RAD52 exon 10 skipping, thereby suppressing the interaction of RAD52 with RAD51, impairing HR and promoting radiation-induced tumor cell death across preclinical models (Fig. 6l), positioning this pathway as a therapeutic vulnerability.

Although great efforts have been made to explore the pathological mechanisms of HCC,^32^ current therapies for advanced HCC yield suboptimal outcomes, indicating that new mechanisms underlying HCC progression and treatment resistance need to be further investigated to identify novel potential therapeutic targets. Succinylation exerts multifaceted control over oncogenic processes through dynamic lysine modification that structurally and functionally reprogram target proteins, orchestrating tumor progression via metabolic adaptation, transcriptional rewiring, signal transduction cascades, and immune microenvironment remodeling.^22,33^ Despite these pan-cancer implications, the HCC succinylome remains largely uncharted territory, with functional characterization limited to a fraction of modified substrates. The current understanding of the mechanistic contributions of succinylation to HCC—particularly its intersection with DNA repair fidelity and therapeutic resistance—represents a critical knowledge gap. Systematic dissection of context-dependent succinyltransferase networks and their disease-relevant targets is imperative to unlock the translational potential of this regulatory axis in HCC management.

The succinylome is principally fueled by tricarboxylic acid (TCA) cycle-derived succinyl-CoA, with its spatiotemporal dynamics governed through balanced enzymatic control via succinyltransferases and desuccinylases.^34^ Here, we observed the global succinylation hyperactivation in HCC, which is consistent with previous reports.^35,36^ Given these findings, we employed LC-MS/MS proteomic profiling to systematically identify SRSF11 as a conserved succinylation substrate across HCC cell lines, followed by validation in patient-derived tissues. Mechanistically, domain-specific mutagenesis (K419R) and custom-generated anti-succinyl lysine antibodies against SRSF11, and gain/loss-of-function studies of candidate enzymes converged to establish KAT2A as the dominant regulator of SRSF11 succinylation. This modification was proved to be enzymatically recapitulable in vitro through succinyl-CoA supplementation, confirming the catalytic competency of KAT2A. While KAT2A has been implicated in tumorigenic processes,^34,37^ our work provides direct evidence of its HCC-specific role: KAT2A ablation suppressed malignant progression in preclinical models predominantly mediated through SRSF11 succinylation attenuation and established a causal hierarchy wherein KAT2A-mediated succinylation licenses the oncogenic functionality of SRSF11.

While SRSF family proteins are established orchestrators of oncogenic splicing programs in tumor initiation, metastasis, and therapeutic resistance,^38,39^ the mechanistic underpinnings of SRSF11—particularly its intersection with the DDR—have remained enigmatic. Building on our prior discovery that SRSF11 sustains HCC proliferation and drug resistance,^27^ transcriptomic profiling of SRSF11-depleted cells revealed its critical involvement in HR-mediated DDR. This finding assumes clinical significance given that radiation-induced DNA damage, while cytotoxic to malignancies, is counteracted by tumor cells through compensatory upregulation of the repair machinery—a key mediator of radioresistance.^10,12,40^ HR and NHEJ are the main repair pathways of DNA damage repair.^13^ Strikingly, IR-challenged HCC models demonstrated that SRSF11 ablation selectively impaired HR pathway activation while sparing NHEJ, establishing HR as the dominant DDR effector of SRSF11 in HCC. Mechanistically, we resolved that KAT2A-catalyzed succinylation at SRSF11-K419 serves as the molecular switch governing this process. Both K419R mutagenesis and KAT2A silencing attenuated SRSF11 succinylation, concomitantly suppressing HR proficiency and HCC growth—effects phenocopied by RAD52 exon 10 exclusion. This splicing event, driven by succinylation-dependent spliceosome recruitment (U1/U2 snRNP and tri-snRNP complexes), preserves RAD52’s RAD51-binding domain, which is essential for strand invasion.^28,38^ Our findings contrast with reported FEN1 succinylation mechanisms involving Rad9-Rad1-Hus1 complex engagement,^41^ highlighting the PTM-specific regulation of DDR subpathways. Crucially, succinylation enhanced the dual functionality of SRSF11: (1) direct pre-mRNAs binding of RAD52, and (2) physical scaffolding of spliceosomal proteins—a coordinated mechanism ensuring RAD52 isoform fidelity.

AS serves as a pivotal RNA processing mechanism that expands proteomic diversity through regulated exon inclusion/exclusion, with its dysregulation emerging as a hallmark of oncogenic transformation and therapeutic resistance.^38,42,43^ AS aberrations have been mechanistically linked to DDR subversion—a phenomenon exemplified by SRSF3-mediated ANRIL splicing in HR potentiation of pancreatic cancer.^44^ Our findings extend this paradigm by demonstrating that SRSF11 governs RAD52 pre-mRNA splicing fidelity, wherein KAT2A-dependent succinylation prevents exon 10 exclusion to preserve the RAD51-binding domain essential for HR proficiency.

Structural dissection reveals the multifunctionality of human RAD52: two DNA-binding domains orchestrate strand annealing, while discrete regions mediate RPA interaction, RAD51 binding, and nuclear compartmentalization.^15,45^ Crucially, exon 10-encoded sequences constitute the RAD51 engagement interface—a feature rationalizing our observation that SRSF11-driven exon skipping ablates RAD52-RAD51 complex formation. As the central recombinase required for DSBs repair,^46^ RAD51 is progressively loaded onto the ssDNA end with the involvement of BRCA1 and BRCA2 to form nuclear protein filaments on ssDNA, promoting strand invasion and facilitating homologous pairing reactions. Unlike yeast RAD52, although human RAD52 is considered to lack mediator activity, IR-induced RAD52-RAD51 lesions can readily form in the presence or absence of BRCA2.^15,16^ Accumulating evidence has shown the crucial role and complex mechanisms of human RAD52 with RAD51 in HR through BRCA2-independent mechanisms, including RAD51 recruitment to active DSBs sites, filament stabilization, and multiple invasions.^16,47–50^ The functions of RAD52 were recapitulated in our IR-induced models, underscoring the indispensable role of RAD52 in RAD51-dependent HR of HCC. Experimental validation via exon 10-specific truncation mutants of RAD52 confirmed the domain’s necessity for RAD51 interaction, phenocopying the effects of SRSF11 knockdown. These results crystallize a mechanism whereby SRSF11 succinylation enforces RAD52 isoform integrity. The exquisite sensitivity of HR to RAD52 splicing perturbations highlights the therapeutic potential of targeting succinylation-dependent AS nodes to disrupt DDR adaptation in radioresistant HCC. Furthermore, perturbation of the KAT2A–SRSF11 axis led to decreased protein levels of key HR effectors. A similar reduction was observed following RAD52 disruption, suggesting the activation of compensatory ubiquitin–proteasome-mediated degradation. These findings indicate a dual-layer regulatory mechanism underpinning HR impairment, which merits further investigation.

Collectively, our findings establish succinylation as a master regulatory switch governing homologous recombination fidelity in hepatocellular carcinoma through PTM-directed control of RAD52 splicing. By elucidating the KAT2A-SRSF11 axis as the enzymatic-structural nexus coupling lysine succinylation to spliceosome remodeling, we demonstrate how SRSF11-K419 modification enforces RAD52 exon 10 inclusion—a critical determinant of RAD51 filament assembly and strand invasion efficiency. This mechanistic understanding provides a rational basis for targeting succinylation-dependent splice-switching to overcome radioresistance.

While this work delineates a novel succinylation-splicing-DNA repair axis, several frontiers require further exploration: the potential interplay between SRSF11-mediated splicing dysregulation and tumor microenvironment dynamics remains uncharacterized and merits investigation through single-cell transcriptomics or patient-derived organoid models; the broader landscape of SRSF11-regulated splicing events beyond RAD52 and their contributions to HCC warrants systematic profiling; and the molecular machinery linking SRSF11 ablation to accelerated RAD51/BRCA1/2 degradation—potentially involving ubiquitin-proteasome activation—demands mechanistic dissection to identify its potential as the multi-pathway target.

Notwithstanding these open questions, the tumor cell death elicited by KAT2A/SRSF11 inhibition—selectively disabling HR without perturbing NHEJ—positions this pathway as a precision therapeutic vulnerability in radiation oncology. Our work opens new avenues for co-targeting succinyl-CoA flux and DNA damage response pathways to potentiate radiotherapy in advanced HCC.

## Materials and methods

The animal experiments were performed in accordance with the National Institutes of Health’s Guide for the Care and Use of Laboratory Animals. All operations were approved by the Animal Care and Use Committee of West China Hospital of Sichuan University (2020351 A). The HCC samples and clinical characteristics collected from patients were approved by the Ethics Committee on Biomedical Research, West China Hospital of Sichuan University (2016, no. 120) and were conducted in accordance with the Declaration of Helsinki. Written informed consent was obtained from each patient.

### Clinical specimens

We collected 6 pairs of fresh-frozen HCC tissues and NCL tissues for SRSF11 lysine succinylation analysis and 50 paraffin-embedded HCC samples for correlation and survival analysis; these samples were obtained from patients treated at West China Hospital of Sichuan University between June 2013 and December 2018. Tumor pathological types were classified according to the WHO classification and confirmed by a clinicopathological diagnosis of HCC. The tumor-node-metastasis (TNM) stage was reclassified on the basis of the 7th edition of Cancer Staging Manual of the American Joint Committee on Cancer (AJCC). OS was defined as the interval between the first operation date and the date of death from any cause or the date of the last follow-up visit. RFS was defined as the date from the first operation to tumor recurrence or death. The clinical characteristics of the patients are listed in Supplementary Table 2.

### Cell culture

Hep3B, Huh7, PLC5, LM3 and 293 T cells were obtained from the Cell Bank of the Chinese Academy of Sciences (Shanghai, China) and were cultured in high-glucose DMEM (Gibco). The THLE2 cell line was obtained from the Meisen Chinese Tissue Culture Collection (MeisenCTCC, Zhejiang, China) and cultured in BEGM (MeisenCTCC). All media were supplemented with 10% fetal bovine serum (ScienCell, CA, USA) and 1% penicillin and streptomycin. All the cell lines were cultured in a constant-temperature incubator with 5% CO_2_ at 37 °C. All the cell lines used in the experiments were authenticated by short tandem repeat profiling and were assessed for mycoplasma contamination with the Cycleave polymerase chain reaction (PCR) mycoplasma detection kit (Takara).

### LC‒MS/MS analysis

To obtain lysine succinylation sites, an anti-pan-Ksu antibody (PTMbio, PTM-419, 1:500) was used to immunoprecipitate succinylated proteins. The immunoprecipitates from Huh7, LM3, PLC5, and Hep3B cells were subsequently separated via SDS‒PAGE. The collected proteins were subjected to LC‒MS/MS detection using an UltiMate 3000 RSLCnano nanoliter liquid chromatography tandem Q Exactive HF mass spectrometer (Thermo Fisher). The raw data were analyzed using MaxQuant (version 1.6.6.0) software for database searches and peptide spectrum matching.

### RNA-seq

Huh7 and LM3 cells with and without SRSF11 knockdown were subjected to RNA-seq. Total RNA was extracted from these cells with a TRIzol reagent kit. Next, quality control of the extracted RNA was performed using NanoDrop ND-1000 (NanoDrop, Wilmington, DE, USA) and Bioanalyzer 2100 (Agilent, CA, USA). The captured mRNA was subsequently fragmented using the NEBNextR Magnesium RNA Fragmentation Module (cat. E6150S, USA). The fragmented RNA was used for library construction. The constructed library was subjected to 2 × 150 bp paired-end sequencing (PE150) using Illumina NovaSeqTM 6000 (LC Bio Technology Co., Ltd. Hangzhou, China) according to standard procedures. Cutadapt (version: cutadapt-1.9) was used to obtain high-quality clean reads, and the reads of all the samples were subsequently aligned to the human reference genome using HISAT2. The analysis of gene differential expression, Gene Ontology (GO), Pathway Enrichment Analysis (KEGG), and Gene Set Enrichment Analysis (GSEA) was performed by DESeq2 software, Gene Ontology database, KEGG database and GSEA (v4.1.0) and MSigDB software, respectively.

### RNA immunoprecipitation (RIP)

RNAs that bind to SRSF11 were identified with the RIP kit (BersinBio, Guangzhou, China). In brief, polysome lysis buffer mixed with protease inhibitors and RNase inhibitors was used to lyse the cells on ice. After the DNA was removed, the anti-SRSF11 antibody or anti-Flag antibody and an equal amount of anti-IgG antibody were added to the lysis mixture, which was subsequently incubated at 4 °C for 16 h. Then, protein A/G beads were used to collect immunoprecipitates. Finally, TRIzol was used to extract RNA from immunoprecipitated samples and input samples. The extracted RNA was subsequently subjected to RT‒PCR and RIP‒seq. The primary antibodies used in this study are listed in Supplementary Table 5.

### RIP-seq

The extraction of SRSF11-binding RNA was performed as described above. High-quality RNA was broken into short fragments via fragmentation buffer, and a library was constructed via the KCTM Digital Stranded mRNA Library Prep Kit (Seqhealth Technology. Co., Ltd., Wuhan, China). The library was sequenced on a NovaSeq X Plus system in PE150 sequencing mode. The sequencing data were used to analyze SRSF-binding genes and motifs.

The detailed information of other experiments is available in supplementary file. These experiments include Expression and purification of recombinant proteins, In vitro succinylation assay, Lentivirus construction and infection, DNA construction and mutagenesis, Small interfering RNA (siRNA) and plasmid transfection, Mouse model, Co-IP, Analysis of alternative splicing, Reverse transcription‒polymerase chain reaction (RT‒PCR), Comet assays, Immunofluorescence staining, In situ Proximity Ligation Assay (PLA), Fluorescent In Situ Hybridization (FISH), Flow cytometry analysis of cell apoptosis and the cell cycle, Histology and immunohistochemistry (IHC), Western blotting, Colony formation assay, Bioinformatics analysis^51^.

### Statistical analysis

The data in this study were analyzed via SPSS 24.0 (IBM Corp.), and the figures were produced with GraphPad Prism 8.0 or R software (version 3.6.1). The data are expressed as the means ± standard deviations (SDs). The normal distribution of the data was assessed via the Kolmogorov‒Smirnov test. For normally distributed data, the data were analyzed via unpaired Student’s t test or one-way ANOVA followed by Bonferroni post hoc correction, where appropriate. For non-normally distributed data, the Kruskal-Wallis test was used to identify differences among more than two groups, and the Wilcoxon test was used to identify differences between two groups. The chi-square test was used to analyze the clinical correlations between gene expression levels and clinicopathological features. The Kaplan–Meier method was used to estimate survival rates, and the log-rank test was used to assess the differences between survival curves. All experiments were independently performed at least three times, and p < 0.05 was considered to indicate statistical significance.

## Supplementary information

Supplementary Materials

Supplementary Table 1

## Data Availability

All the other data supporting the findings of this study are available within the article and its Supplementary Information files. RNA-Seq and RIP-Seq datasets were deposited in the Gene Expression Omnibus (GEO) database under accession number GSE304384 for RIP-seq and GSE304385 for RNA-seq.
