# Supplementary material for: KAT2A-driven succinylation of SRSF11 enforces spliceosome-mediated RAD52 splicing to promote homologous recombination and radioresistance in hepatocellular carcinoma
Source: Signal Transduct Target Ther. 2025 Nov 7;10:364. doi: 10.1038/s41392-025-02458-7 (PMC12592546; doi:10.1038/s41392-025-02458-7)

**Supplementary Materials for**

**KAT2A-driven succinylation of SRSF11 enforces spliceosome-mediated RAD52 splicing to promote homologous recombination and radioresistance in hepatocellular carcinoma**

Jun Wu^1,2^, Jingsheng Yuan^1,2^, Zijian Liu^3^, Yongjie Zhou^1,2^, Bo Zhang^1,2,4^, Yahong Xu^5^, Qiwen Zeng^1,2^, Zhenru Wu^6^, Lingxiang Kong^1,2^, Jiaguo Wang^1,2^, Bohan Zhang^7^, Jian Yang^1,2^, Tao Lv^1,2^, Yujun Shi^1,2^, Jiayin Yang^1,2^

**Correspondence:**

Jiayin Yang. Email: [doctoryjy@scu.edu.cn](mailto:doctoryjy@scu.edu.cn). Phone: +1-86-028-85422867; Yujun Shi. Email: [shiyujun@scu.edu.cn](mailto:shiyujun@scu.edu.cn). Tao Lv, Email: [doclvtao@scu.edu.cn](mailto:doclvtao@scu.edu.cn).

**This file includes:**

Parts of materials and methods

Supplementary Figures 1 to 11

Supplementary Table 2 to 6

Uncropped western blots

**Parts of Materials and Methods**

**Expression and purification of recombinant proteins**

The cDNA encoding human SRSF11 and KAT2A were amplified by PCR and subsequently cloned into a pCMV-Flag vector, respectively. The recombinant vector was transformed into *E. coli*. A single colony was inoculated into LB medium supplemented with antibiotics and grown overnight at 37°C. The culture mixture was diluted (1:100) in fresh medium and incubated at 37°C. Protein expression was induced by adding 0.5 mM isopropyl-β-D-1-thiogalactopyranoside (IPTG), followed by further incubation at 16–25°C for 12–16h to increase solubility. The cells were harvested by centrifugation (6,000 × g, 10 min, 4°C). The cell pellet was resuspended in lysis buffer (20 mM Tris-HCl pH 7.5, 150 mM NaCl, 1 mM EDTA, 1% Triton X-100, and protease inhibitors) and lysed by sonication (3 × 30 sec pulses, 50% amplitude). The lysate was cleared by centrifugation (12,000 × g, 30 min, 4°C). The supernatant was incubated with Anti-Flag M2 affinity gel for 2h at 4°C with gentle rotation. The resin was washed extensively with TBS buffer (50 mM Tris-HCl pH 7.4, 150 mM NaCl) to remove nonspecific proteins. Bound Flag-SRSF11 was eluted via competitive elution with the Flag peptide (100–200 μg/mL), followed by neutralization (1 M Tris-HCl, pH 8.0). The incubation and elution of HA-KAT2A were performed using corresponding Anti-HA affinity gel and HA peptide.

***In vitro* succinylation assay**

Flag-tagged SRSF11 protein purified from *E. coli* was incubated with the indicated concentrations of succinyl-CoA in TBS buffer (150 mM NaCl and 50 mM Tris-HCl [pH 7.4]) at 37°C for the indicated times. Purified Flag-SRSF11, HA-KAT2A and the indicated concentrations of succinyl-CoA were added to a reaction buffer containing 50 mM Tris-HCl (pH 7.4), 2 mM ADP, 130 mM NaCl, 10 mM MgCl_2_, and 20 mM potassium phosphate and incubated at 37°C for the indicated times to confirm that SRSF11 was regulated by KAT2A.

**Lentivirus construction and infection**

The lentiviruses targeting SRSF11 and RAD52 were constructed by inserting their shRNAs between the BamHI and EcoRI sites of the psi-LVRU6GP vector (purchased from GeneCopoeia, Guangzhou, China), and the overexpression lentiviruses of the Tet-off system driven by full-length human SRSF11 wild-type and Mutant (K419R) were constructed by inserting them between the BamHI and EcoRI sites of the psi-LVRU6GP vector (purchased from GeneCodex, Wuhan, China). In this vector, SRSF11 expression was then inhibited by the addition of tetracycline (TET) to the medium (4 μg/mL). sgRNA targeting the SRSF11 gene and Cas9 vectors were cloned into LentiGuide Puro-EGFP and pLV3-U6-MCS-Cas9-Puro (purchased from WZBio., Shandong, China), respectively. The SRSF11 sgRNA sequence is: CAAGAAGCAGATCGAGACGG. Recombinant lentiviruses were amplified in 293T cells, and virus titers were determined by fluorescence and drug screening. After the lentiviral particles were purified via centrifugation, they were aliquoted and stored at -80°C. A lentivirus containing the empty vector psi-LVRU6GP was constructed as a control. The recombinant lentivirus was stably infected into the designated HCC cell line according to the manufacturer's instructions. The cells were cultured in this medium for 48–72h, after which the transfection efficiency was measured via RT‒PCR or western blotting.

**DNA construction and mutagenesis**

PCR-ampliﬁed human wild-type SRSF11 was subsequently cloned into pReceiver-Flag, and KAT2A and RAD52 were cloned into pReceiver-3xHA vectors, respectively (purchased from GeneCopoeia, Guangzhou, China). The site-speciﬁc mutantion of SRSF11, truncation mutation of RAD52 exon 10, and their overexpression plasmids used in our study were generated by GeneCodex (Wuhan, China). The minigene recombinant vectors were constructed by cloning a genomic DNA fragment containing the full-length coding sequence and native intronic regions flanking exon 10 into the GV712 plasmid. The minigene vectors were synthesized by Genechem (Shanghai, China).

**Small interfering RNA (siRNA) and plasmid transfection**

All siRNAs were synthesized by RiboBio (Guangzhou, China). The cells were seeded onto a six-well plate at a density of 50–70% 24 hours before transfection. For siRNA transfection, 4 µl of Lipo3000 reagent (Invitrogen, Carlsbad, MA, USA) and 4 µl of 20 nM siRNA were mixed in 200 µl of Opti-MEM™ medium (Invitrogen, Carlsbad, MA, USA) and then incubated at room temperature for 10 minutes. For plasmid transfection, 6 µl of P3000, 3 µg of plasmid and 125 µl of Opti-MEM™ medium were mixed. Moreover, 5 µl of Lipo3000 and 125 µl of Opti-MEM™ medium were mixed. Then, both two solutions were mixed and incubated at room temperature for 10 minutes. The mixture was directly added to cells in 0.8 ml of culture medium, and the transfection efficacy was assessed 48–72 hours later by RT‒PCR or western blotting. The targeting sequences of the siRNAs and shRNAs used in this study are listed in Supplementary Table 3 and Table 4.

**Mouse model**

BALB/c nude mice (female, 6–8 weeks old) were provided by Byrness Weil Biotech Ltd. (Chengdu, China). All the mice were housed in a speciﬁc pathogen-free environment on a controlled 12-hour light/dark cycle at 22–25°C and 60% humidity with the same food and water. The mice were injected with approximately 3× 105 of the indicated cells expressed luciferase. The cells were irradiated with 5Gy after treatment before injection. At least 5 mice per group were used in each experiment. Tumor growth was monitored weekly by measurements with calipers and visualized using an IVIS@ Lumina II system (Caliper Life Sciences, Hopkinton, MA) 15 min after intraperitoneal injection of 3.0 mg of D-Luciferin (ABP Biosicences, USA) in 200 μl of sterile PBS without magnesium or calcium. The formula 1/2×longest diameter×(shortest diameter) was used to calculate the tumor volume.

For the primary HCC mouse model, pT/Caggs-NRASV12 (25 μg/mouse), pT3-EF1αH-ΔN90-β-catenin (25 μg/mouse), and pCMV-SB (3 μg/mouse) were injected into the mouse liver via hydrodynamic tail vein injection as described previously. Chemically induced HCC was established via the intraperitoneal injection of diethylnitrosamine (DEN) (Sigma) into 2-week-old male C57BL/6 pups at a dose of 20 mg/g body weight. Then, the mice were given a weekly i.p. injection of 0.2 mL/g body weight CCL4 (Cat#289116, Sigma) dissolved in olive oil (Cat#O1514, Sigma) at a 1:10 (v/v) ratio as described previously.

**Co-IP**

A Co-IP kit (Absin, Shanghai, China) was used for the Co-IP assay according to the manufacturer's instructions. Briefly, the cells were lysed using IP lysis buffer on ice, and the supernatant was collected by centrifugation at 12000 rpm for 10 minutes at 4°C. Then, approximately 500–1000μg of extract was incubated with the corresponding primary or IgG antibody overnight at 4°C with rotation. Next, rProtein A/G MagPoly Beams were added and incubated at room temperature for 2 h. After sufficient elution, the immunoprecipitate was used for subsequent detection. The primary antibodies used in this study are listed in Supplementary Table 5.

**Analysis of alternative splicing**

Alternative splicing analysis was performed on the basis of the above RNA-seq data. RMATS (version 4.1.1) was used to identify and analyze different splicing events. These splicing events include skipped exon (SE), mutually exclusive exon (MXE), alternative 5’ splice site (A5SS), alternative 3’splice site (A3SS) and retained intron (RI). Percent spliced-in (PSI) values are used to calculate the splicing level and are defined as inclusion/(inclusion+exclusion). P<0.05 and |ΔPSI|>0.1 were considered to indicate significant splicing events. The differential splicing events were ultimately determined via RT‒PCR.

**Reverse transcription‒polymerase chain reaction (RT‒PCR)**

The RNA bound to SRSF11 was obtained through the RIP assay described above. Total RNA from cells and tissues was extracted using the FastPure Cell/Tissue Total RNA Isolation Kit V2 (Vazyme) according to the manufacturer’s instructions. The extracted RNA was reverse transcribed into cDNA using the iScript^TM^ cDNA Synthesis Kit (Bio-Rad), and the reaction conditions were as follows: 25°C for 5 min, 46°C for 20 min, 95°C for 1 min and hold at 4°C. PCR was subsequently performed with the 2×Taq PCR MasterMix II kit (TIANGEN), and the reaction conditions were as follows: 94°C for 3 min; followed by 34 cycles of 94°C for 30s, 58°C for 30s and 72°C for 40–90s based on the length of the products. The amplified product was separated using 1.5% agarose gels stained with Goldview and observed for bands via a UV imaging system. Finally, ImageJ was used to calculate the integrated optical density of the bands. The primers used in this study are shown in Supplementary Table 6.

**Comet assays**

Comet assay kits (Beyotime, Shanghai, China) were used to detect DNA damage according to the manufacturer’s instructions. In brief, approximately 10^6^ cells/ml were collected, and 10 µl of cells (approximately 104 cells) were mixed with 75µl of 0.7% low melting agarose in a 37°C water bath. The mixture was then placed onto a comet slide and transferred to a refrigerator at 4°C for 10 minutes to embed the cells. Precooled lysis solution was added, and the cells were lysed at 4°C for 1–2 hours. Afterward, the slide was placed in electrophoresis buffer at room temperature for 40 minutes to unwind the DNA double helix. Then, low-voltage (25 V) electrophoresis was performed for 30 minutes. Propidium iodide solution was then added for staining. Finally, the tail moment was observed and measured under a fluorescence microscope for assessment of DNA damage.

**Immunoﬂuorescence staining**

The cells were seeded into a 24-well plate with glass slides 12 hours before transfection. The transfected cells were fixed with 4% paraformaldehyde for 30 minutes and washed three times with PBS. After cell permeabilization with 0.5% Triton X-100 at room temperature for 20 minutes, peroxidase blocking solution was added for blocking. Afterward, the diluted primary antibody was added to the slides, which were subsequently incubated overnight at 4°C. After the samples were washed with PBST three times, secondary antibodies were added, and the samples were incubated at room temperature for 30 minutes. Then, fluorescent dye was added, and the mixture was incubated for 10 minutes. If a second target protein needs to be stained, antibody stripping solution was used for treatment and then the above steps to incubate the primary and secondary antibodies were repeated, followed by another different colored fuel. Finally, DAPI was used to stain the cell nucleus, and the slides were sealed with blocking solution. The images were captured using a Zeiss LSM 980 confocal microscope. The primary antibodies used in this study are listed in Supplementary Table 5.

***In situ* Proximity Ligation Assay (PLA)**

The PLA kit (DU092101-1KT, Sigma, USA) was used for detecting protein interaction according to the manufacturer’s instructions. In briefly, the cells were seeded into a 24-well plate with glass slides 12 hours before transfection. The cells were fixed with 4% paraformaldehyde for 30 minutes and washed three times with PBS. After cell permeabilization with 0.5% Triton X-100 at room temperature for 20 minutes, Duolink® blocking solution was added for blocking 30 min at 37℃. Afterwards, primary antibodies from different species were incubated at 4 ° C overnight. After washing with washing buffer A, Duolink® PLUS and MINUS PLA probe were incubated at 37 ° C for 1 h. Next, the ligase was incubated at 37 ° C for 30 min. Then, polymerase was added and incubated at 37 ° C for 100 minutes for amplification. Finally, DAPI was used to stain the cell nucleus, and the slides were sealed with blocking solution. The images were captured using a Zeiss LSM 980 confocal microscope. The primary antibodies used in this study are listed in Supplementary Table 5.

**Fluorescent In Situ Hybridization (FISH)**

RNA FISH Kit (RiboBio, Guangzhou, China) was used to detect the exon 10 of RAD52. The RNA probe with red fluorescence targeting exon 10 was designed and synthesized by RuiboBio. The paraffin-embedded HCC tissues were cut into 4μm thin slices and then placed on glass slides. Xylene and a series of gradient ethanol solutions were used for dewaxing and rehydration. Protease K (20ug/ml) was used for digestion of slices at 37 ° C for 20 minutes. After washing with PBS three times, hybridization solution was added and incubated at 37 ° C for 30 minutes. Then, the probe was added and hybridized overnight at 37 ° C. After washing with SSC solution and PBS, DAPI was used to stain the cell nucleus, and the slides were sealed with blocking solution. The images were captured using a Zeiss LSM 980 confocal microscope. All reagents and PBS were sterile and RNAse-free.

**Flow cytometry analysis of cell apoptosis and the cell cycle**

An Annexin V-FITC/PI Apoptosis Kit (Elabscience, Wuhan, China) was used for the apoptosis assay. First, the cells were collected and washed with PBS, and 100 μL of diluted 1 × Annexin V binding buffer was added to resuspend the cells. Each sample was incubated with 2.5 μL of Annexin V-FITC reagent and 2.5 μL of PI reagent (50μg/mL) at room temperature in the dark for 15–20 minutes. Apoptosis was detected via a CytoFLEX flow cytometer (Beckman Coulter). Both early and late apoptotic cells were counted for apoptosis analysis.

A cell cycle distribution assay was performed with a Cell Cycle Assay Kit (Elabscience, Wuhan, China). The cells were collected and fixed with 80% ethanol overnight at -20°C. After being washed with PBS, the cells were incubated with RNase A Reagent in a 37°C water bath for 30 minutes. Each sample was subsequently incubated with 400 μL of PI Reagent at 2–8°C in the dark for 30 minutes. Finally, the cell cycle distribution was detected via a CytoFLEX flow cytometer (Beckman Coulter) at a wavelength of 488 nm.

**Histology and immunohistochemistry (IHC)**

The paraffin-embedded HCC tissues were cut into 4μm thin slices and then placed on glass slides. Xylene and a series of gradient ethanol solutions were used for dewaxing and rehydration. Antigen retrieval of the slices was performed with 0.01 M citric acid buffer (pH 6.0) at high temperature. After the samples were blocked with 5% BSA for 30 minutes, they were incubated with the primary antibody overnight at 4°C. After being washed with PBS three times, the slides were incubated with secondary antibody at room temperature for 1 hour and stained with diaminobenzidine (DAB). The IHC results were scored by two independent pathologists according to both the staining intensity (scored from 0 to 3) and the percentage of positively stained cells (scored from 0 to 3), and the final immunoreactivity score (IRS, range 0–9) was obtained by multiplying the two scores. The expression of a given protein was considered low if the score was less than 5 and high if the score was 5 or higher. The primary antibodies used in this study are listed in Supplementary Table 5.

**Western blotting**

RIPA lysis buffer supplemented with protease inhibitor and phenylmethylsulfonyl fluoride was used to extract all the proteins from the tissues and cells. The protein concentration was measured with a BCA assay kit. Then, protein samples were mixed with protein loading buffer and boiled at 100°C for 15 minutes for denaturation. The denatured protein was separated via 8–12% SDS‒PAGE based on its molecular weight and then transferred onto a PVDF membrane. After the membrane was blocked at room temperature for 1 hour with 5% skim milk, it was incubated with the primary antibody overnight at 4°C. GAPDH was used as an internal reference protein. A specific antibody that specifically recognized SRSF11 with succinylation at K419 was designed and produced by HuaBio (Hangzhou, China). Next, the PVDF membranes were washed three times with TBST for 10‒15 minutes each time. The membranes were subsequently incubated with an HRP-conjugated AﬃniPure secondary antibody at room temperature for 1 hour at a dilution ratio of 1:5000. Finally, the membranes were washed with TBST three times, and protein bands visualized with enhanced chemiluminescence (ECL) solution. The dilution ratio and detailed information on all primary antibodies are available in the Supplementary Table 5.

**Colony formation assay**

The cells were seeded in 12-well plates at a density of 1000 cells per well and treated with indicated dose of IR (0–8 Gy, IR exposure durations: 27 seconds for 2 Gy, 53 seconds for 4 Gy, 80 seconds for 6 Gy, and 106 seconds for 8 Gy). After 10–14 days of cultivation, the colonies were fixed with 4% paraformaldehyde, stained with crystal violet, and counted.

**Bioinformatics analysis**

The transcriptome, proteome, and clinical data of HCC patients from The Cancer Genome Atlas (TCGA) were obtained from the UCSC Xena database (https://xena.ucsc.edu/). scRNA-seq data (GSE166635) were obtained from the Gene Expression Omnibus (GEO) database and preprocessed as described previously ^50^. The radiosensitivity index, DNA damage repair score and HR score data for patients from the TCGA-LIHC and GEO cohorts were obtained to evaluate the effects of SRSF11 on radiosensitivity, DNA damage repair and HR. Spearman’s correlation analysis was performed to calculate the correlation coefficient between two factors. To assess the correlation between gene expression and patient survival, the optimal cutoff point for each dataset was determined using the “survminer” R package, and the “surv-cutpoint” function was used to repeat all potential cutoff points to obtain the maximum rank statistic; ultimately, the patients were divided into two groups (the high expression group and the low expression group) for each factor of interest. Survival curves were generated for prognosis analysis using the Kaplan–Meier method, and the significance of differences were identified using the log-rank test.

**Supplemental Figures and Figure legends:**


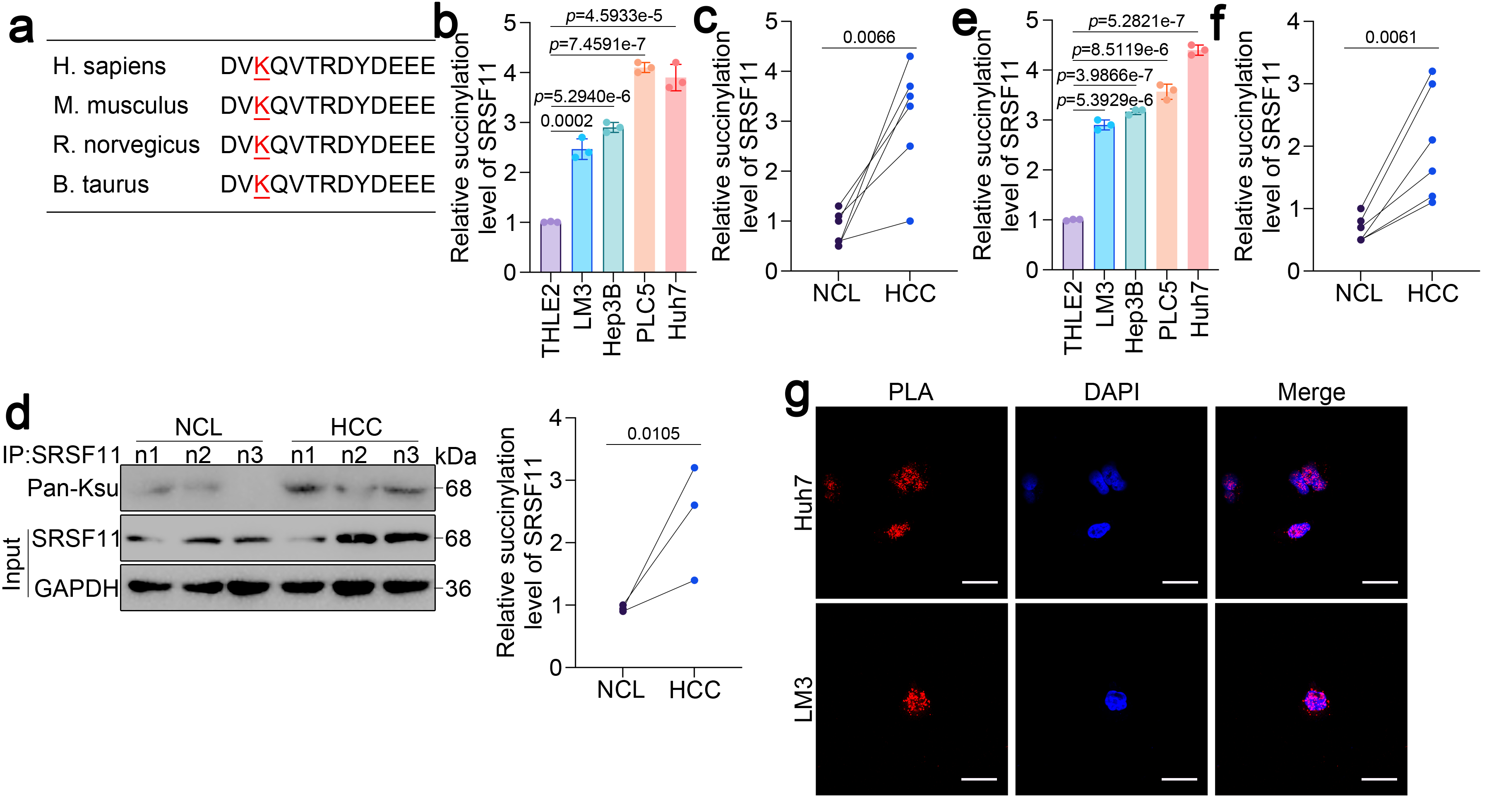


**Supplementary Figure 1 KAT2A drives oncogenic SRSF11 succinylation at K419. a.** Conservation analysis of SRSF11 succinylation at K419 in different species, ranging from *H. sapiens to B. taurus*. **b**. Relative succinylation level of SRSF11 in HCC cells and THLE2 cells examined with anti-pan-Ksu antibody (measured as the ratio of succinylation to total SRSF11 protein by band density) in each group. **c**. Relative succinylation level of SRSF11 in HCC and NCL tissues examined with anti-pan-Ksu antibody (measured as the ratio of succinylation to total SRSF11 protein by band density) in each group. **d**. The succinylation level of SRSF11 examined with anti-pan-Ksu antibody in mouse primary HCC model. **e**. Relative succinylation level of SRSF11 in HCC cells and THLE2 cells examined with anti-SRSF11^Ksu^ antibody (measured as the ratio of succinylation to total SRSF11 protein by band density) in each group. **f**. Relative succinylation level of SRSF11 in HCC and NCL tissues examined with anti-SRSF11Ksu antibody (measured as the ratio of succinylation to total SRSF11 protein by band density) in each group. **g**. The interaction analysis of SRSF11 and KAT2A by PLA assay. For b and e, the data are presented as the means ± SDs, the statistical analyses were performed via two-tailed unpaired Student’s t-tests. For c, f and d, the statistical analyses were performed via ‌ paired Student’s t-tests. For d and g, 3 independent experiments (n = 3) with similar results were performed in triplicate. p < 0.05 was considered to indicate statistical significance.


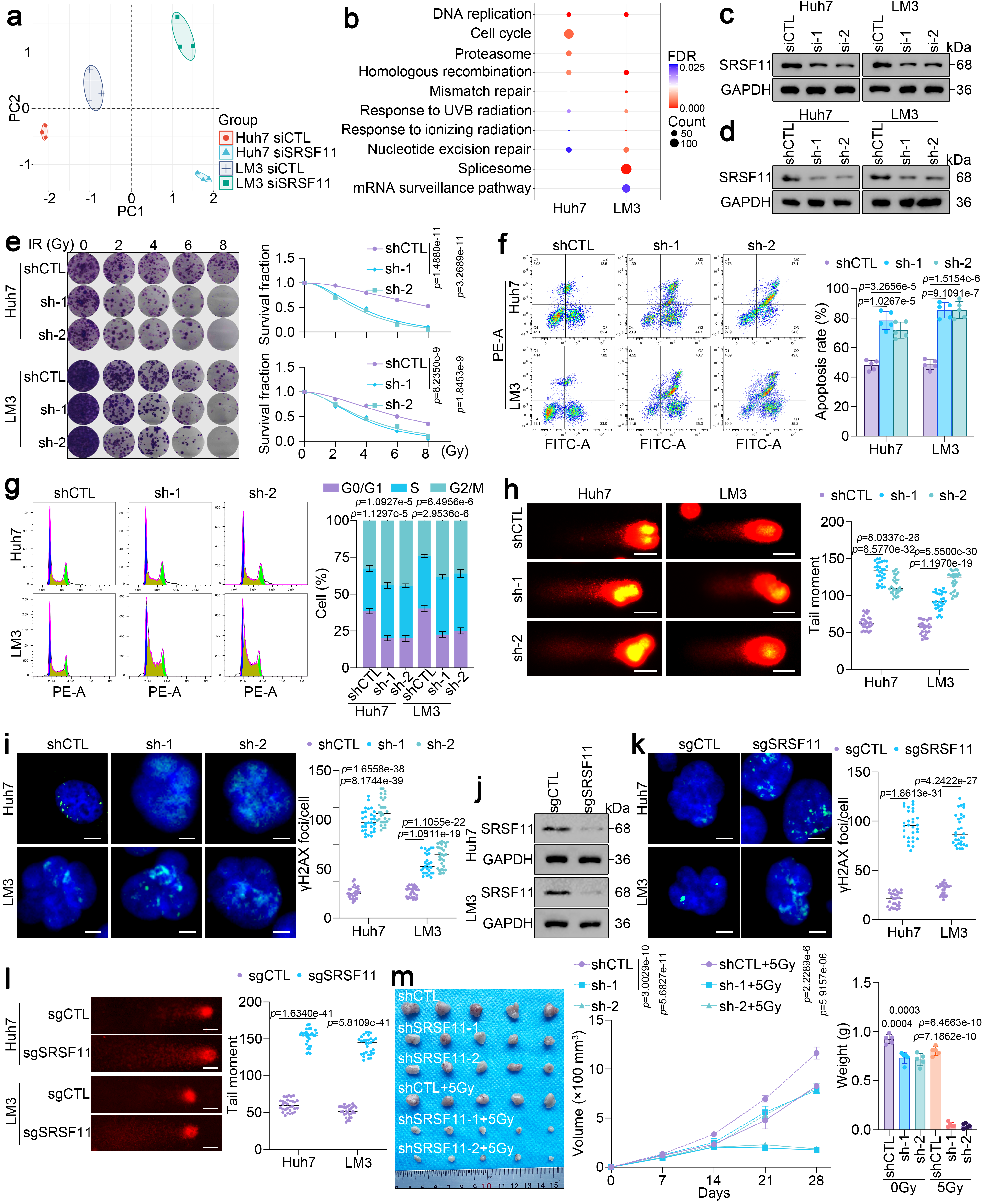


**Supplementary Figure 2 SRSF11 inhibition increases DNA damage and radiotherapy sensitivity. a.** Principal component analysis in Huh7 and LM3 cells transfected with siSRSF11 using RNA transcriptome sequencing. b. GSEA analysis of differentially expressed genes in Huh7 and LM3 cells. **c, d** The knockdown efficiency verification of SRSF11 by siRNAs (**c**) and shRNAs (**d**) in Huh7 and LM3 cells. **e**. Colony formation assays of LM3 and Huh7 cells transfected with shSRSF11 and treated with the indicated dose of IR. **f, g**. Flow cytometry analysis for apoptosis (**f**) and cell cycle (**g**) treated with 5Gy of IR in Huh7 and LM3 cells transfected with shSRSF11. **h**. Comet assay of Huh7 and LM3 cells transfected with shSRSF11 and treated with 5Gy of IR. Scale bars, 10 μm. **i**. Immunofluorescence staining for γ-H2AX (green) and DAPI (blue) in LM3 and Huh7 cells transfected with shSRSF11 after IR by 5Gy. Scale bars, 10 μm. **j**. The knockout efficiency verification of SRSF11 by sgRNAs. **k**. Immunofluorescence staining for γ-H2AX (green) and DAPI (blue) in LM3 and Huh7 cells transfected with sgSRSF11 after IR by 5Gy. Scale bars, 10 μm. **l**. Comet assay of Huh7 and LM3 cells transfected with sgSRSF11 and treated with 5Gy of IR. Scale bars, 10 μm. **m.** Representative images of the xenograft, tumour volume, and tumor weight using LM3 cells transfected with shSRSF11 and treated without/with IR. For c‒m, 3 independent experiments (n = 3) with similar results were performed in triplicate. For e-m, the data are presented as the means ± SDs. The statistical analyses were performed via two-tailed unpaired Student’s t-tests. p < 0.05 was considered to indicate statistical significance.


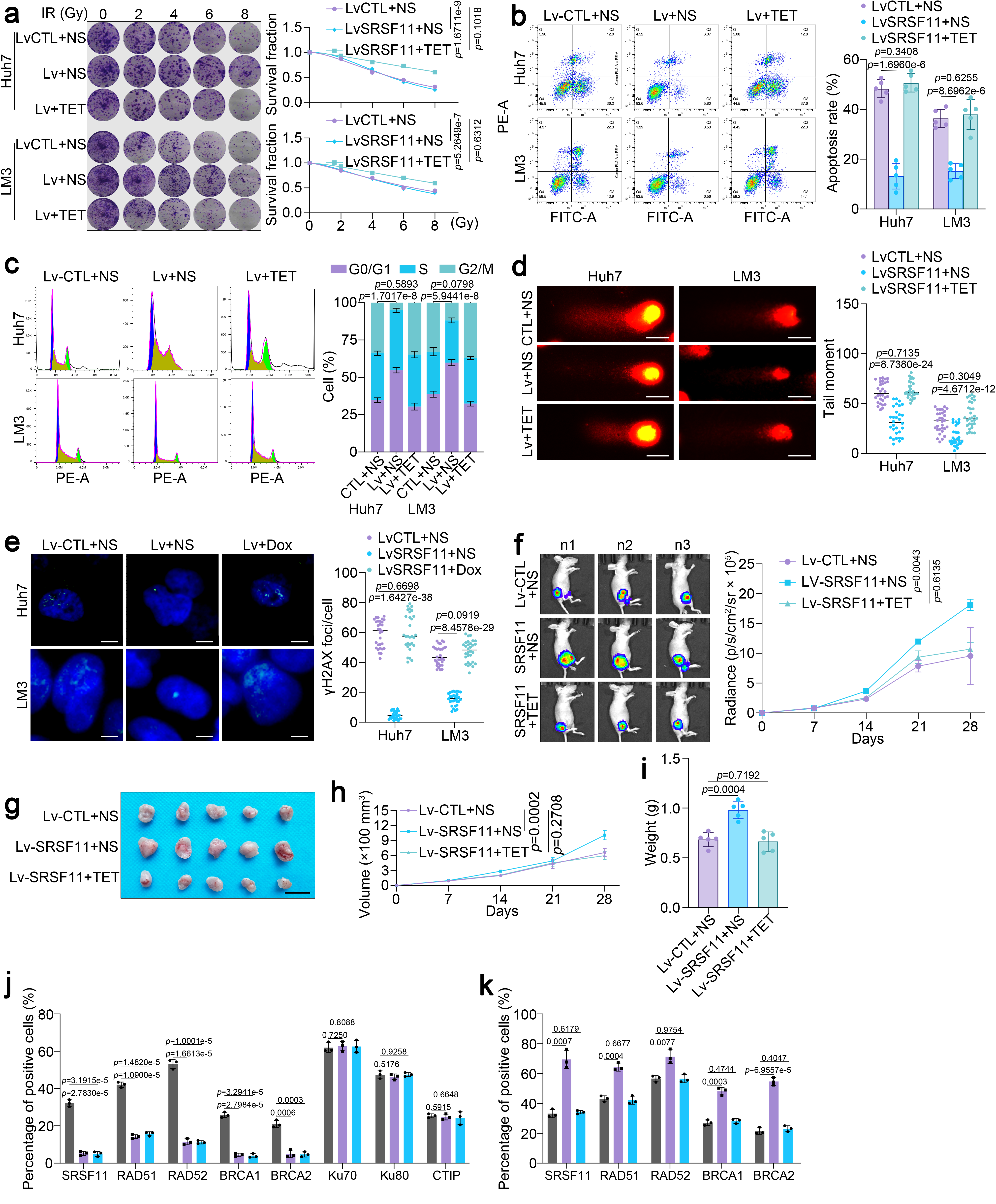


**Supplementary Figure 3 SRSF11 promotes DNA DSBs repair by HR *in vivo*. a.** Colony formation assays of LM3 and Huh7 cells infected with Lv-SRSF11 treated with/without TET and the indicated dose of IR. **b, c**. Flow cytometry analysis of apoptosis (**b**) and the cell cycle (**c**) in Huh7 and LM3 cells infected with Lv-SRSF11 and treated with/without TET and 5Gy of IR. **d**. Comet assay of Huh7 and LM3 cells infected with Lv-SRSF11 and treated with/without TET and 5Gy of IR. Scale bars, 10 μm. **e**. Immunofluorescence staining for γ-H2AX (green) and DAPI (blue) in LM3 and Huh7 cells infected with Lv-SRSF11 treated with/without TET and 5Gy of IR. Scale bars, 10 μm. **f**. Luciferase signals of xenograft model in nude mice using LM3 cells infected with Lv-SRSF11 and treated with/without TET and 5Gy of IR. (n=5 per group). **g-i.** Representative images of the xenograft, tumour volume, and tumor weight using LM3 cells infected with Lv-SRSF11 and treated with/without TET. Scale bars, 1 cm. **j**. Quantitative analysis of IHC staining for the HR-related proteins RAD51, RAD52, BRCA1, and BRCA2 and the NHEJ-related proteins Ku70, Ku80, and CTIP in xenograft tumors infected with/without shSRSF11. **k**. Quantitative analysis of IHC staining for the HR-related proteins RAD51, RAD52, BRCA1, and BRCA2 in xenograft tumors infected with Lv-SRSF11 and treated with/without TET. For a‒e, 3 independent experiments (n=3) with similar results were performed in triplicate. For a‒f and h‒k, the data are presented as the means ± SDs. The statistical analyses were performed via two-tailed unpaired Student’s t-tests. p < 0.05 was considered to indicate statistical significance.


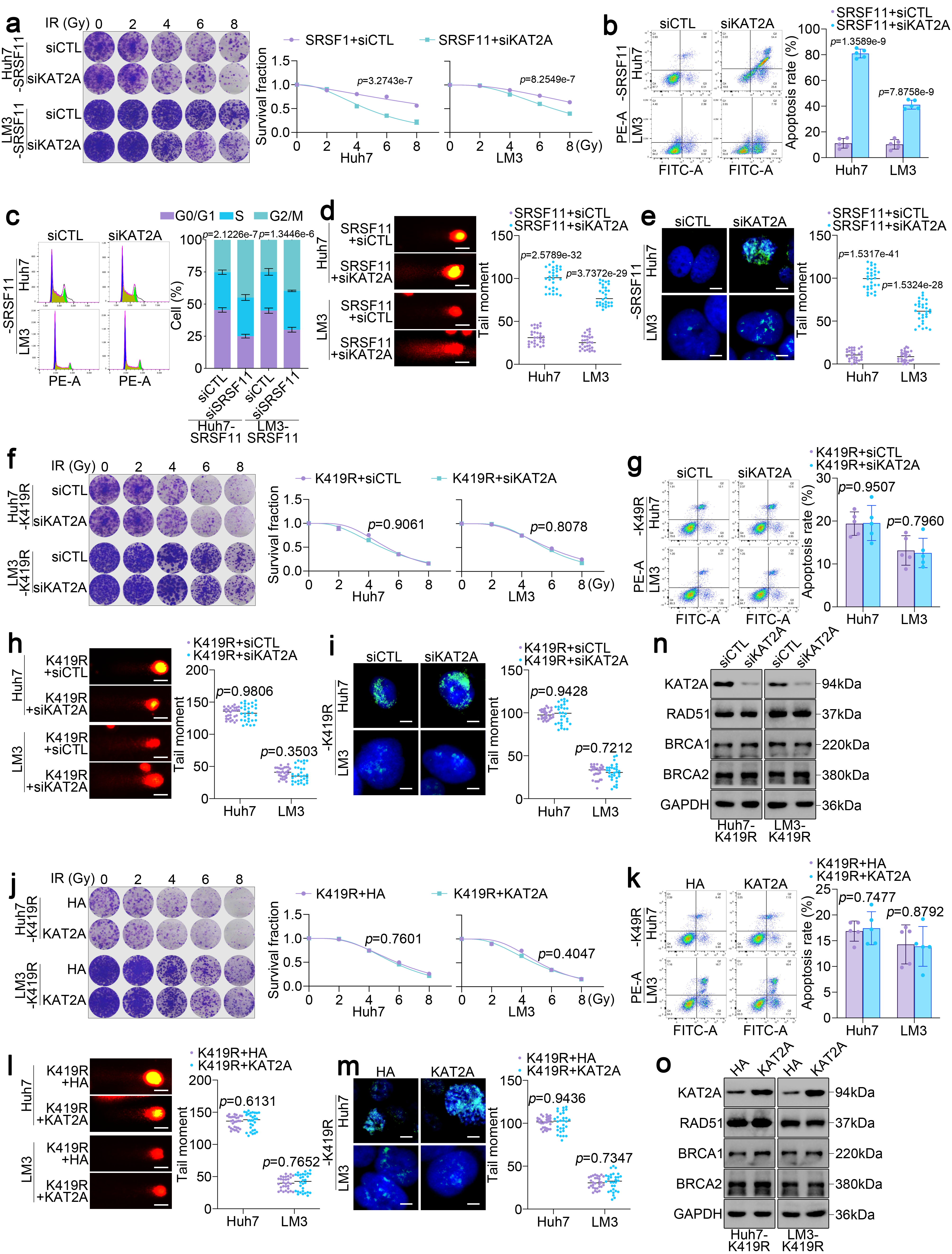


**Supplementary Figure 4** **KAT2A-induced succinylation at K419 regulates SRSF11-mediated HR a**. Colony formation assays in SRSF11-overexpressing LM3 and Huh7 cells transfected with siKAT2A and treated with the indicated dose of IR. **b, c**. Flow cytometry analysis of apoptosis (**b**) and the cell cycle (**c**) in SRSF11-overexpressing LM3 and Huh7 cells transfected with siKAT2A and treated with 5Gy of IR. **d**. Comet assay of SRSF11-overexpressing LM3 and Huh7 cells transfected with siKAT2A and treated with 5Gy of IR. Scale bars, 10 μm. **e**. Immunofluorescence staining for γ-H2AX (green) and DAPI (blue) in SRSF11-overexpressing LM3 and Huh7 cells transfected with siKAT2A and treated with 5Gy of IR. Scale bars, 10 μm. **f**. Colony formation assays in SRSF11 K419R-overexpressed LM3 and Huh7 cells transfected with siKAT2A and treated with the indicated dose of IR. **g**. Flow cytometry analysis for apoptosis in SRSF11 K419R-overexpressed LM3 and Huh7 cells transfected with siKAT2A and treated with 5Gy of IR. **h**. Comet assay of SRSF11 K419R-overexpressed LM3 and Huh7 cells transfected with siKAT2A and treated with 5Gy of IR. Scale bars, 10 μm. **i**. Immunofluorescence staining for γ-H2AX (green) and DAPI (blue) in SRSF11 K419R-overexpressed LM3 and Huh7 cells transfected with siKAT2A and treated with 5Gy of IR. Scale bars, 10 μm. **j**. Colony formation assays in SRSF11 K419R-overexpressed LM3 and Huh7 cells transfected with HA-KAT2A and treated with the indicated dose of IR. **k**. Flow cytometry analysis for apoptosis in SRSF11 K419R-overexpressed LM3 and Huh7 cells transfected with HA-KAT2A and treated with 5Gy of IR. **l**. Comet assay of SRSF11 K419R-overexpressed LM3 and Huh7 cells transfected with HA-KAT2A and treated with 5Gy of IR. Scale bars, 10 μm. **m**. Immunofluorescence staining for γ-H2AX (green) and DAPI (blue) in SRSF11 K419R-overexpressed LM3 and Huh7 cells transfected with HA-KAT2A and treated with 5Gy of IR. Scale bars, 10 μm. **n**. Western blotting assay of the expression of RAD51, BRCA1 and BRCA2 in SRSF11 K419R-overexpressed LM3 and Huh7 cells transfected with siKAT2A and treated with 5Gy of IR. **o**. Western blotting assay of the expression of RAD51, BRCA1 and BRCA2 in SRSF11 K419R-overexpressed LM3 and Huh7 cells transfected with HA-KAT2A and treated with 5Gy of IR. For all the above experiments, the data are presented as the means ± SDs. 3 independent experiments (n=3) with similar results were performed in triplicate. The statistical analyses were performed via two-tailed unpaired Student’s t-tests. p < 0.05 was considered to indicate statistical significance.


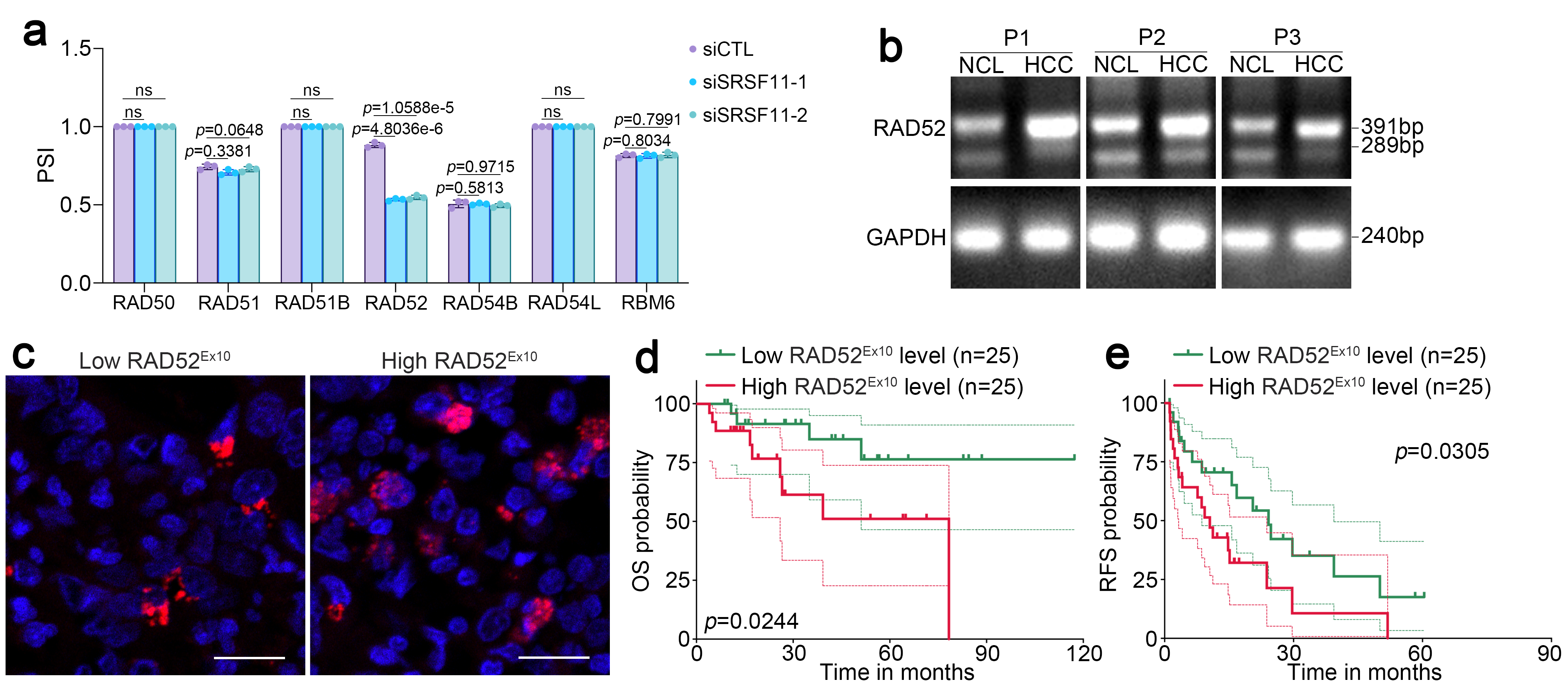


**Supplementary Figure 5 SRSF11 promotes the inclusion of RAD52 exon 10. a.** The PSI [PSI=inclusion/(inclusion+exclusion)] analysis of RAD50, RAD51, RAD51B, RAD52, RAD54B, RAD54L and RBM6 in Huh7 transfected with siSRSF11. **b**. RT‒PCR detection of exon 10 skipping of RAD52 in NCL and HCC tissues of patients. **c**. Representative images of RNA *In Situ* Hybridization specifically targeting exon 10 of RAD52 in HCC tissues of patients. **d, e**. OS and RFS curves for high expression and low expression group of RAD52 with exon10 from our clinical cohort. For a, the data are presented as the means ± SDs. For a-b, 3 independent experiments (n = 3) with similar results were performed in triplicate. The statistical analyses were performed via two-tailed unpaired Student’s t-tests. p < 0.05 was considered to indicate statistical significance.


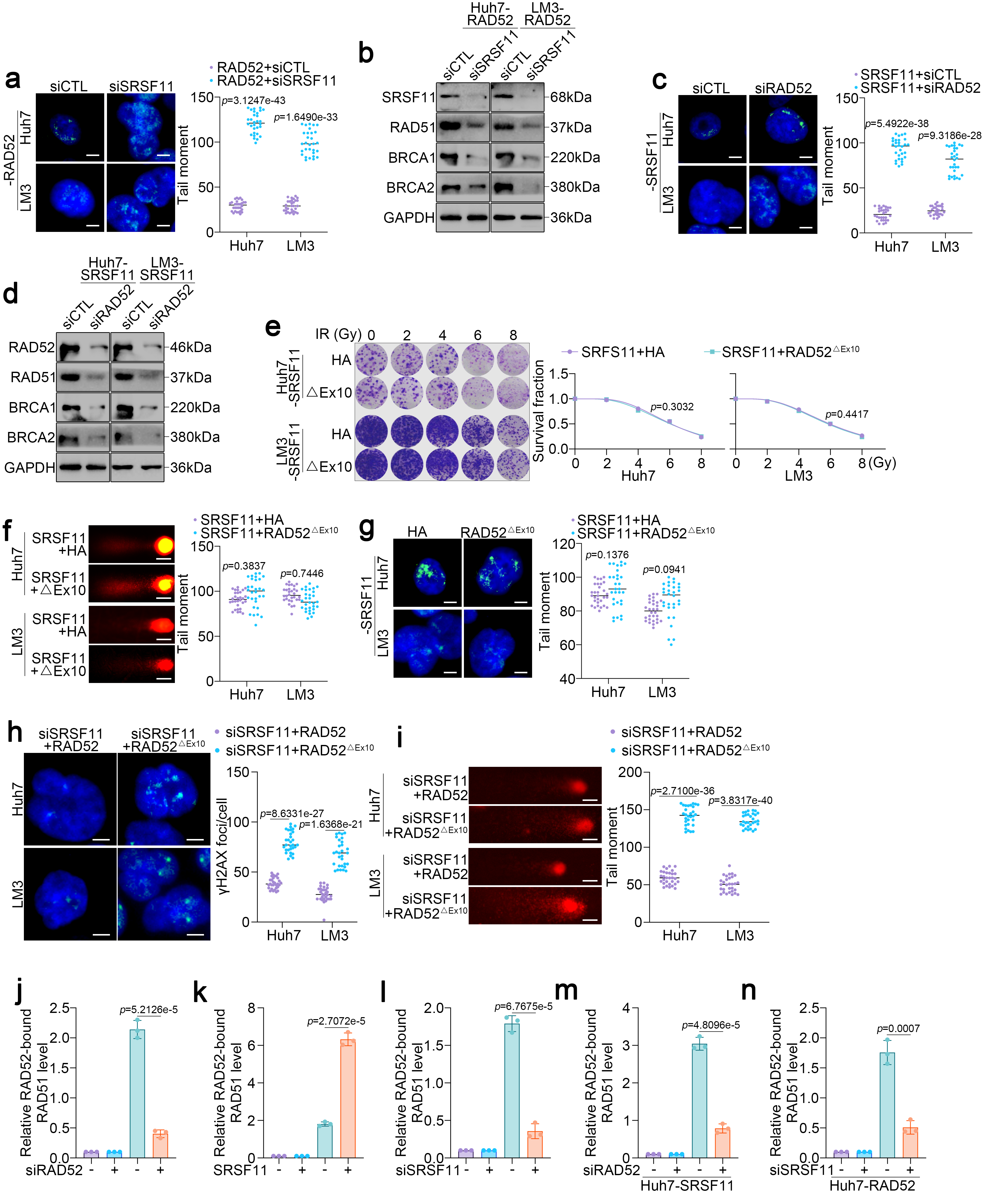


**Supplementary Figure 6 SRSF11 promotes RAD52/RAD51 dimer formation by regulating RAD52 splicing. a.** Immunofluorescence staining for γH2AX (green) and DAPI (blue) in RAD52-overexpressed Huh7 and LM3 cells transfected with siSRSF11 and treated with 5Gy of IR. Scale bars, 10 μm. **b**. Western blotting assay of RAD51, BRCA1 and BRCA2 in RAD52-overexpressed Huh7 and LM3 cells transfected with siSRSF11 and treated with 5Gy of IR. **c**. Immunofluorescence staining for γ-H2AX (green) and DAPI (blue) in SRSF11-overexpressed Huh7 and LM3 cells transfected with siRAD52^Ex10^ and treated with 5Gy of IR. Scale bars, 10 μm. **d**. Western blotting assay of of RAD51, BRCA1 and BRCA2 in SRSF11-overexpressed Huh7 and LM3 cells transfected with siRAD52^Ex10^ and treated with 5Gy of IR. **e**. Colony formation assays of SRSF11-overexpressed LM3 and Huh7 cells transfected with HA-RAD52^ΔEx10^ and treated with the indicated dose of IR. **f**. Comet assay of SRSF11-overexpressed LM3 and Huh7 cells transfected with HA-RAD52^ΔEx10^ and treated with 5Gy of IR. Scale bars, 10 μm. **g**. Immunofluorescence staining for γ-H2AX (green) and DAPI (blue) in SRSF11-overexpressed LM3 and Huh7 cells transfected with HA-RAD52^ΔEx10^ and treated with 5Gy of IR. Scale bars, 10 μm. **h**. Immunofluorescence staining for γH2AX (green) and DAPI (blue) in HA-RAD52- or HA-RAD52^ΔEx10^ overexpressed Huh7 and LM3 cells transfected with siSRSF11 and treated with 5Gy of IR. Scale bars, 10 μm. **i**. Comet assay of HA-RAD52- or HA-RAD52^ΔEx10^ overexpressed Huh7 and LM3 cells transfected with siSRSF11 and treated with 5Gy of IR. Scale bars, 10 μm. **j**. Quantitative analysis for the binding of RAD52 with RAD51 (measured as the ratio of RAD51, RAD52 to their expression level in input by band density) after immunoprecipitation with an anti-RAD52 antibody in Huh7 cells transfected with or without siRAD52^Ex10^. **k**. Quantitative analysis for the binding of RAD52 with RAD51 after immunoprecipitation with an anti-RAD52 antibody in Huh7 cells transfected with or without Flag-SRSF11. **l**. Quantitative analysis for the binding of RAD52 with RAD51 after immunoprecipitation with an anti-RAD52 antibody in Huh7 cells transfected with or without siSRSF11. **m**. Quantitative analysis for the binding of RAD52 with RAD5 after immunoprecipitation with an anti-RAD52 antibody in SRSF11-overexpressing Huh7 cells transfected with or without siRAD52^Ex10^. **n**. Quantitative analysis for the binding of RAD52 with RAD51 after immunoprecipitation with an anti-RAD52 antibody in RAD52-overexpressing Huh7 cells transfected with or without siSRSF11. For a-i, 3 independent experiments (n = 3) with similar results were performed in triplicate. For a, c and e‒n, the data are presented as the means ± SDs. The statistical analyses were performed via two-tailed unpaired Student’s t-tests. p < 0.05 was considered to indicate statistical significance.

**
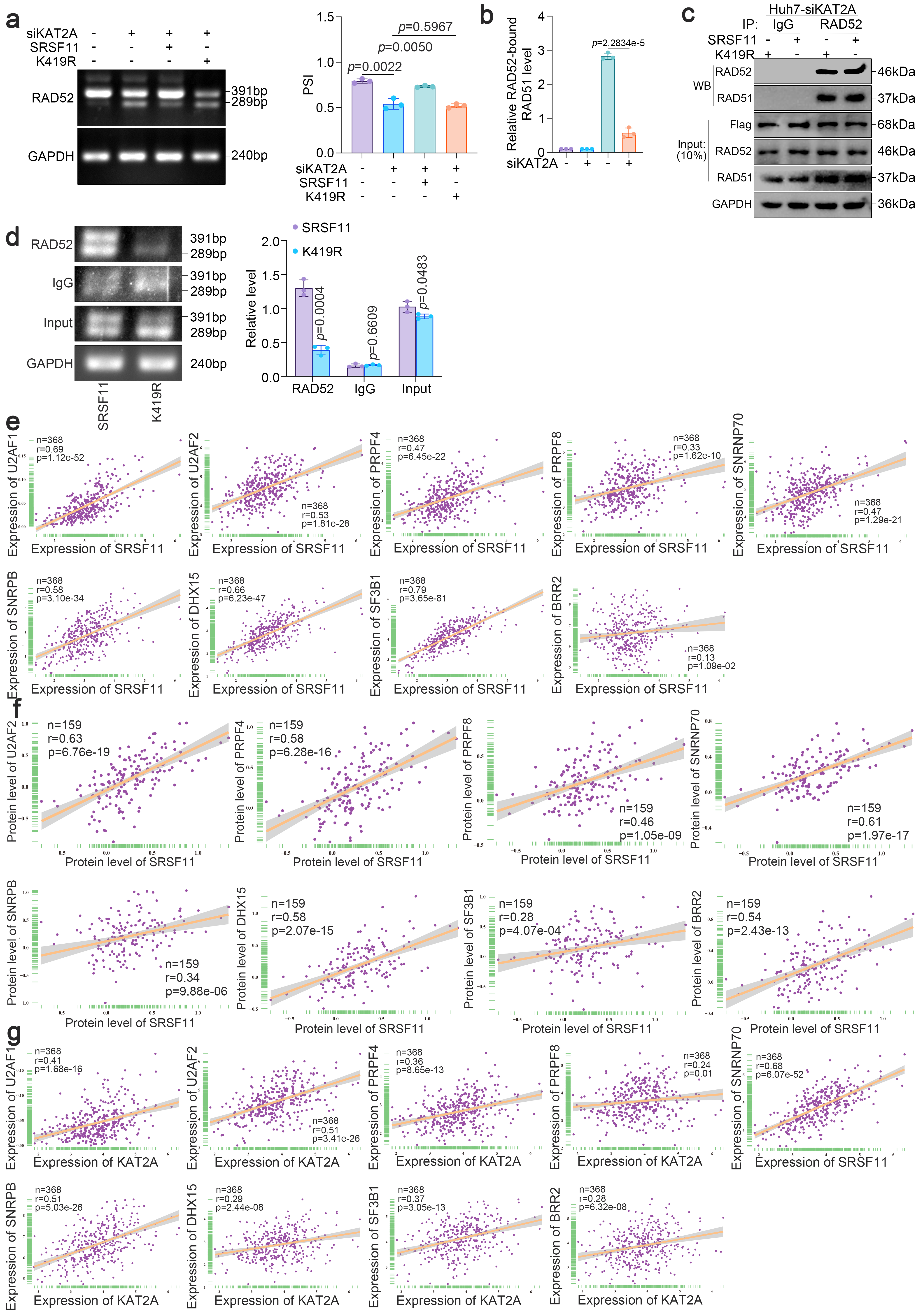
**

**Supplementary Figure 7 KAT2A-mediated K419 succinylation of SRSF11 promote spliceosome formation. a.** RT-PCR detection the exon 10 skipping of RAD52 in Huh7 cells transfected with Flag-SRSF11 or Flag-SRSF11 K419R and with/without siKAT2A. **b**. Quantitative analysis for the binding of RAD52 with RAD51 (measured as the ratio of RAD51, RAD52 to their expression level in input by band density) after immunoprecipitation with an anti-RAD52 antibody in Huh7 cells transfected with or without siKAT2A. **c**. Western blotting assay of the binding of RAD51 with RAD52 after immunoprecipitation with an anti-RAD52 antibody in Huh7 cells transfected with Flag-SRSF11 or Flag-SRSF11 K419R after knocking down KAT2A. **d**. RIP and RT‒PCR detection of RAD52 pre-mRNA binding to SRSF11 by anti-Flag antibody in Huh7 cells transfected with Flag-SRSF11 or Flag-SRSF11 K419R. **e**. Correlation analysis of the mRNA expression of SRSF11 with U2AF1, U2AF2, PRPF4, PRPF8, SNRNP, SNRPB, DHX15, SF3B1 and BRR2 from TCGA-LIHC database. **f**. Correlation analysis of the protein expression of SRSF11 with U2AF1, U2AF2, PRPF4, PRPF8, SNRNP, SNRPB, DHX15, SF3B1 and BRR2 from TCGA-LIHC database. **g**. Correlation analysis of the mRNA expression of KAT2A with U2AF1, U2AF2, PRPF4, PRPF8, SNRNP, SNRPB, DHX15, SF3B1 and BRR2 from the TCGA-LIHC database. For a and c-d, 3 independent experiments (n = 3) with similar results were performed in triplicate. For a‒b and d, the data are presented as the means ± SDs. The statistical analyses were performed via two-tailed unpaired Student’s t-tests. p < 0.05 was considered to indicate statistical significance.


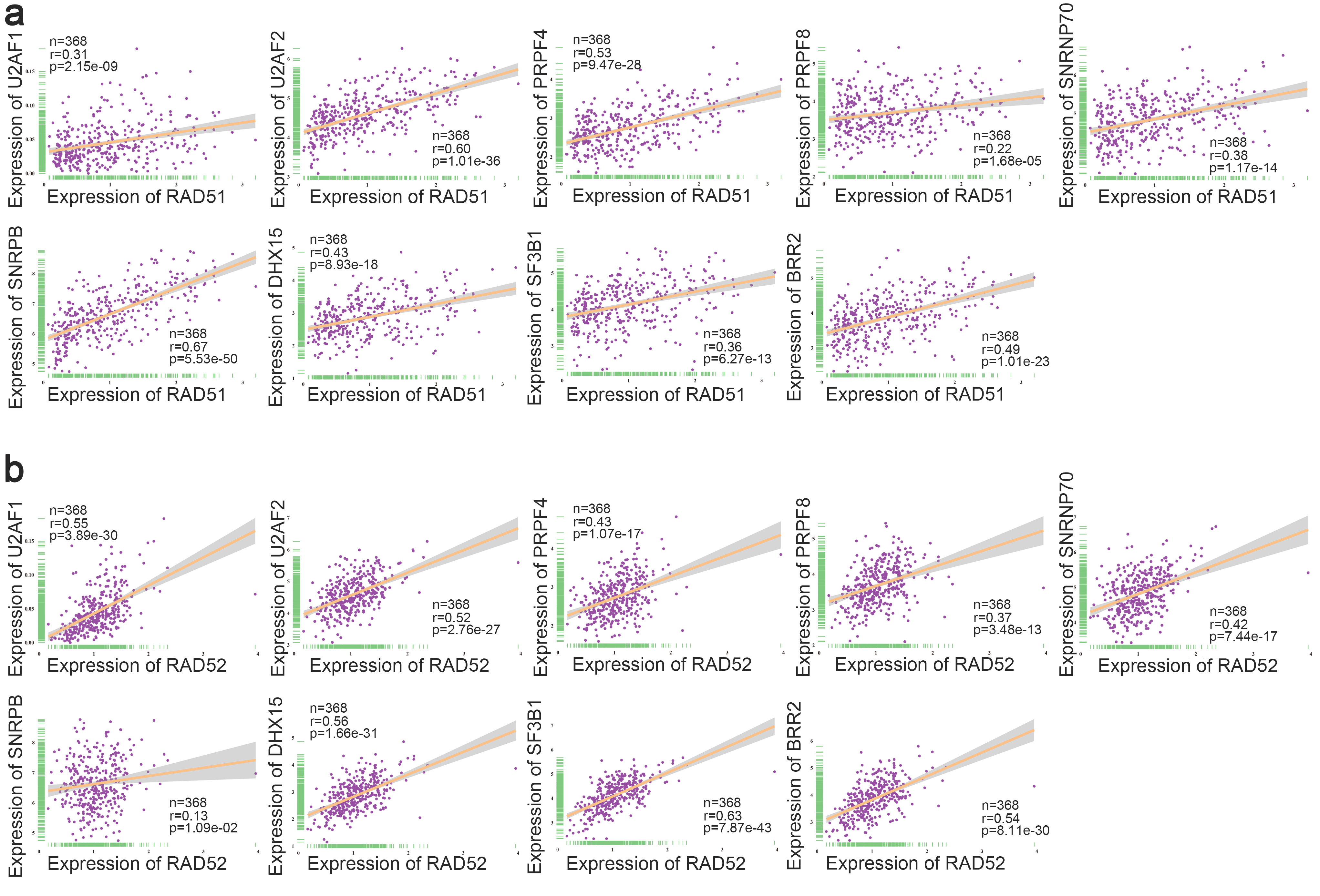


**Supplementary Figure 8 KAT2A-mediated K419 succinylation of SRSF11 promote spliceosome formation. a.** Correlation analysis of the protein expression of RAD51 with U2AF1, U2AF2, PRPF4, PRPF8, SNRNP, SNRPB, DHX15, SF3B1 and BRR2 from TCGA-LIHC database. **b**. Correlation analysis of the protein expression of RAD52 with U2AF1, U2AF2, PRPF4, PRPF8, SNRNP, SNRPB, DHX15, SF3B1 and BRR2 from TCGA-LIHC database.


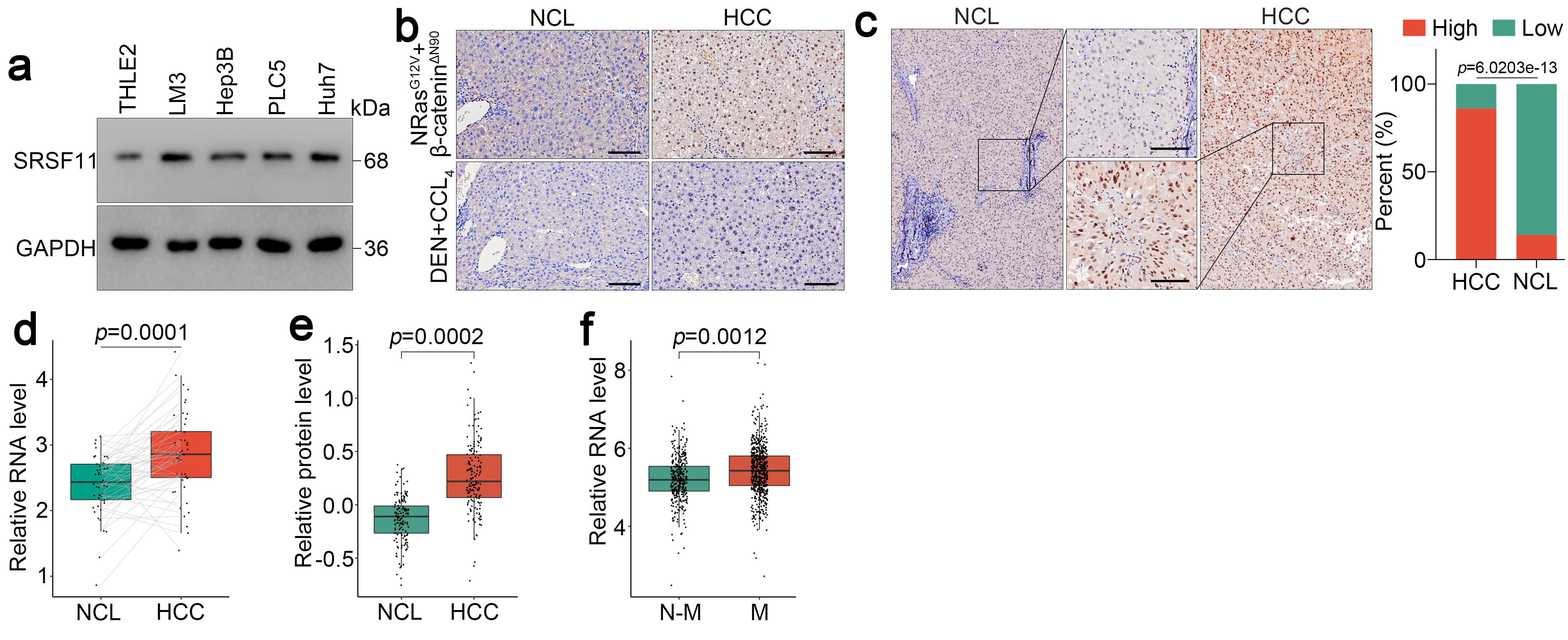


**Supplementary Figure 9** **SRSF11 correlates with HR-mediated DSBs repair, radioresistance and poor survival in HCC. a.** Western blotting assay of SRSF11 expression in Huh7, LM3, Hep3B, PLC5 and THLE2 cells. **b**. Representative images of IHC staining with anti-SRSF11 antibody in DEN and β-catenin^ΔN90^-induced orthotopic mouse HCC models. Scale bars, 100 μm. **c**. Representative images of IHC staining with anti-SRSF11 antibody in HCC and NCL tissues from clinical samples of HCC patients (Number of samples: 50). Scale bars, 100 μm. **d, e**. The SRSF11 expression in RNA (**d**) and protein (**e**) level by transcriptomic and proteomic data of TCGA-LIHC. **f**. The SRSF11 expression in HCC cells and non-HCC cells using single-cell transcriptome data (GSE166635). For a, 3 independent experiments (n = 3) with similar results were performed in triplicate. For a and d‒f the data are presented as the means ± SDs. The statistical analyses were performed via two-tailed unpaired Student’s t-tests. p < 0.05 was considered to indicate statistical significance.


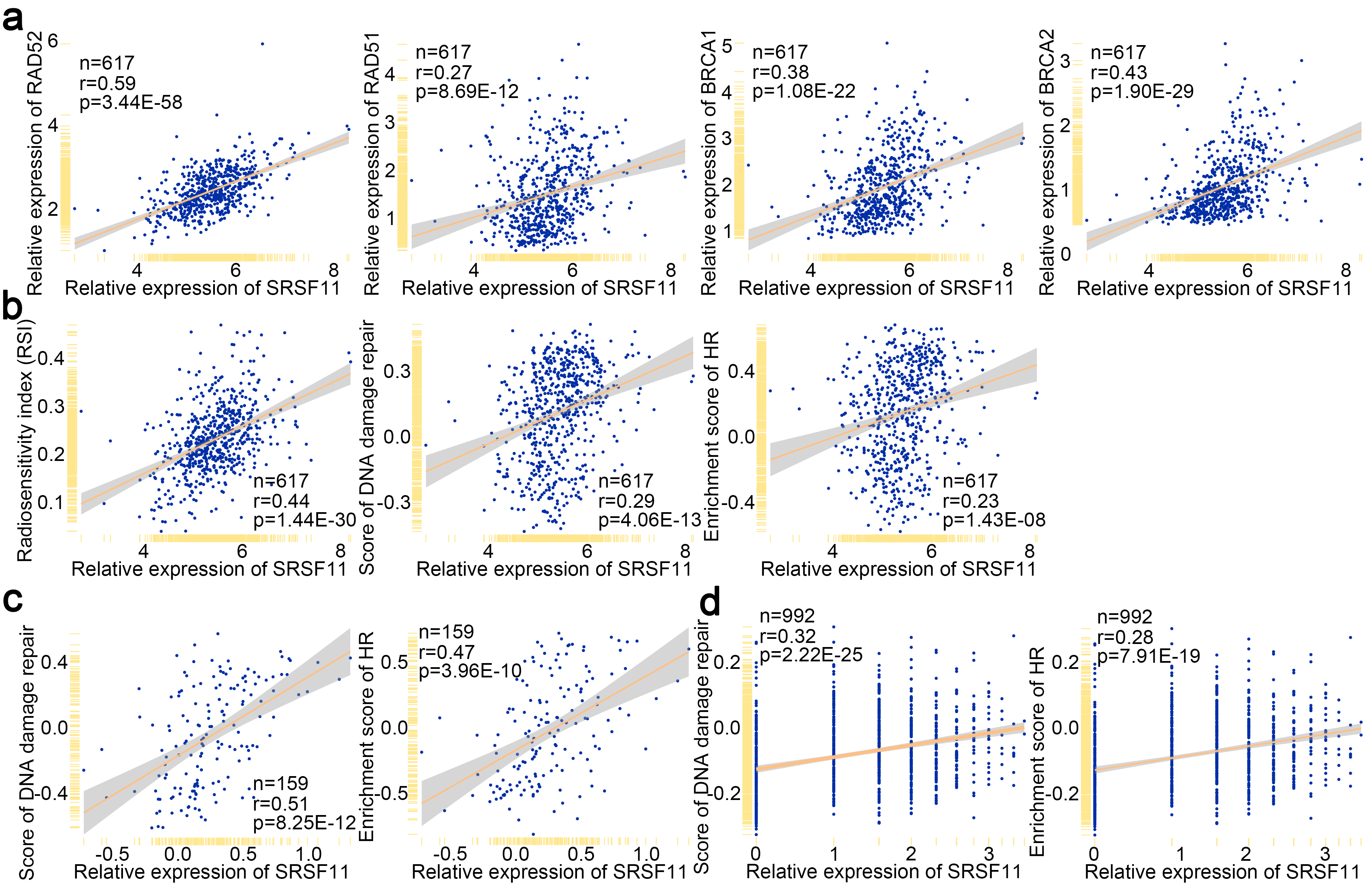


**Supplementary Figure 10 SRSF11 correlates with HR-mediated DSBs repair and radioresistance. a**. Correlation analysis of SRSF11 expression with RAD51, RAD52, BRCA1 and BRCA2 by transcriptomic or proteomic data from TCGA-LIHC database. **b**. Correlation analysis of SRSF11 expression with radiotherapy index, score of DNA damage repair and enrichment score of HR using single-cell transcriptome data (GSE166635). **c, d**. Correlation analysis of SRSF11 expression at the RNA (**d**) and protein (**e**) levels with the DNA damage repair score and enrichment score of HR from the TCGA-LIHC database. p < 0.05 was considered to indicate statistical significance.

**
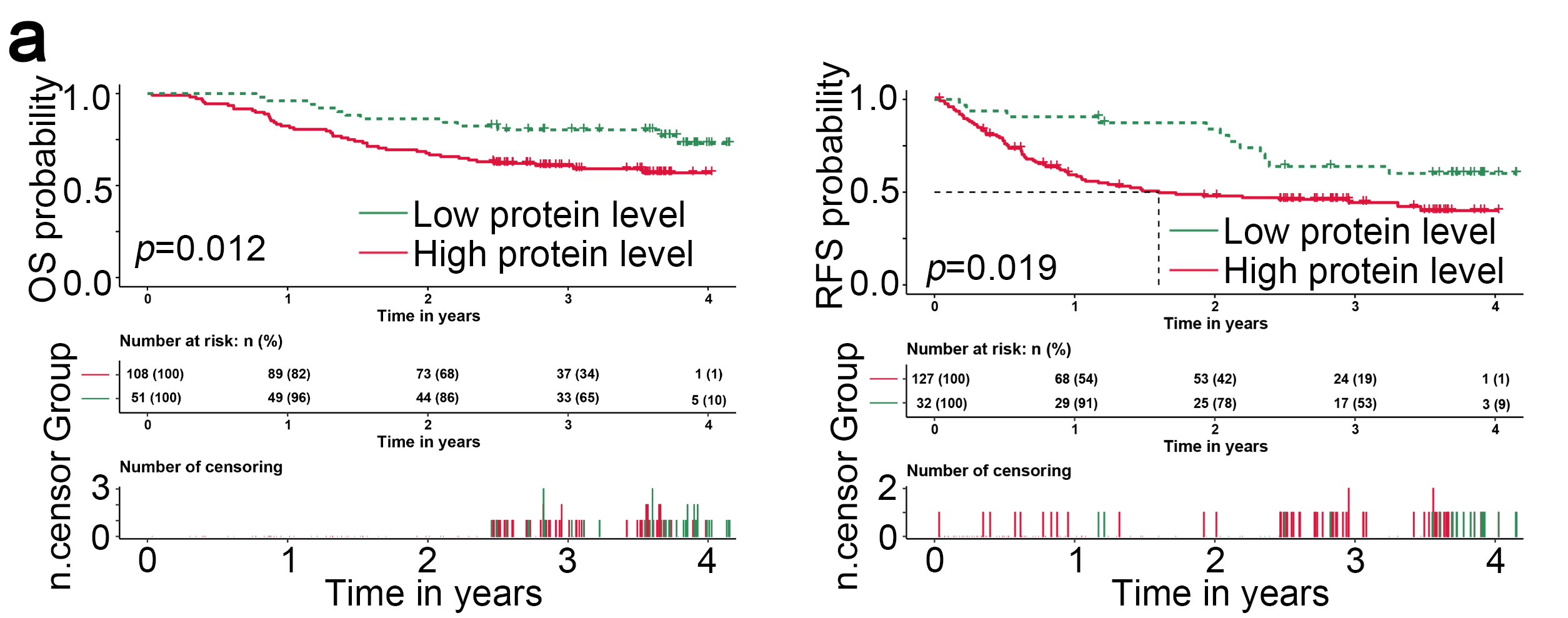
**

**Supplementary** **Figure 11 SRSF11 correlates with poor survival in HCC. a**. OS and RFS curves using proteomic data from TCGA-LIHC database. p < 0.05 was considered to indicate statistical significance.

**Supplementary Table 2 SRSF11 expression and the clinical characteristics of patients with HCC.**

| Clinical features | | N | SRSF11 expression | |
| --- | --- | --- | --- | --- |
|  |  |  | High | Low |
| Gender | Male | 41 | 20 | 21 |
|  | Female | 9 | 4 | 5 |
| Age (years) | >55 | 23 | 10 | 13 |
|  | ≤55 | 27 | 14 | 13 |
| BMI | >24 | 27 | 15 | 12 |
|  | ≤24 | 23 | 9 | 14 |
| Height (cm) | >165 | 25 | 12 | 13 |
|  | ≤165 | 25 | 12 | 13 |
| Weight (kg) | >60 | 26 | 16 | 10 |
|  | ≤60 | 24 | 8 | 16 |
| HBV | Positive | 38 | 20 | 18 |
|  | Negative | 12 | 4 | 8 |
| History of alcoholism | With | 12 | 6 | 6 |
|  | Without | 38 | 18 | 20 |
| Cirrhosis | With | 38 | 19 | 19 |
|  | Without | 12 | 5 | 7 |
| BCLC stage | A+B | 28 | 9 | 19 |
|  | C | 22 | 15 | 7 |
| AJCC stage | I-II | 27 | 8 | 19 |
|  | III | 23 | 16 | 7 |
| Tumor size (cm) | >5 | 34 | 20 | 14 |
|  | ≤5 | 16 | 4 | 12 |
| Tumor number | 1 | 30 | 12 | 18 |
|  | >1 | 20 | 12 | 8 |
| Differentiation | G1-G2 | 24 | 8 | 16 |
|  | G3 | 26 | 16 | 10 |
| MVI | Present | 12 | 8 | 4 |
|  | Absent | 38 | 16 | 22 |
| Satellite lesions | Present | 8 | 5 | 3 |
|  | Absent | 16 | 7 | 9 |
| AFP (ng/ml) | Normal | 10 | 4 | 6 |
|  | Abnormal | 40 | 20 | 20 |

The expression of SRSF11 in HCC samples were classified into high expression (IHC score>5) and low expression (IHC score<5) groups based on IHC scores.

**Supplementary Table 3 Sequences of the siRNA targeting sequence used in the study.**

| Primer names | Targeting sequence (5’-3’) |
| --- | --- |
| siSRSF11-1 | GAGCAAACTTGAACTCTCA |
| siSRSF11-2 | GGCACAAGATCTCCTAAAA |
| siKAT2A-1 | CGAAACCACTCATGTCTTT |
| siKAT2A-2 | GCCATCGGCTACTTCAAAA |
| siRAD52(exon 10)-1 | GGAGAAAGACTTCCTTGCA |
| siRAD52(exon 10)-2 | GCACAGCACTCCTGTAACT |
| siRAD52(exon 10)-3 | GGAGTGACTCAAGAATTAA |

**Supplementary Table 4 Sequences of the shRNA targeting sequence used in the study.**

| Sequence names | Targeting sequence (5’-3’) |
| --- | --- |
| shSRSF11-1 sense | GCATCTGACAAACACTGTATT |
| shSRSF11-1 antisense | AATACAGTGTTTGTCAGATGC |
| shSRSF11-2 sense | GCCTACTCCTAACCCACTTAC |
| shSRSF11-2 antisense | GTAAGTGGGTTAGGAGTAGGC |
| shRAD52-1 sense | GCTGGGCCCAGAATACATAAG |
| shRAD52-1 antisense | CTTATGTATTCTGGGCCCAGC |
| shRAD52-2 sense | GCAGGAGTGACTCAAGAATTA |
| shRAD52-2 antisense | TAATTCTTGAGTCACTCCTGC |

**Supplementary Table 5 Information of antibodies used in this study.**

| Antibody names | Manufacturer | Catalog Number | Application |
| --- | --- | --- | --- |
| Anti-pan-Ksu antibody | PTM Biolab | PTM-419 | 1:1000 for WB; 3μg for CoIP |
| Anti-SRSF11-K419su antibody | HuaBio | - | 1:1000 for WB; 1:50 for IHC |
| Anti-SRSF11 antibody | Abcam | Ab254733 | 1:1000 for WB; 3μg for CoIP; 1:200 for IHC, IF, and PLA; 3μg for RIP |
| Anti-KAT2A antibody | Proteintech | 66575-1-Ig | 1:2000 for WB; 3μg for CoIP; 1:400 for IF and PLA |
| Anti-Ku70 antibody | Proteintech | 10723-1-AP | 1:3000 for WB; 1:500 for IHC; 1:500 for IF |
| Anti-Ku80 antibody | Proteintech | 16389-1-AP | 1:1000 for WB; 1:600 for IHC; 1:500 for IF |
| Anti-CTIP antibody | Active Motif | 61142 | 1:1000 for WB |
| Anti-RAD51 antibody | Proteintech | 14961-1-AP | 1:1000 for WB; 3μg for CoIP |
| Anti-RAD51 antibody | HuaBio | ET1705-96 | 1:500 for IHC; 1:200 for IF |
| Anti-RAD52 antibody | Proteintech | 28045-1-AP | 1:3000 for WB; 3μg for CoIP |
| Anti-RAD52 antibody | Immunoway | YT3969 | 1:200 for IHC; 1:500 for IF |
| Anti-BRCA1 antibody | Immunoway | YT0519 | 1:1000 for WB; 1:200 for IHC |
| Anti-BRCA2 antibody | Abclonal | A2435 | 1:1000 for WB |
| Anti-γH2AX antibody | Abcam | Ab81299 | 1:250 for IF |
| Anti-PRPF8 antibody | Proteintech | 11171-1-AP | 1:1000 for WB |
| Anti-SNRNP70 antibody | Abclonal | A14786 | 1:1000 for WB |
| Anti-U2AF2 antibody | HuaBio | JE36-33 | 1:1000 for WB |
| Anti-Flag-tag antibody | Proteintech | 66008-4-Ig | 1:5000 for WB; 3μg for CoIP, 3μg for RIP |
| Anti-HA-tag antibody | Proteintech | 51064-2-AP | 1:5000 for WB; 3μg for CoIP |
| Anti-IgG antibody | Proteintech | 98136-1-RR | 3μg for CoIP; 3μg for RIP |
| Anti-GAPDH antibody | Proteintech | 60004-1-Ig | 1:100000 for WB |
| HRP-conjugated Goat anti-Rabbit antibody | Proteintech | SA00001-2 | 1:5000 for WB |
| HRP-conjugated Goat anti-mouse antibody | Proteintech | SA00001-1 | 1:5000 for WB |

WB, western blotting; CoIP, co-immunoprecipitation; IF, immunofluorescence; IHC, immunohistochemistry; RIP, RNA immunoprecipitation; PLA: *In situ* Proximity Ligation Assay.

**Supplementary Table 6 Sequences of the primers for exon skipping detection used in the study.**

| Primer names | Sequences (5’-3’) |
| --- | --- |
| RAD50 forward | ACAGCTTGAAGACTGGCTACAT |
| RAD50 reverse | TGTCGTTCTTTAGGCGCTGT |
| RAD51 forward | GCAGATGCAGCTTGAAGCAA |
| RAD51 reverse | CATACCTCTCAGCCACTGCC |
| RAD51B forward | CCTTACCTGTCAGGACTTTTTATGTC |
| RAD51B reverse | CAATCCTTTGTAGAACTTCATCAC |
| RAD52 forward | AGAAGCAGCAGGTTCGAGTC |
| RAD52 reverse | GATTCCCAGTTTCCTGTTGTGC |
| RAD54B forward | ACCTCCAGGAAGAAGTAATCCAG |
| RAD54B reverse | ATCAGTGTTTGGCCCTCTTCA |
| RAD54L forward | GCCTAGTGACTCCTAGGAAACGG |
| RAD54L reverse | GACTCCAACATGAAGGCGGA |
| RBM6 forward | CTTGTTGGGGCCCTCTTGAT |
| RBM6 reverse | CCTGCTGGGTTTCTCCTCAC |
| GAPDH forward | GGAGTCCACTGGCGTCTTCA |
| GAPDH reverse | GTCATGAGTCCTTCCACGATACC |

**Uncropped western blots**


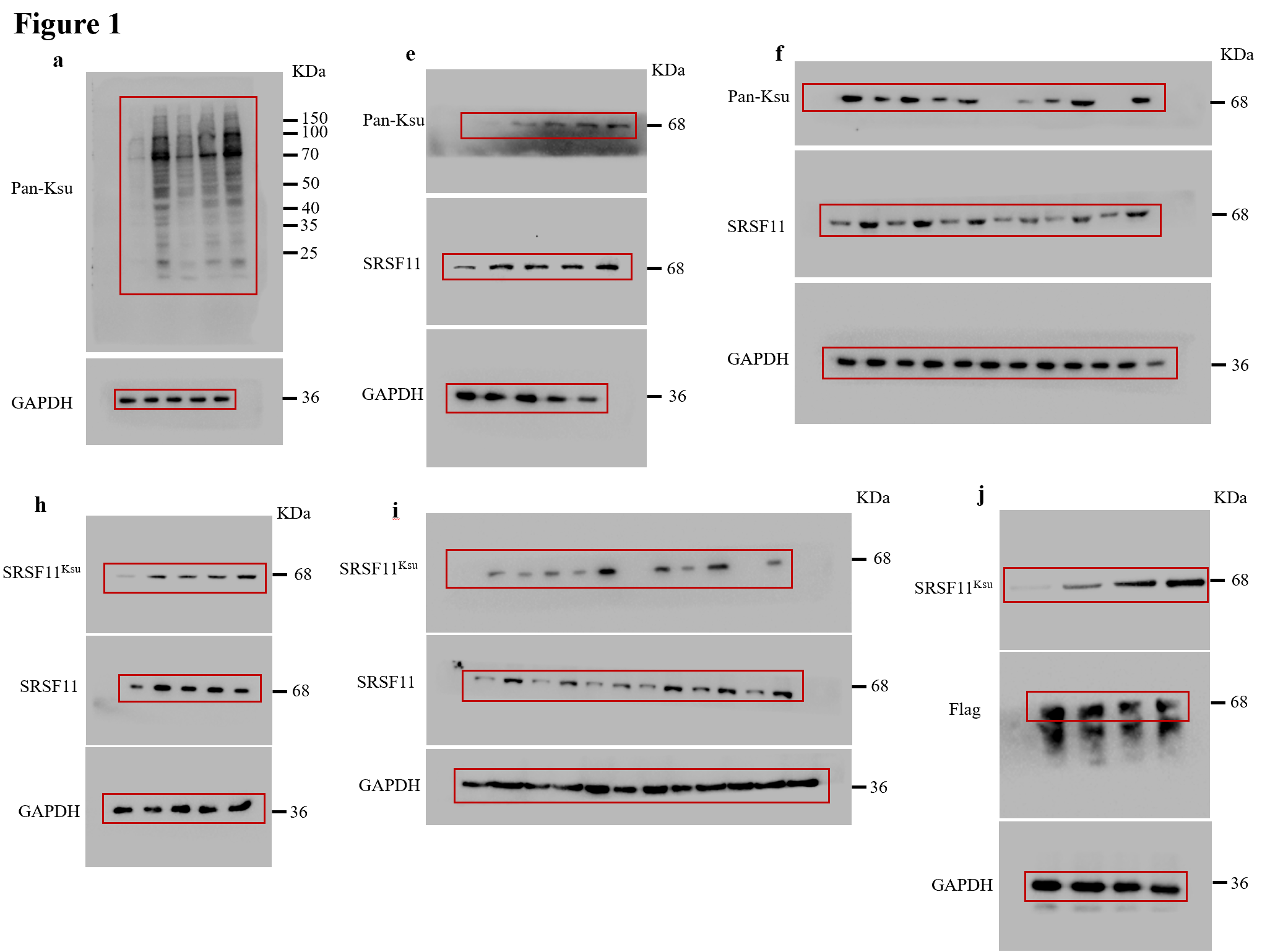


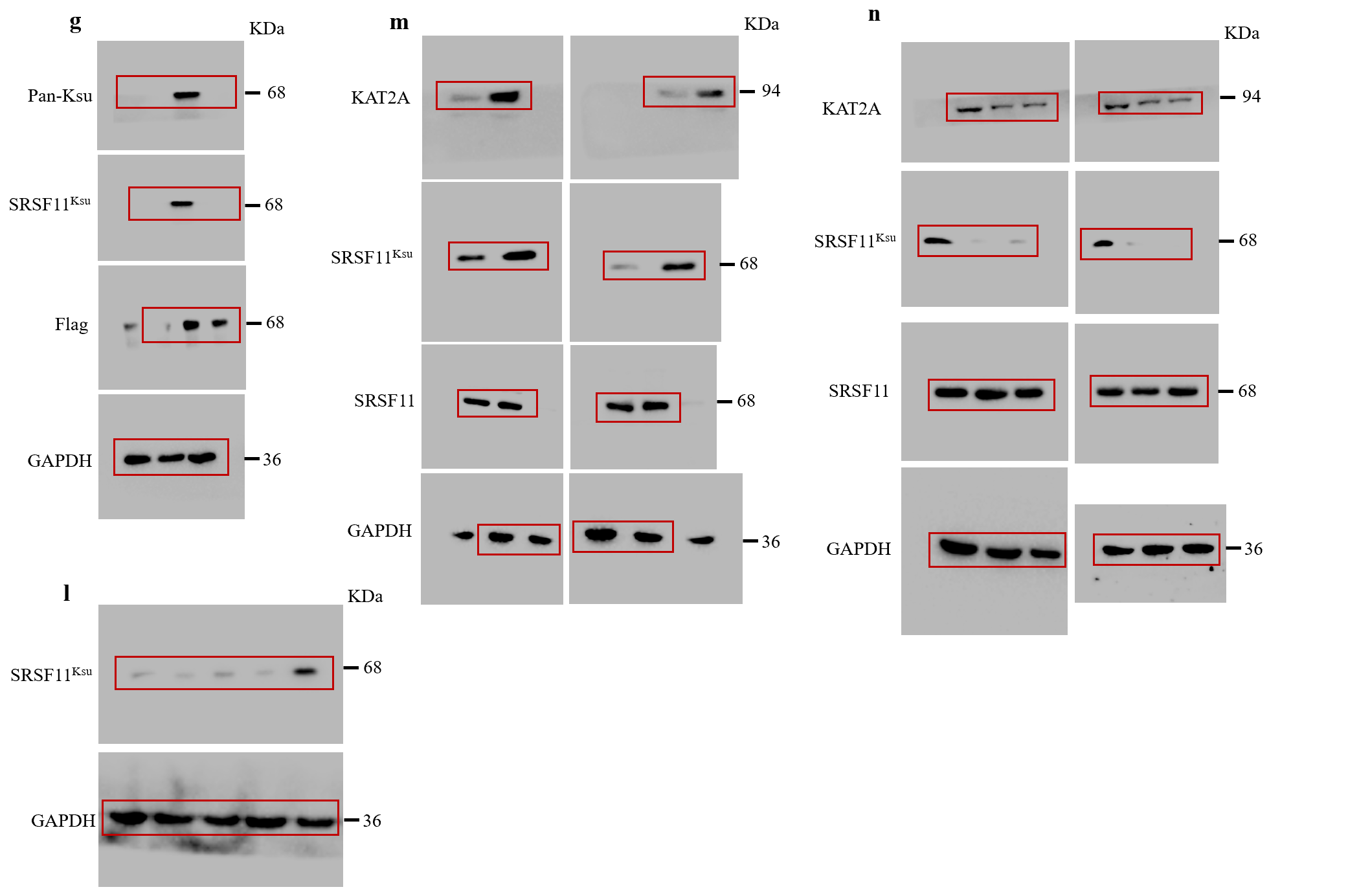


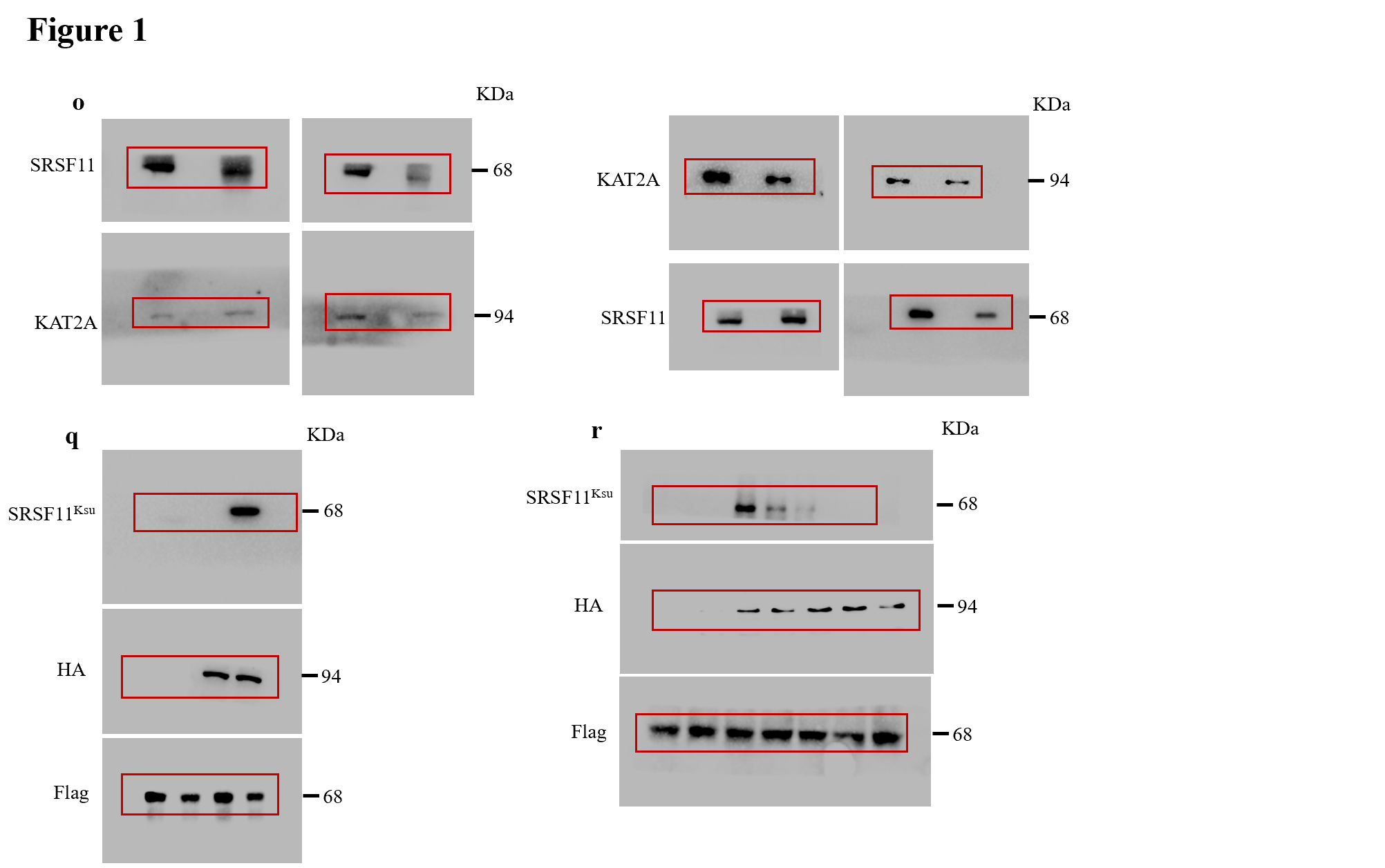


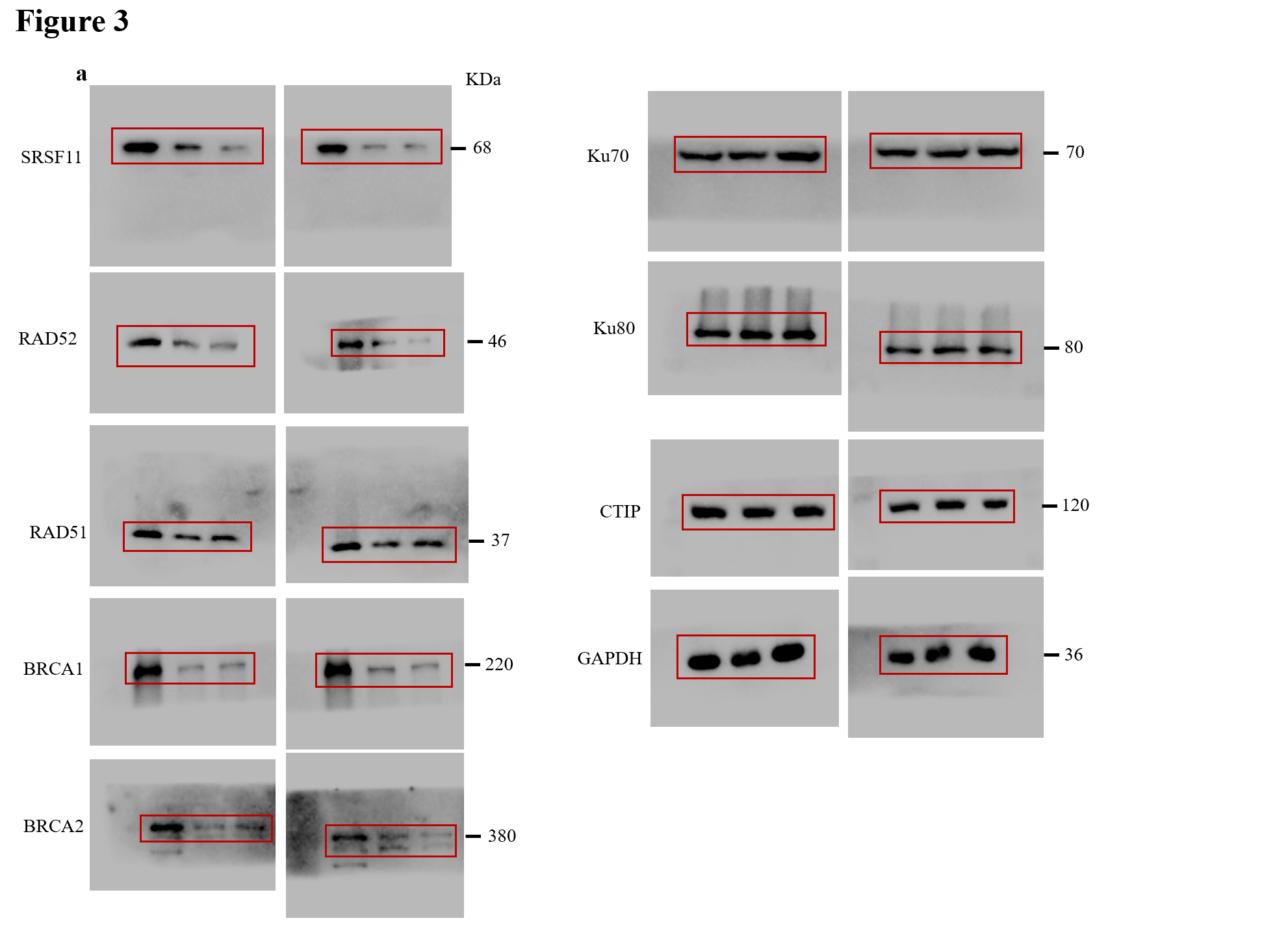


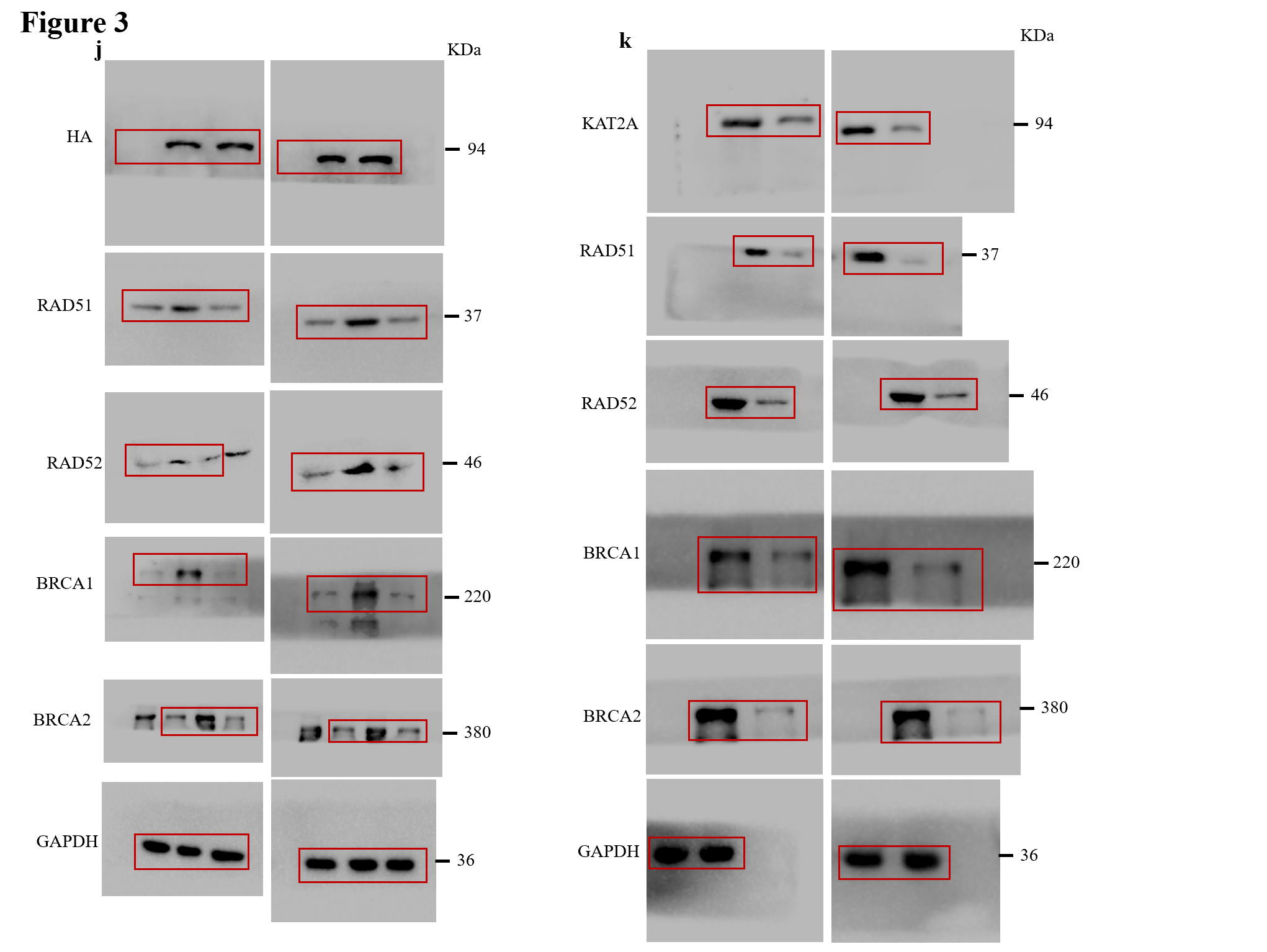


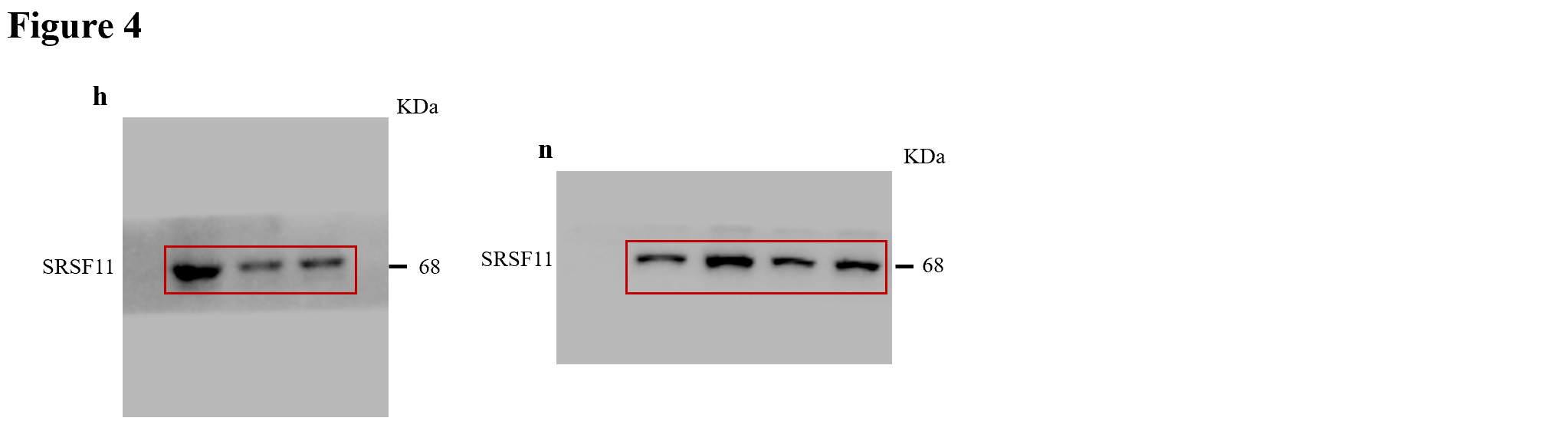


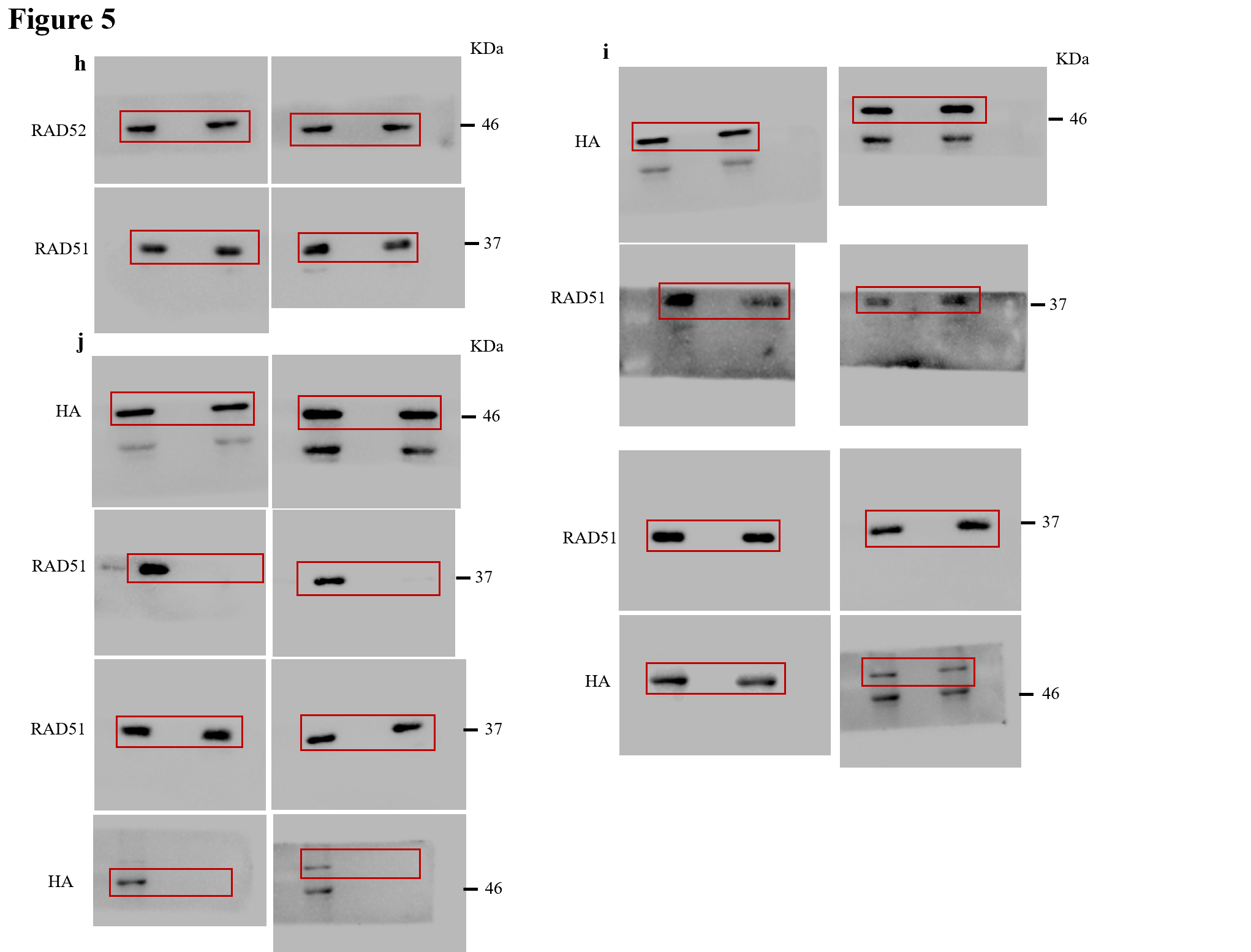


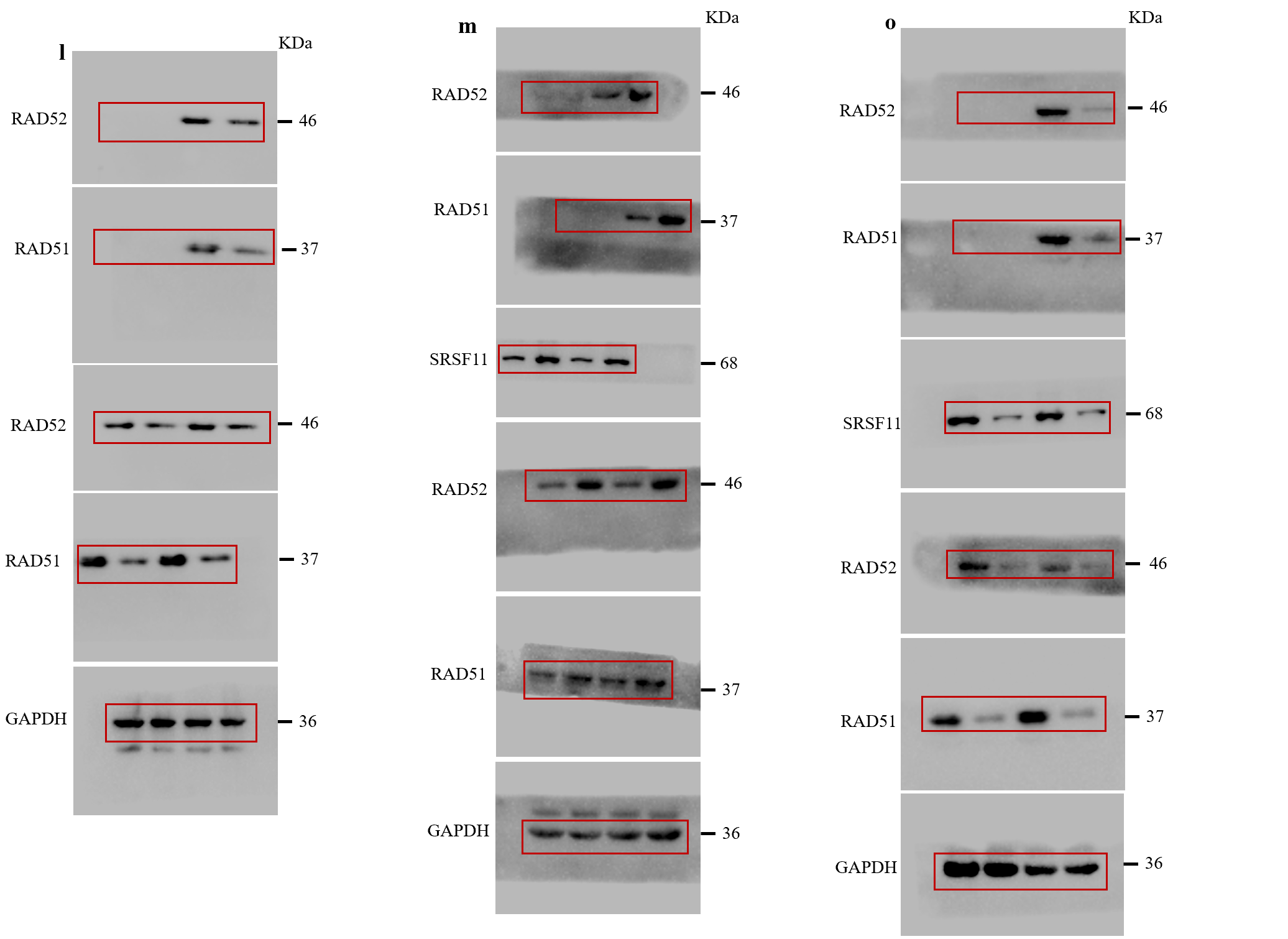


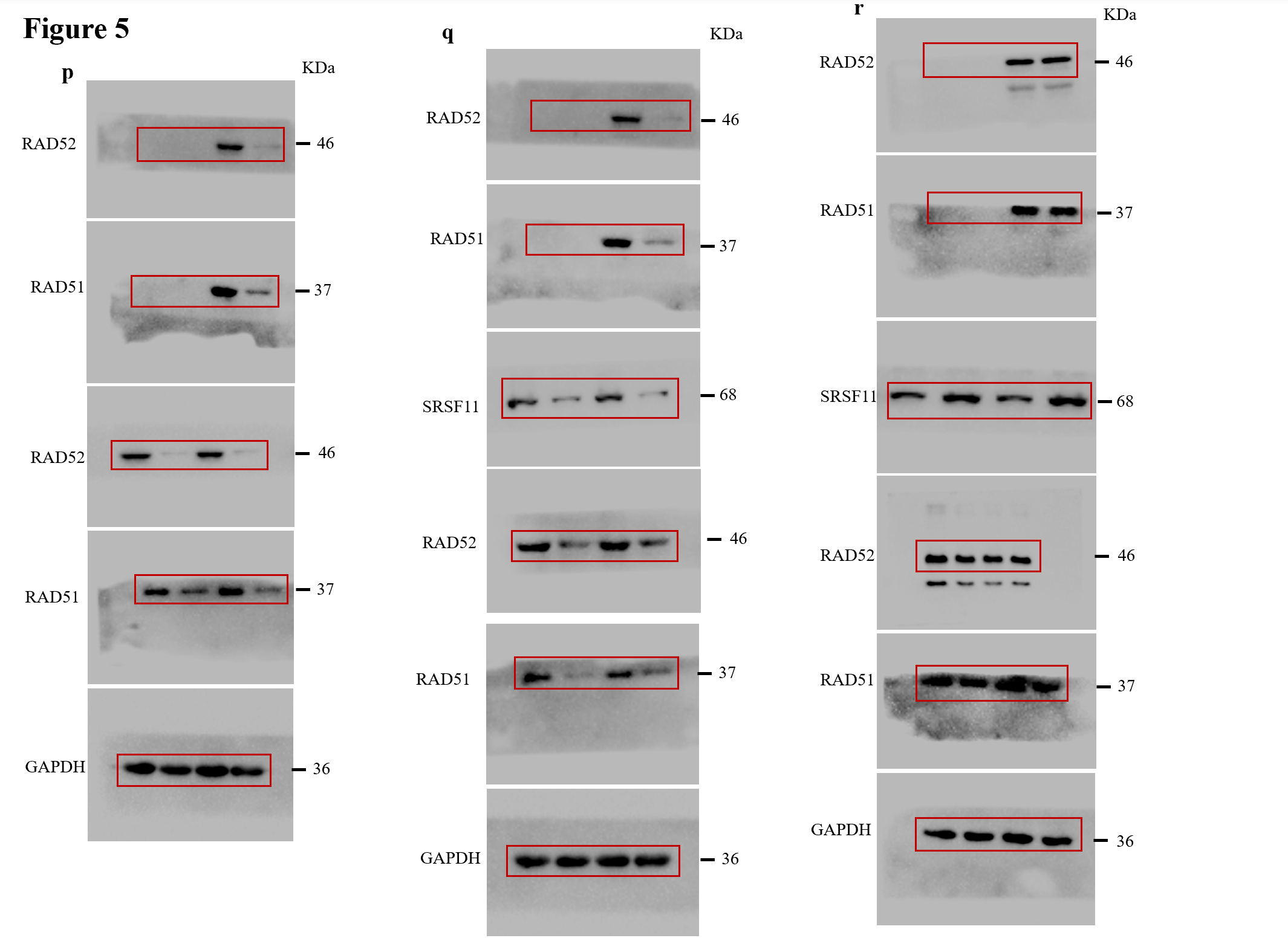


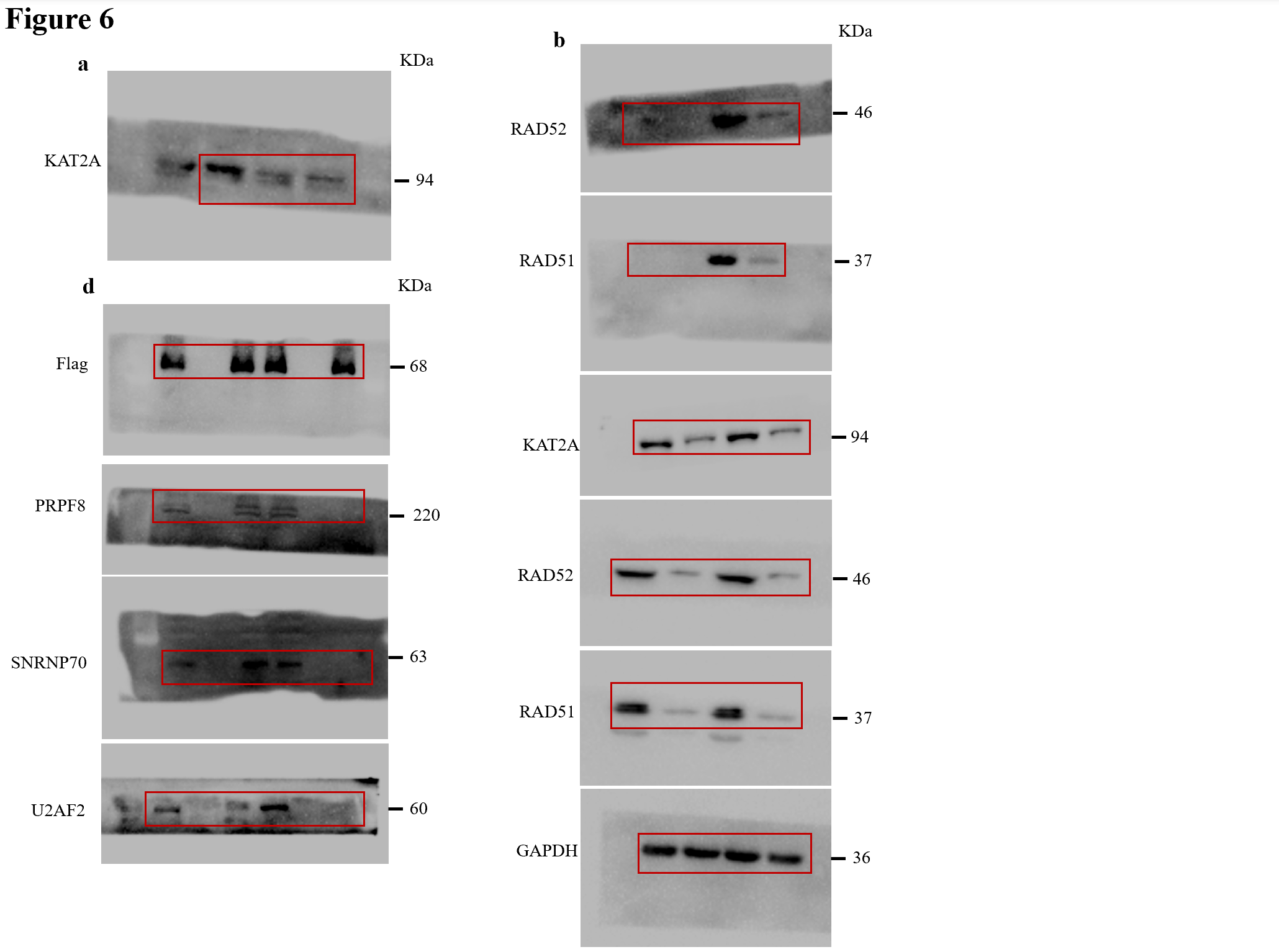


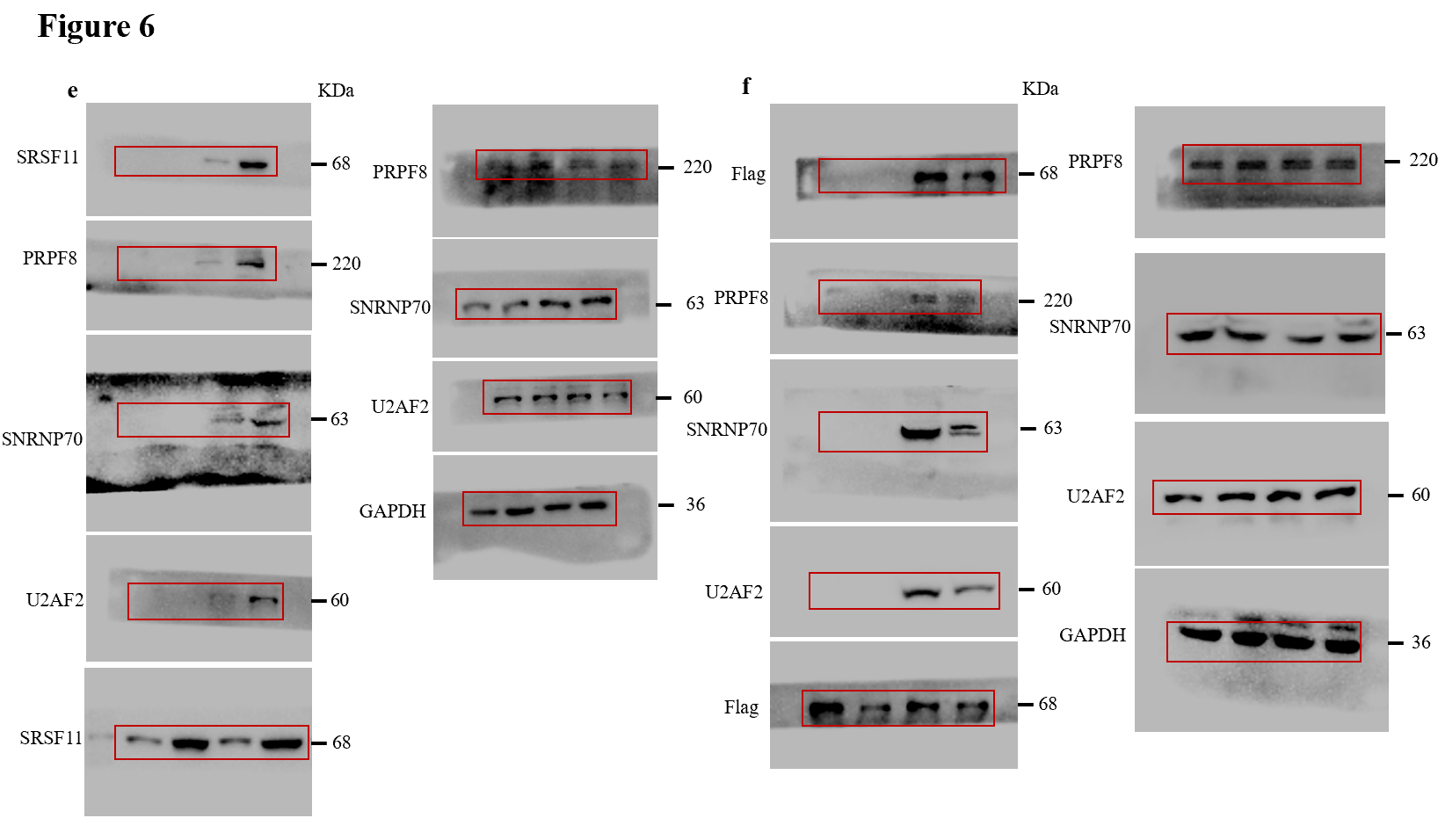


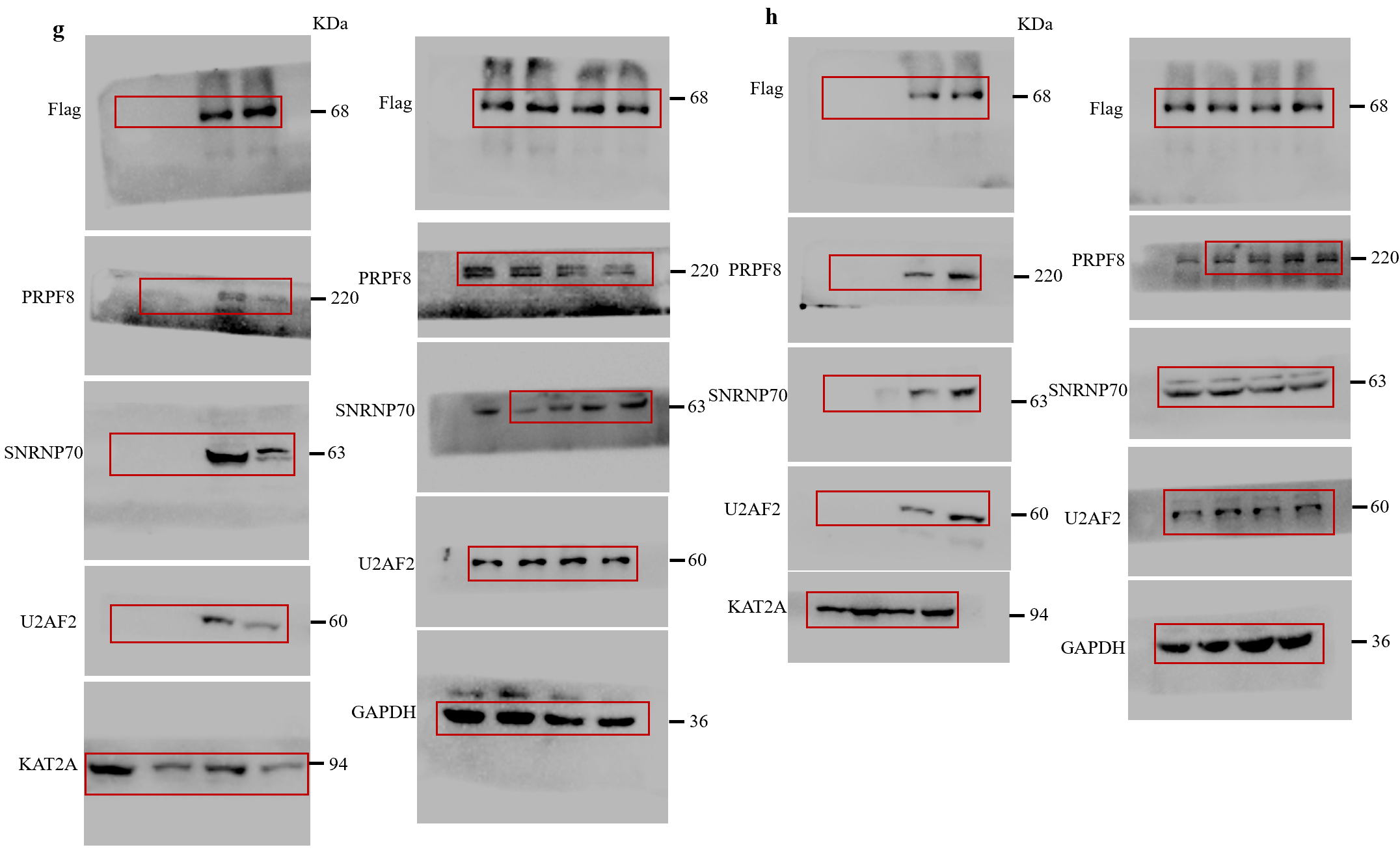


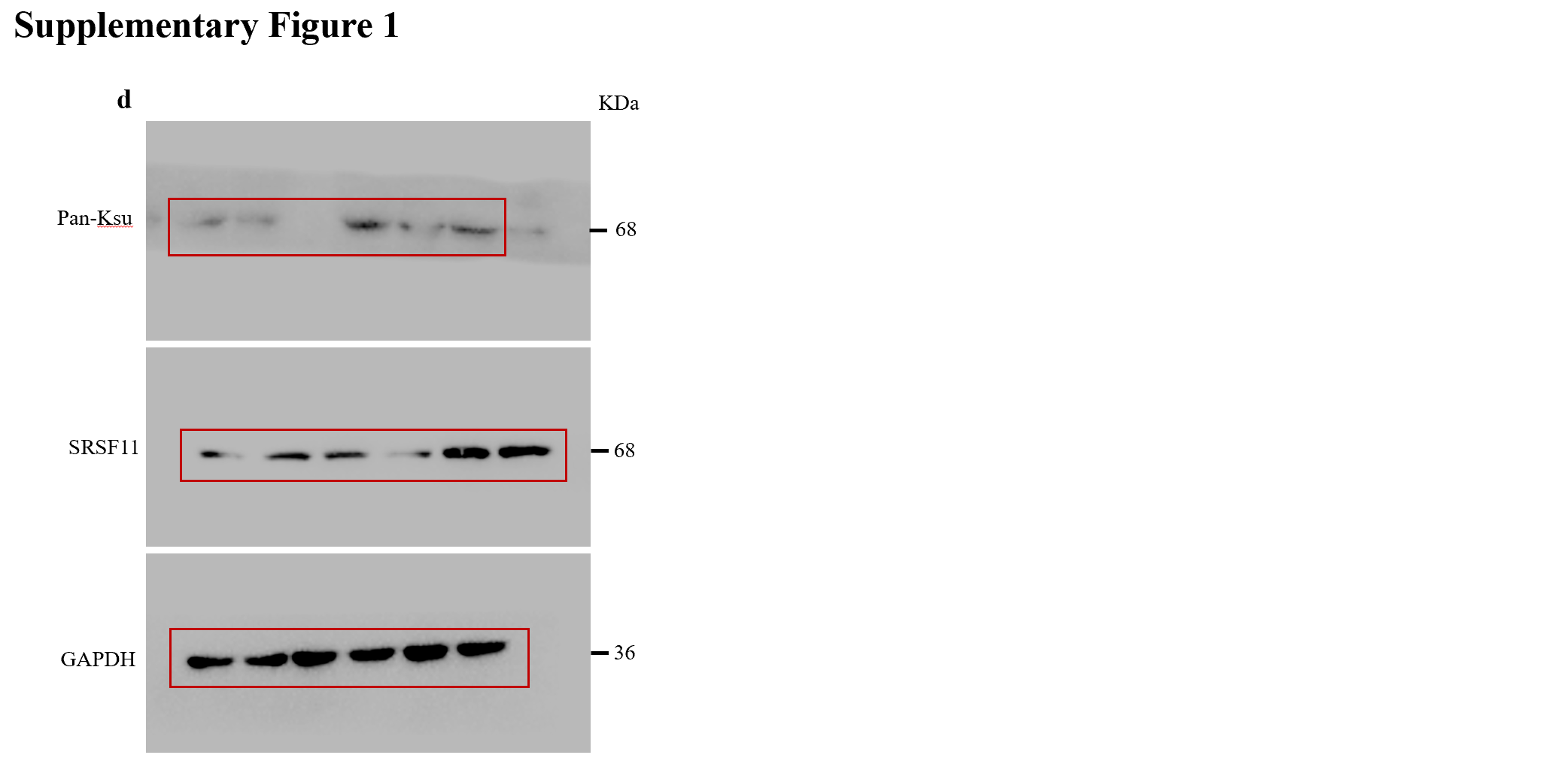


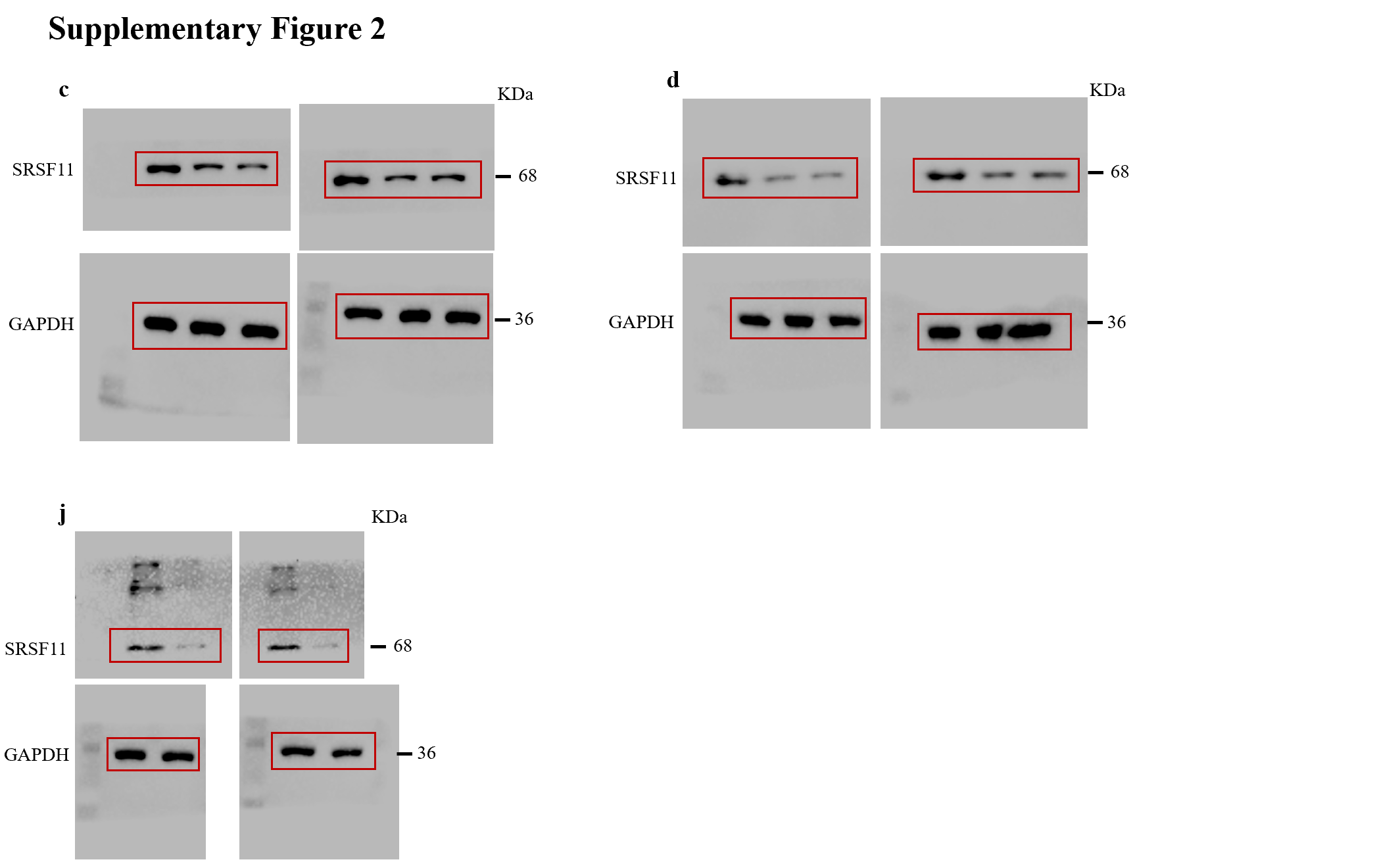


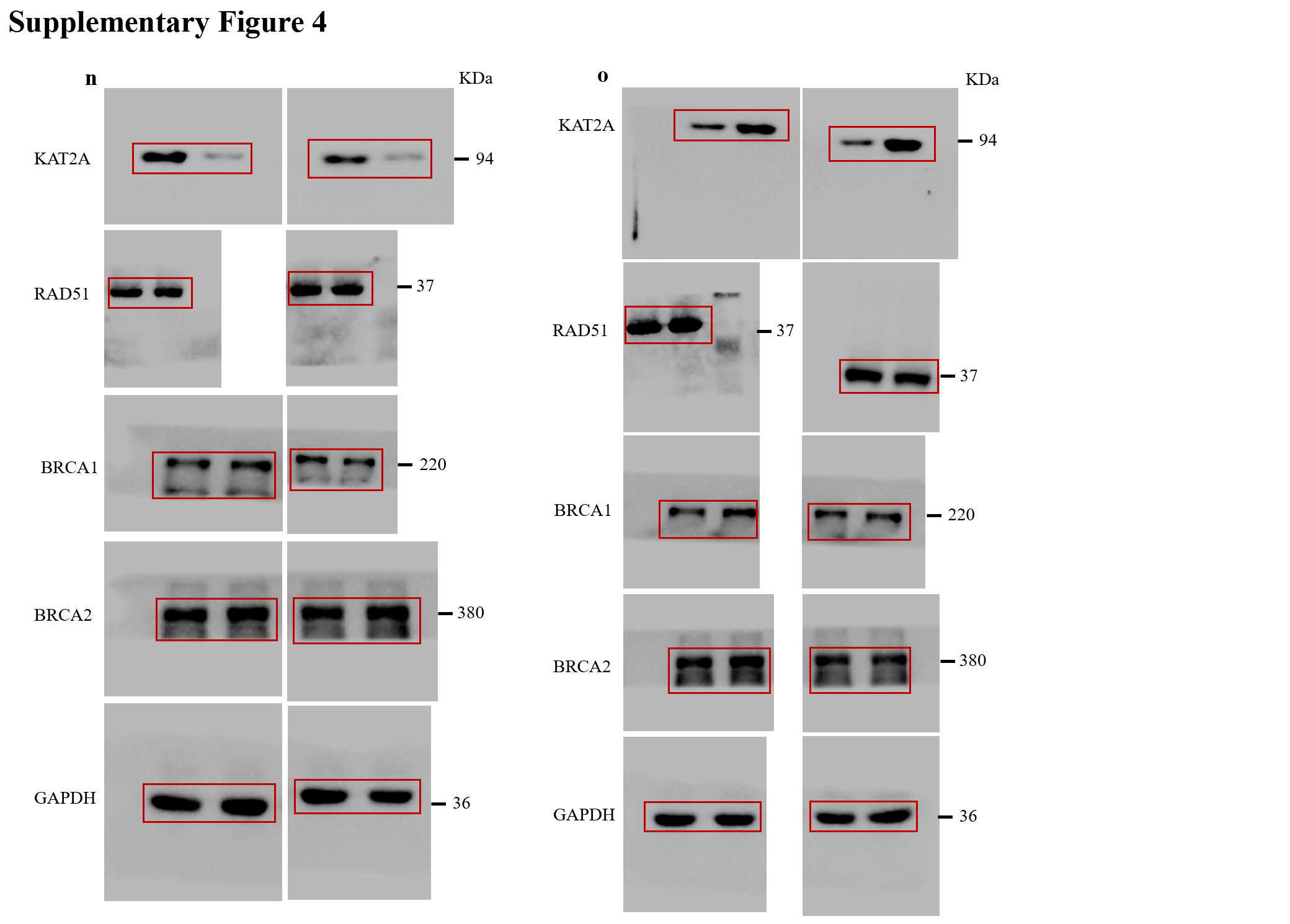


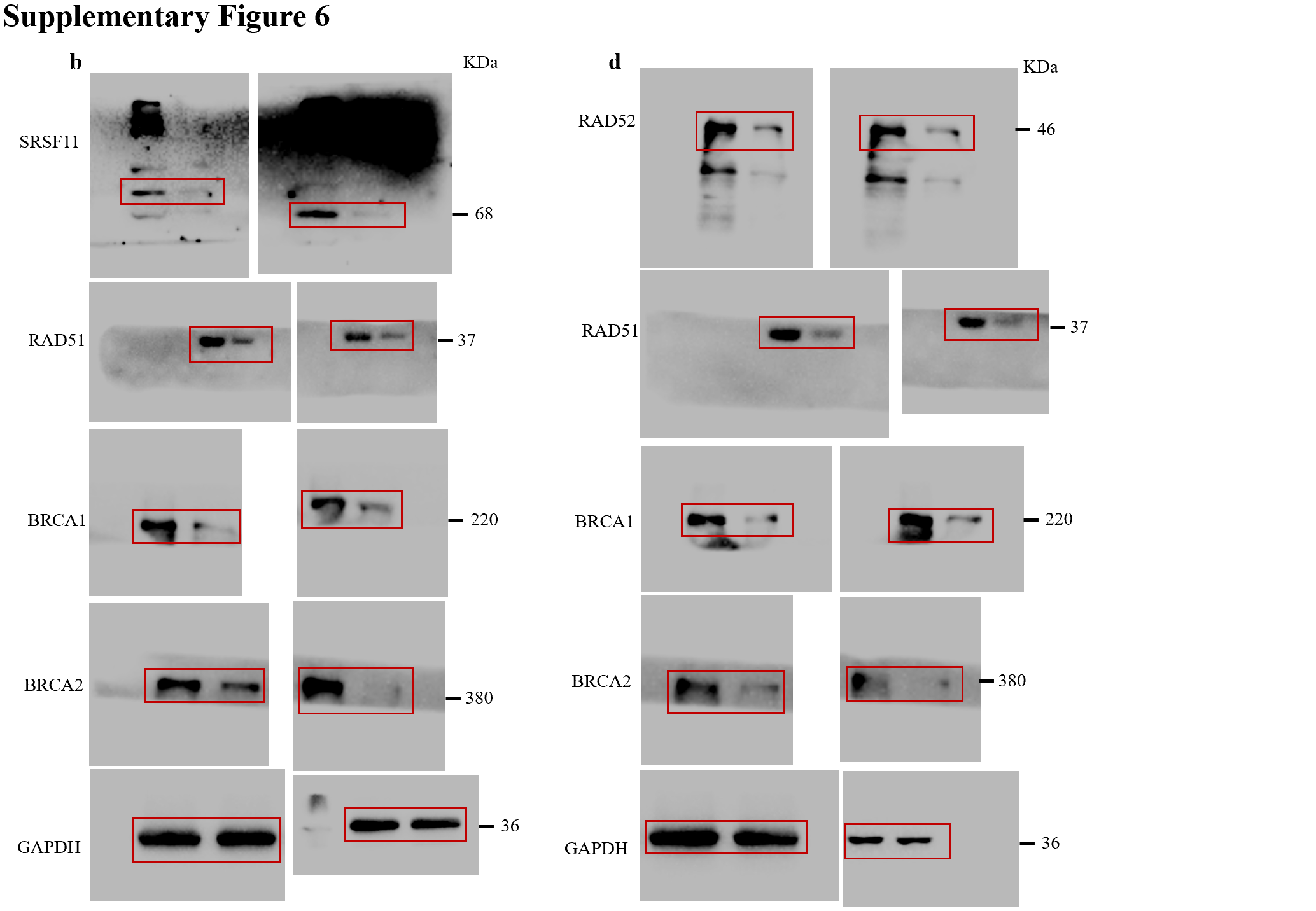


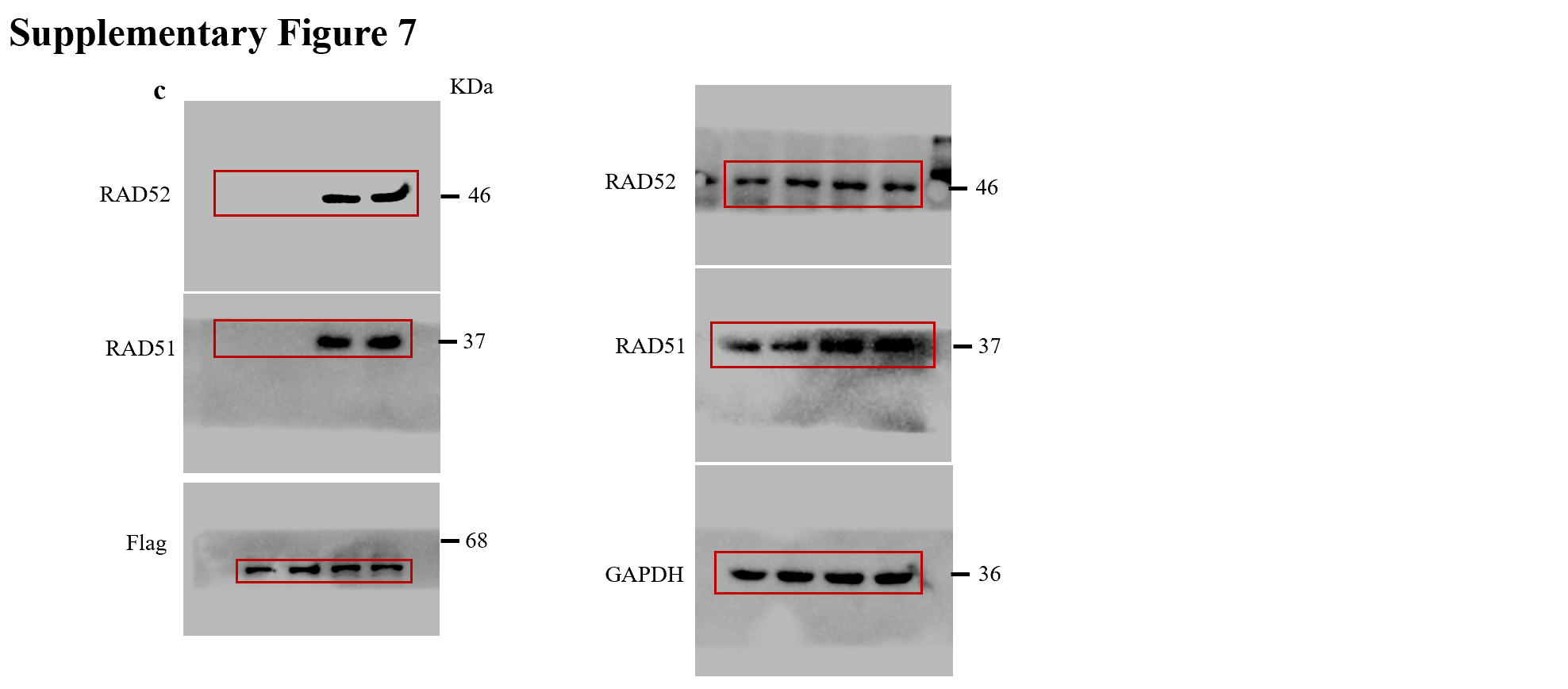


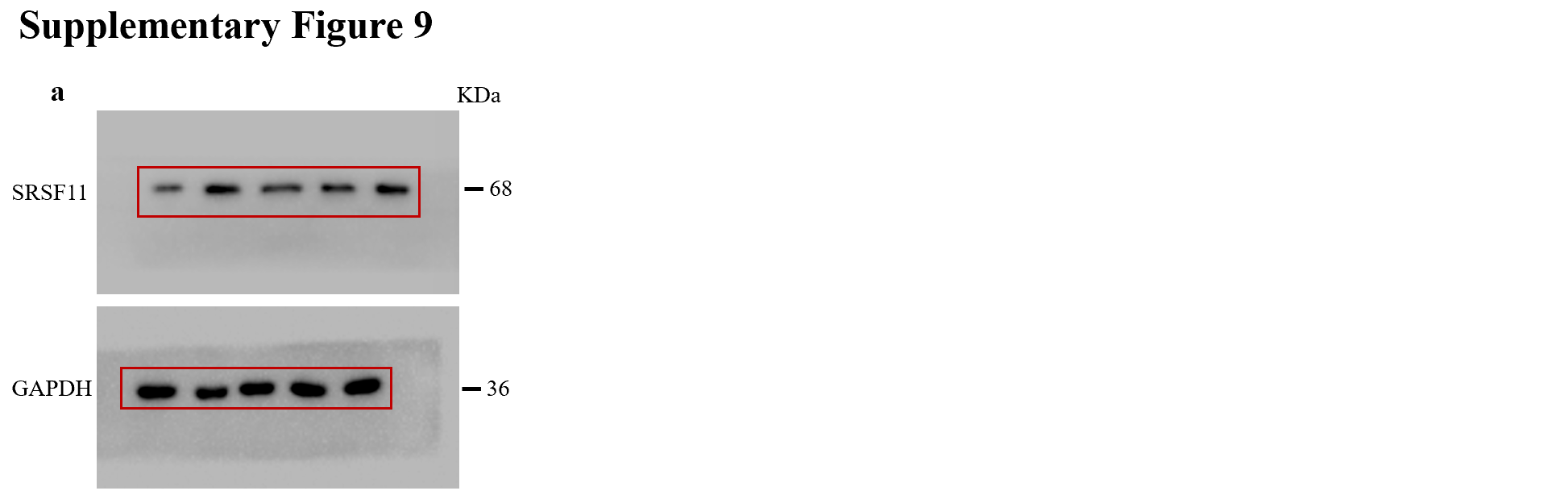

Supplement: Supplementary file 1 — Supplementary Materials [file 41392_2025_2458_MOESM1_ESM.docx]
